# Role of Coordination Environment in Synergistic Catalysis: A Molecular Orbital Perspective on M_1_M_2_N_6_ Catalysts

**DOI:** 10.1021/jacs.6c08871

**Published:** 2026-07-16

**Authors:** Zhangsheng Shi, Ziyang Wu, Zheng Shu, Xin Wang

**Affiliations:** 1 Department of Chemistry, 53025 City University of Hong Kong, Hong Kong SAR 999077, PR China; 2 State Key Laboratory of Advanced Fiber Materials, College of Materials Science and Engineering, Donghua University, Shanghai 201620, China; 3 Department of Chemistry, National University of Singapore, Singapore 117543, Republic of Singapore

## Abstract

Visualizing the coordination environment-dependent synergistic mechanisms that govern the structural stability and adsorption behavior of dual-atom catalysts (DACs) is pivotal for precise catalyst design. However, insights into these mechanisms at the molecular orbital level remain elusive. Herein, we present large-scale density functional theory calculations to elucidate how synergistic effects arise from the combination of *d* atomic orbitals into molecular orbitals between M_1_ and M_2_ sites, with notable variations observed from M_1_–N_3_–M_2_–N_3_–C to M_1_–N_4_–M_2_–N_4_–C. We identify an average weakening of M_1_/M_2_–N bond strength in M_1_–N_3_–M_2_–N_3_–C relative to M_1_–N_4_–M_2_–N_4_–C, which is attributed to a shift from direct *d*_x^2^–y^2^_ orbital overlap to nitrogen-mediated interactions involving the hybridization of bridge nitrogen 2*p* orbitals. Using hydrogen as a model adsorbate, we demonstrate that hydrogen adsorption on M_1_–N_3_–M_2_–N_3_–C shifts from wild modulation via a bridge configuration to mild modulation through an end-on configuration, signifying a selective orbital coupling from *d*_x^2^–y^2^_ to *d*_z^2^_ orbitals. In contrast, hydrogen adsorption on M_1_–N_4_–M_2_–N_4_–C exhibits only mild modulation via an end-on configuration. This behavior is ascribed to the symmetry constraints of antibonding (*d*-*d*/d*)-p* molecular orbitals near the Fermi level, mediated by nitrogen-mediated *d*_z^2^_-*d*_z^2^_^*^ molecular orbitals. Furthermore, machine learning analyses corroborate these coordination environment-dependent synergistic mechanisms. Our findings provide a comprehensive molecular orbital-level understanding of how the interplay between coordination environments and electronic structures influences the properties of M_1_M_2_N_6_ catalysts, thereby establishing a theoretical framework for enhanced DACs design.

## Graphical abstract

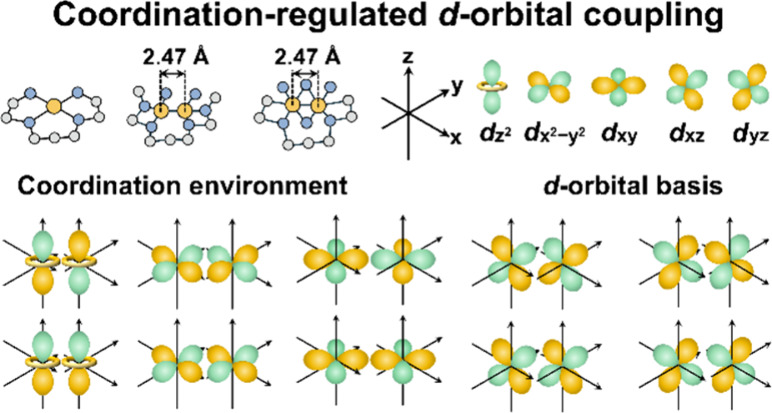

## 1 Introduction

Heterogeneous single-atom catalysts (SACs)^1–4^ have garnered considerable attention due to their advantages, including low cost, maximal atomic utilization, and tunable catalytic properties. Among SACs, those featuring M–N_4_ moieties have been extensively studied for applications such as the hydrogen evolution reaction (HER).^5–8^ However, the monotonous geometric configuration of isolated metal centers in SACs limits the modulation of their electronic structure, thereby constraining further optimization.^9,10^ Dual-atom catalysts (DACs), as an extension of SACs, offer enhanced electronic structure modulation due to their intricate and flexible active sites.^11,12^ This enables DACs to achieve superior structural stability, catalytic activity, and selectivity compared to SACs.^13–16^

Currently, sustainable efforts are devoted to designing DACs with planar-symmetric M_1_M_2_N_6_ atomic configurations, such as M_1_–N_3_–M_2_–N_3_–C and M_1_–N_4_–M_2_–N_4_–C,^17,18^ where the initial intermetallic distances are equal (Figure 1a and Figures S1 and S2). Specifically, Wang et al. introduced a Fe_2_ site into Fe_1_–N_4_–Fe_2_–N_4_–C, enhancing structural stability and enabling direct decomposition of H_2_O_2_ through bridge oxygen adsorption. Additionally, tailoring both geometric and electronic properties via customized coordination environments is another effective strategy to improve the structural stability and adsorption behaviors of DACs.^19,20^ Notably, Li et al. synthesized a Cl-bridged Fe_1_–N_3_–Fe_2_–N_3_–C structure, which strengthens Fe–N bonds while alleviating Fe–O interactions through an end-on configuration, compared to pristine Fe_1_–N_3_–Fe_2_–N_3_–C.^21^ All these strategies are based on introducing or modulating the synergistic cooperation between M_1_ and M_2_ sites, extending beyond SACs. In SACs, the discrete *d* orbitals of metals are selectively coupled by the orbitals of coordinated atoms or the molecular orbital of an adsorbate through end-on or side-on configurations, contributing to their structural stability and chemisorption behaviors.^22–24^ However, for DACs, understanding the synergistic effects within the discrete *d* orbitals of M_1_ and M_2_ sites and how these effects are influenced by the coordination environment is crucial for modulating structural stability, selective orbital coupling (including end-on and bridge adsorption patterns), and for breaking linear scaling relationships in various electrochemical applications.^25^

**Figure 1:**
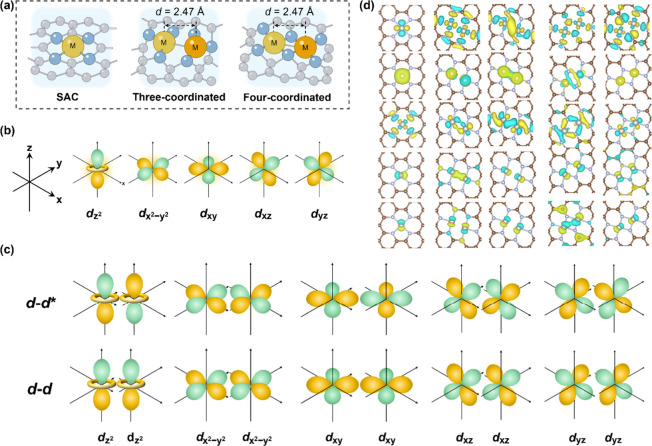
(a) Schematic representations of M–N_4_–C, M_1_–N_3_–M_2_–N_3_–C, and M_1_–N_4_–M_2_–N_4_–C with an intermetallic distance of 2.47 Å. (b) Schematic illustrations of the *d* atomic orbitals of a single metal atom. (c) Schematic illustrations of the *d* molecular orbitals of dual metal atoms. (d) Wave functions of spin-down *d*_x^2^–y^2^_, *d*_z^2^_, *d*_*xy*_, *d*_*xz*_, *d*_*yz*_ orbitals of Ni–N_4_–C, and their coordination environment-dependent molecular orbitals, including *d*_*x* ^2^–*y* ^2^_-*d*_*x* ^2^–*y* ^2^_^*^, *d*_*x* ^2^–*y* ^2^_-*d*_*x* ^2^–*y* ^2^_, *d*_*z* ^2^_-*d*_*z* ^2^_^*^, *d*_*z* ^2^_-*d*_*z* ^2^_, *d*_*xy*_-*d*_xy_^*^, *d*_*xy*_-*d*_*xy*_, *d*_*xz*_-*d*_xz_^*^, *d*_*xz*_-*d*_*xz*_, *d*_*yz*_-*d*_yz_^*^, *d*_*yz*_-*d*_*yz*_ molecular orbitals of Ni–N_3_–Ni–N_3_–C and Ni–N_4_–Ni–N_4_–C (see illustration of * in Figure S6).

While these effects remain abstract, we sought to visualize these synergistic interactions from a molecular orbital perspective by conducting large-scale density functional theory (DFT) calculations on a diverse set of more than 400 SACs and DACs, including M–N_4_–C, M_1_–N_3_–M_2_–N_3_–C, and M_1_–N_4_–M_2_–N_4_–C configurations (see Computational methods and Construction of M_1_M_2_N_6_ in Supporting Information). We compared their thermodynamic stability (α*E*_f_) to explore the impact of synergistic effects between the *d* orbitals of M_1_ and M_2_ on structural stability relative to SACs. Additionally, we investigated how the coordination environment influences these synergistic effects and further affects the structural stability of M_1_–N_3_–M_2_–N_3_–C and M_1_–N_4_–M_2_–N_4_–C configurations. We also examined hydrogen adsorption behavior to understand how adsorption configurations (end-on or bridge adsorption) are influenced by these synergistic effects. The corresponding free energy (Δ*G*_H*_) was calculated to elucidate how excellent HER performance is achieved through these synergistic effects. Finally, we employed a machine learning method to corroborate the difference in synergistic mechanisms between M_1_–N_3_–M_2_–N_3_–C and M_1_–N_4_–M_2_–N_4_–C configurations. Our findings uncover the origin of synergistic effects in DACs from a molecular orbital perspective and lay the theoretical foundation for the precise design and optimization of catalysts to enhance structural stability and break linear scaling relationships in various electrochemical applications.

## 2 Results and Discussion

### 2.1 Characterization of *d* Orbitals in M_1_M_2_N_6_

As shown in Figures S4 and S5, the five *d* orbitals of Ni and Pd in the Ni–N_4_–C and Pd–N_4_–C catalysts are identified as nondegenerate *d*_*x* ^2^–*y* ^2^_, *d*_*z* ^2^_, *d*_*xy*_, *d*_*xz*_, *d*_*yz*_ orbitals (Figure 1b). These orbitals are evident as sharp peaks in the projected density of states (PDOS) of M sites. The introduction of the M_2_ site facilitates synergistic interactions between the *d* orbitals of M_1_ and M_2_. In homonuclear configurations, such as Ni–N_3_–Ni–N_3_–C, Ni–N_4_–Ni–N_4_–C, Pd–N_3_–Pd–N_3_–C, and Pd–N_4_–Pd–N_4_–C, various combinations of bonding and antibonding molecular orbitals are observed (Figure 1c,d). These combinations include *d*_*x* ^2^–*y* ^2^_-*d*_*x* ^2^–*y* ^2^_^*^, *d*_*x* ^2^–*y* ^2^_-*d*_*x* ^2^–*y* ^2^_, *d*_*z* ^2^_-*d*_*z* ^2^_^*^, *d*_*z* ^2^_-*d*_*z* ^2^_, *d*_*xy*_-*d*_xy_^*^, *d*_*xy*_-*d*_*xy*_, *d*_*xz*_-*d*_xz_^*^, *d*_*xz*_-*d*_*xz*_, *d*_*yz*_-*d*_yz_^*^, *d*_*yz*_-*d*_*yz*_ molecular orbitals (* denotes opposite algebraic signs (±) for orbital lobes occupying identical spatial regions as shown in Figure S6). In heteronuclear configurations, like Ni–N_3_–Pd–N_3_–C and Ni–N_4_–Pd–N_4_–C, similar combinations are also observed (Figure S7).^26^ However, in these cases, one of the M_1_ or M_2_ orbitals predominantly influences each molecular orbital, highlighting the auxiliary role of the other metal site. Meanwhile, the orbital interactions within early- (V), mid- (Fe), late- (Cu), and 5*d*- (Pt) transition metals were also investigated. As shown in Figures S8–S17, consistent synergistic effects in the form of *d*-d* combinations are observed. Additionally, the influences of spin–orbital coupling (SOC)^27^ in heavy elements such as Pt, Ir, and Os, as well as the application of the DFT+U correction method,^28^ were examined. These analyses reveal negligible changes in the orbital combination features (Figures S18–S21). This computational insensitivity underscores the robustness of our orbital-based synergistic description across various effects, making it reliable for further exploration. Subsequently, the variation in these synergistic effects across different coordination environments and their differential impact on structural stability, adsorption behavior, and catalytic performance are subsequently discussed based on these molecular orbitals.

### 2.2 Thermodynamic Stability of M_1_M_2_N_6_

We first explored the thermodynamic stability of two distinct M_1_M_2_N_6_ atomic configurations, each involving 26 transition metals (Figure S3): M_1_–N_3_–M_2_–N_3_–C and M_1_–N_4_–M_2_–N_4_–C, with initial intermetallic distances set at 2.47 Å (Figure 1a and Figure S2). To gain molecular orbital-level insights into the synergistic interactions within these configurations, we utilized the corresponding SACs as references. Formation energy (*E*_f_) served as the primary criterion for evaluating thermodynamic stability, defined as

$$E_{f}=\left(E_{M_{1}M_{2}/\mathrm{NC}}-E_{\mathrm{NC}}-E_{M_{1}}-E_{M_{2}}\right)/2$$ 1

where *E*_M_1_M_2_/NC_ represents the total energy of the M_1_M_2_N_6_ system, *E*_NC_ is the total energy of the N_6_C framework, and *E*_M_1__ and *E*_M_2__ denote the average energies of metal atoms in their most stable bulk phases. The calculated *E*_f_ values for both M_1_–N_4_–M_2_–N_4_–C and M–N_4_–C configurations are negative, indicating good thermodynamic stability against metal aggregation. However, some *E*_f_ values for M_1_–N_3_–M_2_–N_3_–C are slightly positive, suggesting a weaker thermodynamic stability. All the results are provided in Tables S1–S3. To further elucidate the discrepancy in structural stability, we calculated α*E*_f_ for comparison, setting α to 0.75 for both the M_1_–N_4_–M_2_–N_4_–C and M–N_4_–C configurations, and to 1 for the M_1_–N_3_–M_2_–N_3_–C configuration (Figure 2a). The results indicate that the average strength of the M_1_/M_2_–N bond in the M_1_–N_3_–M_2_–N_3_–C configuration is weaker than that in the M_1_–N_4_–M_2_–N_4_–C configuration, suggesting that different mechanisms contribute to their thermodynamic stability.

**Figure 2:**
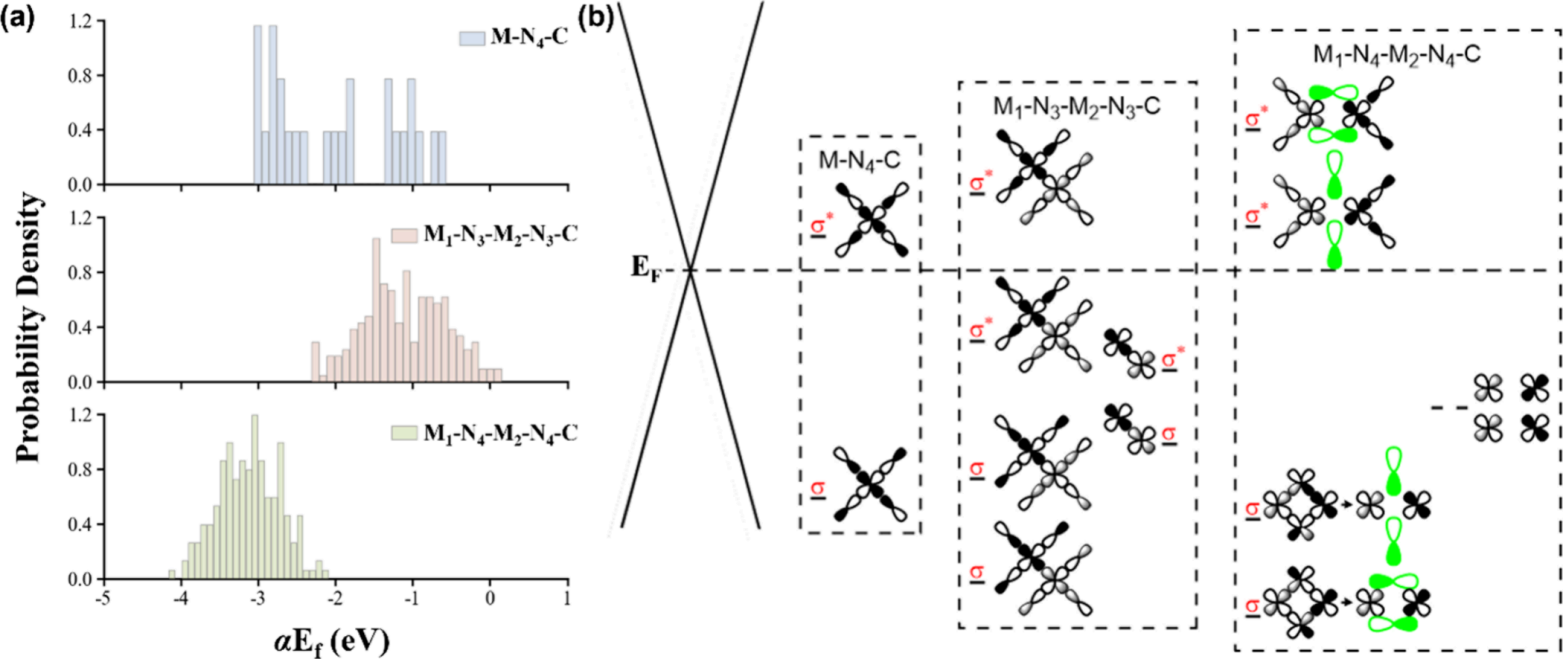
(a) Probability density of α*E*_f_ for M–N_4_–C (α = 0.75), M_1_–N_3_–M_2_–N_3_–C (α = 1.0), and M_1_–N_4_–M_2_–N_4_–C (α = 0.75). (b) Schematic illustration of the orbital hybridization between M_1_, M_2_, and the coordinated N atoms contributing to the structural stability of homonuclear M_1_M_2_N_6_ systems.

To elucidate this disparity, we examined homonuclear DACs, specifically Ni_1_–N_3_–Ni_2_–N_3_–C and Ni_1_–N_4_–Ni_2_–N_4_–C. The α*E*_f_ value for Ni SAC is −2.99 eV, while those for Ni_1_–N_3_–Ni_2_–N_3_–C and Ni_1_–N_4_–Ni_2_–N_4_–C are −1.88 and −3.16 eV, respectively. We employed the integrated crystal orbital Hamiltonian population (ICOHP) to quantify interactions between Ni and the coordinated N atoms, as ICOHP magnitude provides insight into bonding strength, with more negative values indicating stronger bonding. The relationship described by the equation:^29^

$$\Delta E=\frac{{\left|H_{ij}\right|}^{2}}{E_{i}^{0}-E_{j}^{0}}$$ 2

where *H*_*ij*_ is the deviation of resonance integrals from zero under perturbation between orbitals *i* and *j*, guides our analysis. This deviation is influenced by orbital symmetry and overlap, with smaller energy differences between orbitals resulting in a larger Δ*E*, which can upshift antibonding state positions. Therefore, ICOHP not only quantifies bonding strength but also provides information about orbital symmetry and energy level differences, critical for understanding how structural changes affect bonding characteristics.

To further elucidate the variation in Ni–N bond strength as the coordination environment of the Ni site changes, we integrated projected COHP (pCOHP), PDOS, spin-down wave functions, and deformation charge density into our analysis. As shown in Figure 3, the average ICOHP of Ni–N in Ni–N_4_–C is −1.83 eV for both spin-up and spin-down interactions (Figure 3a). In contrast, for Ni_1_–N_3_–Ni_2_–N_3_–C, the average ICOHP for both spin-up and spin-down interactions is −1.68 eV (Figure 3b). For Ni_1_–N_4_–Ni_2_–N_4_–C, the average ICOHP for both spin-up and spin-down interactions is −1.88 eV, with enhanced interactions between Ni and bridge N atoms (Ni–N_bri_) indicated by an ICOHP of −2.00 eV (Figure 3c), while other Ni–N interactions remain largely unchanged compared to those in Ni–N_4_–C. A detailed analysis of the −pCOHP of Ni–N bond in Ni–N_4_–C reveals clear unoccupied antibonding orbitals, corresponding to the *d*_*xy*_ orbital at 1.09 eV in PDOS (Figure 3d). In the cases of Ni_1_–N_3_–Ni_2_–N_3_–C and Ni_1_–N_4_–Ni_2_–N_4_–C, *d*_x^2^–y^2^_ orbitals play a crucial role in Ni–N bonding. The −pCOHP curves of Ni–N bonds in Ni_1_–N_3_–Ni_2_–N_3_–C indicate that one of the two *d*_x^2^–y^2^_ orbitals is positioned below the Fermi level at −0.38 eV, while the other is shifted upward to 1.41 eV (Figure 3d). This suggests a weakening of the Ni–N bond compared to pristine Ni–N_4_–C. Conversely, in Ni_1_–N_4_–Ni_2_–N_4_–C, both *d*_x^2^–y^2^_ orbitals above the Fermi level are moved to higher energy levels at 1.60 and 2.10 eV (Figure 3d), respectively, corresponding to the enhanced thermodynamic stability observed in this structure.

**Figure 3:**
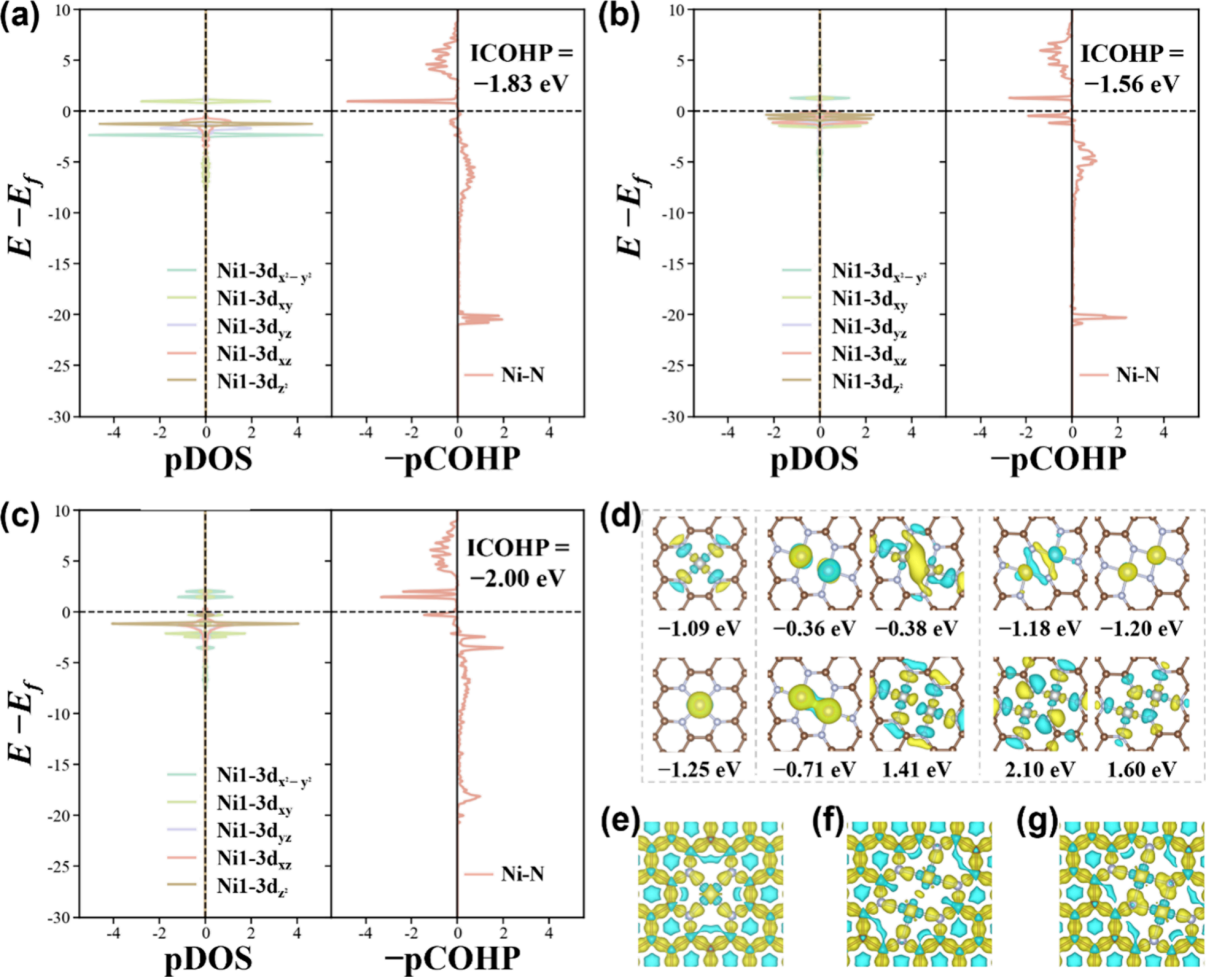
PDOS of Ni_1_ 3*d* orbitals and pCOHP of the Ni–N pair for (a) Ni–N_4_–C, (b) Ni–N_3_–Ni–N_3_–C, and (c) Ni–N_4_–Ni–N_4_–C. (d) From left to right, spin-down wave functions and their corresponding energies relative to the Fermi level for Ni *d*_*xy*_ and *d*_*z* ^2^_ orbitals in Ni–N_4_–C, and *d*_*x* ^2^–*y* ^2^_-*d*_*x* ^2^–*y* ^2^_, *d*_*x* ^2^–*y* ^2^_-*d*_*x* ^2^–*y* ^2^_^*^, *d*_*z* ^2^_-*d*_*z* ^2^_, and *d*_*z* ^2^_-*d*_*z* ^2^_^*^ orbitals of Ni_1_ site in Ni–N_3_–Ni–N_3_–C, and Ni–N_4_–Ni–N_4_–C. Deformation charge density of (e) Ni–N_4_–C, (f) Ni–N_3_–Ni–N_3_–C, and (g) Ni–N_4_–Ni–N_4_–C.

To further elucidate the physical mechanism responsible for the differences in Ni–N bond strengths between Ni_1_–N_3_–Ni_2_–N_3_–C and Ni_1_–N_4_–Ni_2_–N_4_–C, we propose a mechanism based on the analysis of spin-down wave functions of Ni *d*_*x* ^2^–*y* ^2^_ orbitals (Figure 3d). As illustrated in Figure 2b and Figure S22, the *d*_x^2^–y^2^_ atomic orbitals at the Ni_1_ and Ni_2_ sites combine to form bonding *d*_*x* ^2^–*y* ^2^_-*d*_*x* ^2^–*y* ^2^_ and antibonding *d*_*x* ^2^–*y* ^2^_-*d*_*x* ^2^–*y* ^2^_^*^ molecular orbitals. Similar to SACs (Figure 3e), these molecular orbitals in both Ni_1_–N_3_–Ni_2_–N_3_–C and Ni_1_–N_4_–Ni_2_–N_4_–C hybridize with nitrogen 2*p* orbitals, contributing to the thermodynamic stability of DACs. However, in Ni_1_–N_3_–Ni_2_–N_3_–C, a strong head-to-head interaction (Figure 3f) between the *d*_x^2^–y^2^_ orbitals at Ni_1_ and Ni_2_ sites causes deviations in molecular orbital energies, leading to the occupation of an unoccupied (*d*_x^2^–y^2^_-*d*_x^2^–y^2^_)-p* orbital. This results in weakened Ni_1_/Ni_2_–N bonds. Conversely, in Ni_1_–N_4_–Ni_2_–N_4_–C, negligible direct interaction between *d*_x^2^–y^2^_ orbitals (Figure 3g) leads to the degeneracy of bonding and antibonding molecular orbitals. Both (*d*_x^2^–y^2^_-*d*_x^2^–y^2^_)-p* and (*d*_x^2^–y^2^_-*d*_x^2^–y^2^_^*^)-p* molecular orbitals remain above the Fermi level, thereby maintaining Ni–N bond strength. Additionally, in Ni_1_–N_4_–Ni_2_–N_4_–C, the *p*_x_ and *p*_y_ orbitals of bridging nitrogen (N_bri_) atoms (Figure S23), induced by the combinations of Ni_1_-*d*_x^2^–y^2^_ and Ni_2_-*d*_x^2^–y^2^_ orbitals, hybridize into a larger *p* orbital (Figure 3d,g). This hybridization indicates that more electrons from the *p* orbitals participate in interactions with *d*_*x* ^2^–*y* ^2^_-*d*_*x* ^2^–*y* ^2^_ and *d*_*x* ^2^–*y* ^2^_-*d*_*x* ^2^–*y* ^2^_^*^ molecular orbitals, thereby strengthening the Ni_1_/Ni_2_–N_bri_ bonds. Herein, we adopt the unoccupied state population of the *d*_x^2^–y^2^_ orbital (*e*_unocc_) as a descriptor to characterize the Ni_1_–Ni_2_ interaction and evaluate the corresponding Ni–N bonding strength. Direct head-to-head orbital coupling generates partially filled *d*_x^2^–y^2^_-*d*_x^2^–y^2^_ bonding and *d*_x^2^–y^2^_-*d*_x^2^–y^2^_^*^ antibonding orbital combinations, resulting in a lower *e*_unocc_ value. In contrast, indirect orbital interactions effectively increase the degree of unoccupied *d*_x^2^–y^2^_ states, leading to a higher *e*_unocc_ value. As shown in Tables S4 and S5, the *e*_unocc_ of Ni_1_ in Ni_1_–N_3_–Ni_2_–N_3_–C is 0.67 while that in Ni_1_–N_4_–Ni_2_–N_4_–C is higher (0.87), indicating a stronger average Ni–N bonding strength. Overall, the head-to-head interaction between Ni_1_-*d*_x^2^–y^2^_ and Ni_2_-*d*_x^2^–y^2^_ in Ni_1_–N_3_–Ni_2_–N_3_–C determines the energy levels of *d*_x^2^–y^2^_-*d*_x^2^–y^2^_ and *d*_x^2^–y^2^_-*d*_x^2^–y^2^_^*^ states, while in Ni_1_–N_4_–Ni_2_–N_4_–C, these energy levels are instead influenced by N_bri_-mediated *d*_x^2^–y^2^_ interactions. Understanding these two different synergistic mechanisms, one weakening and the other strengthening thermodynamic stability, is crucial for elucidating the structural behavior of M_1_M_2_N_6_ configurations.

In addition to Ni, we also investigated the difference in synergistic cooperation toward Pd–N bonding in Pd–Pd DACs compared to Pd SAC (Figure S24). For both spin-up and spin-down interactions, the dominant contribution to Pd–N bonding arises from the *d*_*xy*_ and *p* orbital interactions, with the antibonding *d*_*xy*_-p* orbital positioned at 2.32 eV. Consequently, the spin-down ICOHP of the Pd–N bond and α*E*_f_ of Pd–N_4_–C are −2.0 and −2.45 eV, respectively. In the Pd_1_–N_3_–Pd_2_–N_3_–C structure, a σ bond forms between Pd_1_-*d*_x^2^–y^2^_ and Pd_2_-*d*_x^2^–y^2^_, creating bonding *d*_x^2^–y^2^_-*d*_x^2^–y^2^_ and antibonding *d*_x^2^–y^2^_-*d*_x^2^–y^2^_^*^ molecular orbitals. Upon interacting with N 2*p* orbitals, one antibonding (*d*_x^2^–y^2^_-*d*_x^2^–y^2^_)-p* orbital is occupied below the Fermi level at −0.23 eV, while the other antibonding (*d*_x^2^–y^2^_-*d*_x^2^–y^2^_^*^)-p* orbital is shifted upward to 2.49 eV. The corresponding *e*_unocc_ value of Pd is 0.61. Consequently, the ICOHP for both spin-up and spin-down Pd–N bonds is −1.61 eV, while the α*E*_f_ of Pd_1_–N_3_–Pd_2_–N_3_–C is −1.30 eV. For Pd_1_–N_4_–Pd_2_–N_4_–C, interactions mediated by N_bri_ rather than head-to-head interactions between Pd_1_-*d*_x^2^–y^2^_ and Pd_2_-*d*_x^2^–y^2^_ lead to the degeneracy of bonding *d*_x^2^–y^2^_-*d*_x^2^–y^2^_ and antibonding *d*_x^2^–y^2^_-*d*_x^2^–y^2^_^*^ molecular orbitals prior to interaction with N 2*p* orbitals. In contrast to Pd_1_–N_3_–Pd_2_–N_3_–C, both antibonding (*d*_x^2^–y^2^_-*d*_x^2^–y^2^_)-p* and (*d*_x^2^–y^2^_-*d*_x^2^–y^2^_^*^)-p* orbitals remain above the Fermi level and are pushed upward to 2.94 and 3.00 eV, respectively, due to the hybridization of N_bri_ *p*_*x*_ and *p*_*y*_ orbitals. The corresponding *e*_unocc_ value of Pd is 0.83. As a result, for both spin-up and spin-down interactions, the ICOHP of Pd–N bonds remains at the level of −2.0 eV, similar to that in Pd–N_4_–C, while Pd_1_/Pd_2_–N_bri_ bonds exhibit an enhanced ICOHP of −2.13 eV. This enhancement in Pd_1_/Pd_2_–N_bri_ bonds contributes to the increased thermodynamic stability of Pd_1_–N_4_–Pd_2_–N_4_–C, as indicated by the α*E*_f_ value of −4.63 eV.

Additionally, we extended this analysis to heteronuclear DACs to verify the difference in synergistic mechanisms that modulates their structural stability between heteronuclear M_1_–N_3_–M_2_–N_3_–C (Figure S25) and M_1_–N_4_–M_2_–N_4_–C (Figure S26). Ni_1_–N_3_–Pd_2_–N_3_–C and Ni_1_–N_4_–Pd_2_–N_4_–C were selected as examples. As depicted in Figure S7, the strong in-plane interactions between Ni_1_-*d*_x^2^–y^2^_ and Pd_2_-*d*_x^2^–y^2^_ persist in Ni_1_–N_3_–Pd_2_–N_3_–C, resulting in a (*d*_x^2^–y^2^_-*d*_x^2^–y^2^_)-p* orbital located below the Fermi level at −0.34 eV and a (*d*_x^2^–y^2^_-*d*_x^2^–y^2^_^*^)-p* orbital above the Fermi level at 1.84 eV. The *e*_unocc_ values of Ni_1_ and Pd_2_ are 0.61 and 0.65, respectively. This strong in-plane interaction in Ni_1_–N_3_–Pd_2_–N_3_–C weakens the Ni_1_/Pd_2_–N bonds and yields an average ICOHP of −1.67 eV for Ni–N and −1.77 eV for Pd–N bonds, resulting in an α*E*_f_ of −1.62 eV. In contrast, direct interactions between the Ni_1_-*d*_x^2^–y^2^_ and Pd_2_-*d*_x^2^–y^2^_ orbitals are negligible in Ni_1_–N_4_–Pd_2_–N_4_–C. Unlike in homonuclear DACs, one *d*_x^2^–y^2^_-*d*_x^2^–y^2^_ orbital is predominantly contributed by Pd_2_-*d*_x^2^–y^2^_ and is located at 3.16 eV, while the other *d*_x^2^–y^2^_-*d*_x^2^–y^2^_^*^ orbital is mainly composed of Ni_1_-*d*_x^2^–y^2^_ and is situated at 1.78 eV. The *e*_unocc_ values of Ni_1_ and Pd_2_ are increased to 0.86 and 0.83, respectively. This configuration enhances the thermodynamic stability of Ni_1_–N_4_–Pd_2_–N_4_–C, as indicated by an α*E*_f_ of −2.76 eV. As illustrated in Figures S28 and S30a, the in-plane interactions in heteronuclear DACs extend beyond M_1_-*d*_x^2^–y^2^_ and M_2_-*d*_x^2^–y^2^_ orbitals, involving *d*_*xy*_ orbitals as well. The occupation of antibonding (*d*_*xy*_-*d*_x^2^–y^2^_)-p* orbitals can also weaken M_1_/M_2_–N bonds. In M_1_–N_4_–M_2_–N_4_–C, the in-plane interactions contributing to their thermodynamic stability remain primarily N-mediated M_1_-*d*_x^2^–y^2^_ and M_2_-*d*_x^2^–y^2^_ interactions, as evidenced by wave function and deformation charge density analyses (Figures S29 and S30b). These interactions help maintain or strengthen M_1_/M_2_–N bonds relative to corresponding SACs.

For early transition metals with low valence electron numbers (e.g., Ti and V), the occupied *d*_x^2^–y^2^_ orbital occupation (*e*_occ_) was utilized as the characteristic descriptor to correlate with ICOHP values and formation energies (Figure S22). Direct M_1_–M_2_ interactions result in partially occupied bonding *d*_x^2^–y^2^_-*d*_x^2^–y^2^_ and antibonding *d*_x^2^–y^2^_-*d*_x^2^–y^2^_^*^ combinations, leading to a lower *e*_occ_ value in Ti/V–N_3_–M_2_–N_3_–C compared to Ti/V–N_4_–M_2_–N_4_–C, where N_bri_-mediated M_1_–M_2_ interactions lead to a higher *e*_occ_. Taking V_1_–N_3_–V_2_–N_3_–C and V_1_–N_4_–V_2_–N_4_–C as examples, the *e*_occ_ values of V are 0.53 and 0.72, respectively. The higher occupied *d*_x^2^–y^2^_ orbital contributes to stronger V–N bonding strength, as indicated by the ICOHP of −2.62 eV for the V–N pair in V_1_–N_4_–V_2_–N_4_–C, compared to the weaker V–N bond in V_1_–N_3_–V_2_–N_3_–C, which has an ICOHP of −2.16 eV due to the lower *e*_occ_ value of 0.53. The corresponding α*E*_f_ values are −1.44 and −3.59 eV, indicating greater structural stability of V_1_–N_4_–V_2_–N_4_–C compared to V_1_–N_3_–V_2_–N_3_–C. A comprehensive understanding of these mechanisms, both in homonuclear and heteronuclear DACs, provides valuable insights into the structural behavior of M_1_M_2_N_6_ configurations, which is essential for the rational design and optimization of catalytic systems, particularly concerning structural stability.

### 2.3 Hydrogen Adsorption on M_1_M_2_N_6_

Building on the aforementioned analysis, we anticipate that the adsorption behavior on M_1_M_2_N_6_ configurations will be significantly influenced by the synergistic interactions between discrete *d* orbitals of M_1_ and M_2_ atoms, differing from the coordination environment observed in M_1_–N_3_–M_2_–N_3_–C and M_1_–N_4_–M_2_–N_4_–C. To elucidate this, we initiated a study on hydrogen adsorption behavior and how the synergistic cooperation between frontier orbitals of M_1_ and M_2_ sites modulates hydrogen adsorption energies (Δ*G*_H*_). For DACs, two distinct adsorption sites for hydrogen (H) are considered: the site corresponding to the more stable configuration is denoted as M_1_, while the alternative metal site is designated as M_2_. Detailed Δ*G*_H*_ values of both SACs and DACs are presented in Tables S4–S6. The computed Δ*G*_H*_ values for SACs range from −1.21 eV for Hf–N_4_–C to 2.12 eV for Au–N_4_–C (Figure 4a). Notably, none of the SACs, including Co–N_4_–C, exhibit adsorption energy close to 0 eV (|Δ*G*_H*_| ≤ 0.08 eV). With the introduction of M_2_ sites, the computed Δ*G*_H*_ values for M_1_–N_3_–M_2_–N_3_–C range from −1.51 to 0.76 eV (Figure 4b), while those for M_1_–N_4_–M_2_–N_4_–C range from −1.13 to 2.41 eV (Figure 4c). Specifically, compared to M_1_–N_4_–M_2_–N_4_–C, the Δ*G*_H*_ values on M_1_–N_3_–M_2_–N_3_–C exhibit a clear leftward trend, indicating a more negative adsorption energy. This trend is particularly pronounced for catalysts with positive Δ*G*_H*_ values, suggesting that the synergistic mechanisms modulating hydrogen adsorption in M_1_–N_3_–M_2_–N_3_–C differ from those in M_1_–N_4_–M_2_–N_4_–C. To quantitatively describe catalytic activity, the relationship between Δ*G*_H*_ and exchange current (*i*_0_) is explored. The exchange current *i*_0_ can be calculated using the following formula:

$$i_{0}=-ek_{0}\frac{1}{\exp\left(\left|\Delta G_{H*}\right|/k_{B}T\right)}$$ 3

where *e* is the elementary charge, *k*_B_ is the Boltzmann constant, *T* is the temperature, and *k*_0_ is the reaction rate constant, which is set to 1.^30^ In contrast to SACs, some DACs emerge as optimal catalysts located near the peak of the volcano plot, exhibiting superior catalytic activity compared to the benchmark catalyst Pt (111).^31^ Of particular interest are the homonuclear Ni–N_3_–Ni–N_3_–C (Δ*G*_H*_ = 0.05 eV) and the heteronuclear Ni–N_4_–Os–N_4_–C (Δ*G*_H*_ = −0.04 eV) and Co–N_3_–Cu–N_3_–C (Δ*G*_H*_ = 0.02 eV) catalysts, along with their corresponding Ni–N_4_–Ni–N_4_–C, Ni–N_3_–Os–N_3_–C, Co–N_4_–Cu–N_4_–C, and SACs. These systems are studied to uncover the physical origin of synergistic mechanisms that lead to excellent HER performance.

**Figure 4:**
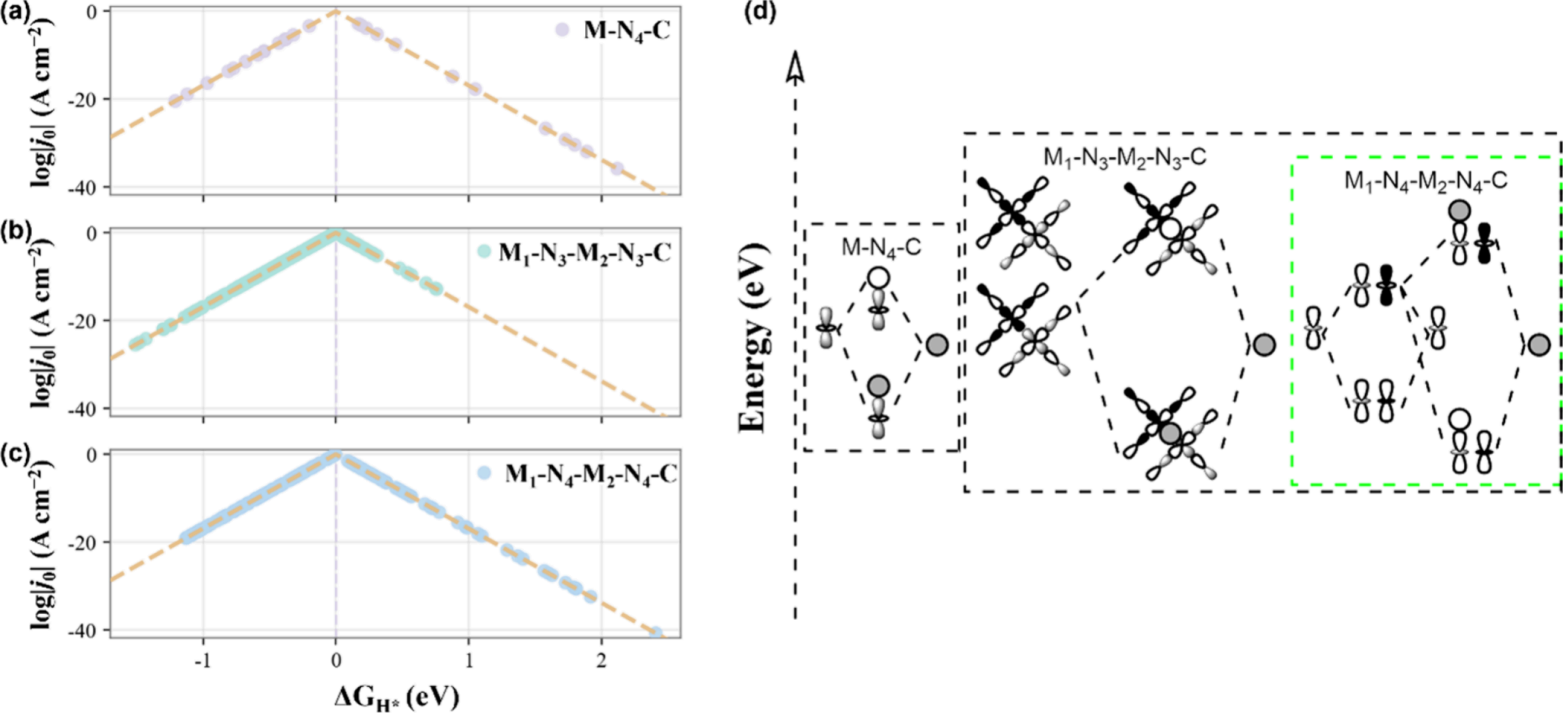
Exchange current density for H adsorption on (a) M–N_4_–C, (b) M_1_–N_3_–M_2_–N_3_–C, and (c) M_1_–N_4_–M_2_–N_4_–C. (d) Schematic illustration of the orbital hybridization between M_1_ and H atoms contributing to hydrogen adsorption on homonuclear M_1_M_2_N_6_ systems.

As shown in Figure 3d, the Ni-*d*_z^2^_ orbital in Ni SAC is positioned at −1.25 eV prior to hydrogen adsorption. The unoccupied antibonding *d*_z^2^_-*s* * orbital facilitates hydrogen adsorption and is located at 0.06 eV (Figure 5a). This results in an ICOHP of −1.32 eV for Ni–H and a Δ*G*_H*_ value of 1.72 eV (Figure 5f), which is clearly unfavorable for HER performance. Incorporating a Ni_2_ site into Ni–N_4_–Ni–N_4_–C induces weak N-mediated interactions between Ni_1_-*d*_z^2^_ and Ni_2_-*d*_z^2^_ orbitals, creating a bonding *d*_z^2^_-*d*_z^2^_ orbital at −1.20 eV and an antibonding *d*_z^2^_-*d*_z^2^_^*^ orbital at −1.18 eV (Figure S31c). Consequently, the Δ*G*_H*_ and ICOHP values are mildly adjusted to 1.42 and −1.37 eV with antibonding (*d*_z^2^_-*d*_z^2^_^*^)-s* orbital positioned at 0.59 eV (Figure 5b), which remains unfavorable for HER performance. In contrast, for Ni–N_3_–Ni–N_3_–C, hydrogen adsorption is wildly enhanced, and yields a Δ*G*_H*_ value of 0.05 eV, indicating excellent HER performance. This wild enhancement is attributed to the strong in-plane interactions between the discrete *d*_x^2^–y^2^_ orbitals of Ni_1_ and Ni_2_ atoms. Unlike Ni–N_4_–Ni–N_4_–C, which exhibits end-on hydrogen adsorption, hydrogen is bridge-adsorbed across the Ni–Ni dual sites in Ni–N_3_–Ni–N_3_–C (Figure 5e), leading to a more favorable adsorption energy. The mechanism underlying this change in adsorption pattern is further elucidated through PDOS, wave function, and deformation charge density analyses, which reveal that an antibonding (*d*_x^2^–y^2^_-*d*_x^2^–y^2^_)-s* orbital (Figure 5b), rather than an antibonding (*d*_*z* ^2^_-*d*_*z* ^2^_^*^)-s* orbital, is positioned above the Fermi level at 1.16 eV. This finding clarifies the physical mechanism responsible for wildly enhanced hydrogen adsorption on Ni_1_–N_3_–Ni_2_–N_3_–C (Figure 5f). Specifically, the head-to-head interaction between Ni_1_-*d*_x^2^–y^2^_ and Ni_2_-*d*_x^2^–y^2^_ orbitals results in the bonding *d*_x^2^–y^2^_-*d*_x^2^–y^2^_ orbital being positioned below the Fermi level at −0.38 eV. Meanwhile, the side interaction between Ni_1_-*d*_z^2^_ and Ni_2_-*d*_z^2^_ orbitals positions an antibonding *d*_z^2^_-*d*_z^2^_^*^ orbital near the Fermi level at −0.36 eV (Figure 3d). The orbital symmetry in Ni_1_–N_3_–Ni_2_–N_3_–C facilitates bridge adsorption through the bonding *d*_x^2^–y^2^_-*d*_x^2^–y^2^_ orbital, while end-on adsorption is limited by the antibonding *d*_z^2^_-*d*_z^2^_^*^ orbital. To minimize energy, bridge adsorption involving two *d*_x^2^–y^2^_ orbitals (ICOHP = −2.10 eV for two Ni–H pairs) is favored on Ni_1_–N_3_–Ni_2_–N_3_–C, as opposed to end-on adsorption involving a single *d*_z^2^_ orbital. Interestingly, bridge-site hydrogen adsorption induces orbital rearrangement: partial *d*_x^2^–y^2^_-*d*_x^2^–y^2^_ states shift upward across the Fermi level and reorganize into (*d*_x^2^–y^2^_-*d*_x^2^–y^2^_)-s* molecular orbitals (Figure S32). The reinforced Ni–N bond is validated by the ICOHP values, which evolve from −1.56 to −1.74 eV. We further quantified the occupation of the *d*_x^2^–y^2^_ orbital localized at the Ni_1_ site over the energy range of *E*_F_–2 to *E*_F_, where the integrated occupation number decreases from 0.89 to 0.40. This strengthening of the Ni–N bond stems from suppressed metallic coupling between Ni_1_ and Ni_2_, as reflected by the corresponding ICOHP value increasing from −0.98 to −0.73 eV. In contrast to the wild modulation of hydrogen adsorption achieved through the bridge configuration in Ni_1_–N_3_–Ni_2_–N_3_–C, hydrogen adsorption on Ni_1_–N_4_–Ni_2_–N_4_–C is constrained by mild modulation with an end-on configuration. This limitation arises because only N-mediated antibonding (*d*-*d*/d*)-p* orbitals are located near the Fermi level (Figure S4c), restricting the adsorption pattern and resulting in only a mild improvement in Δ*G*_H*_.

**Figure 5:**
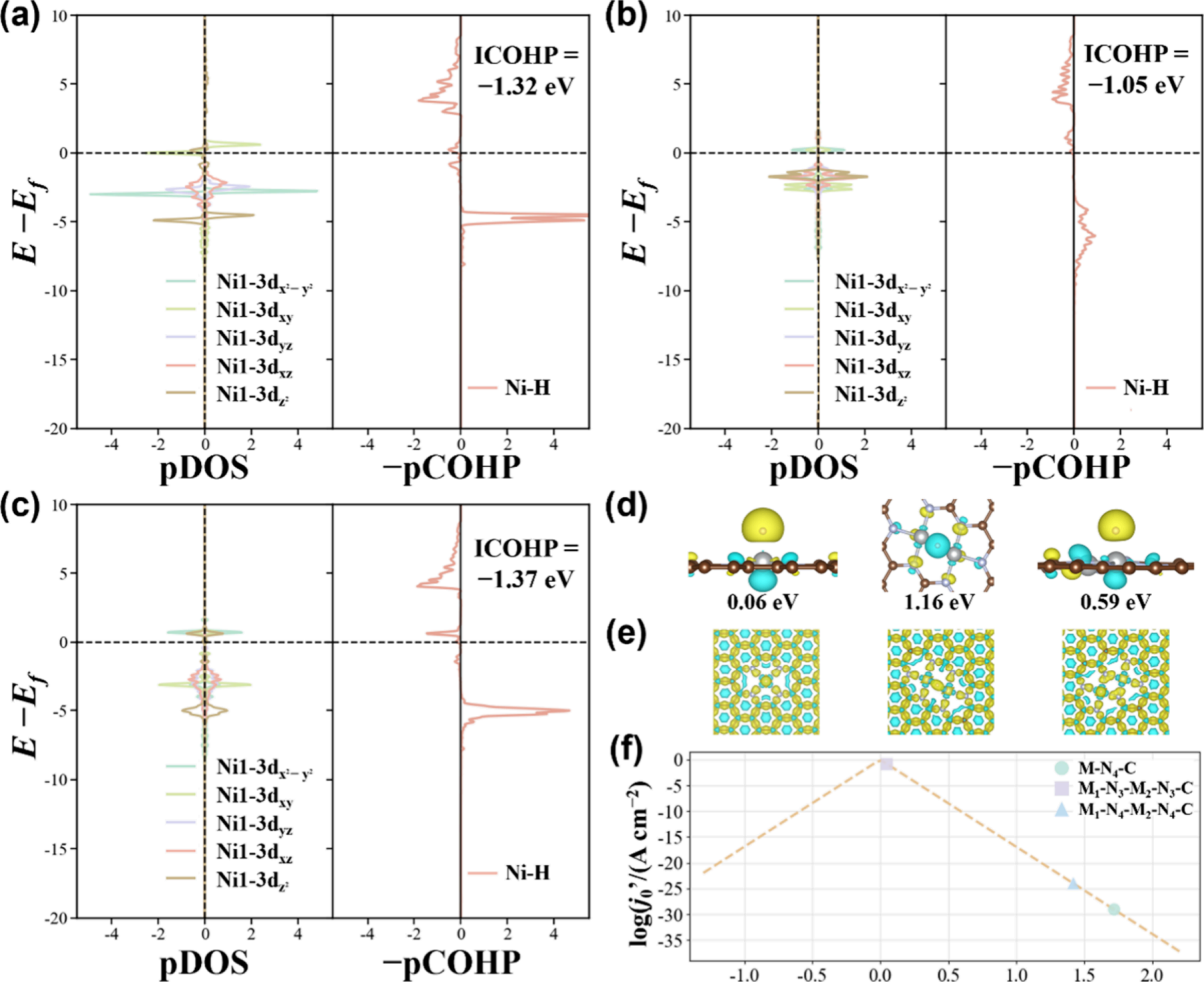
PDOS of Ni_1_ 3*d* orbitals and pCOHP of the Ni–H pair for (a) Ni–N_4_–C, (b) Ni–N_3_–Ni–N_3_–C, and (c) Ni–N_4_–Ni–N_4_–C. (d) Spin-down wave functions and their corresponding energies relative to the Fermi level for antibonding *d*_z^2^_-s*, (*d*_x^2^–y^2^_-*d*_x^2^–y^2^_)-s* and (*d*_z^2^_-*d*_z^2^_^*^)-s* orbitals. (e) Deformation charge density. (f) Exchange current density for H adsorption.

A similar transition from mild modulation via end-on hydrogen adsorption to wild modulation via bridge hydrogen adsorption, facilitated by orbital symmetry, is also observed in heteronuclear M_1_–N_3_–M_2_–N_3_–C. For instance, in Co–N_3_–Cu–N_3_–C, in-plane interactions among *d* orbitals of Cu and Co sites contribute to the formation of bonding *d*_*xy*_-*d*_x^2^–y^2^_ orbitals positioned below the Fermi level at −0.92 and −1.52 eV, respectively (Figure S28). Meanwhile, the antibonding *d*_z^2^_-*d*_z^2^_^*^ orbital, primarily composed of Co_1_-*d*_z^2^_ states, is positioned at −0.16 eV. However, bridge adsorption is favored over end-on adsorption for hydrogen, with the corresponding antibonding orbital located at 2.30 eV (Figure S34a). Consequently, hydrogen adsorption is widely enhanced, resulting in a Δ*G*_H*_ value of 0.02 eV, indicating superior HER performance compared to both Co–N_4_–C (0.19 eV) and Cu–N_4_–C (1.80 eV). In Co–N_4_–Cu–N_4_–C, hydrogen adsorption is constrained by orbital symmetry, with hydrogen atom being end-on adsorbed by Co_1_-*d*_z^2^_ in the *d*_z^2^_-*d*_z^2^_^*^ molecular orbital located at −0.49 eV (Figure S29). This configuration results in an antibonding (*d*_z^2^_-*d*_z^2^_^*^)-s* molecular orbital at 1.76 eV (Figure S34b) and a Δ*G*_H*_ value of 0.48 eV, indicating unfavorable HER performance.

In addition to bridge adsorption, end-on hydrogen adsorption on both homonuclear and heteronuclear M_1_–N_3_–M_2_–N_3_–C catalyst can also be effective when hydrogen adsorption on M_1_–N_4_–C is strong (TableS6 and Figure S35). For example, in Ni–N_3_–Os–N_3_–C, the Δ*G*_H*_ values for corresponding SACs are −0.69 eV for Os–N_4_–C and 1.72 eV for Ni–N_4_–C. Specifically, the Os-*d*_z^2^_ states within *d*_z^2^_-*d*_z^2^_^*^ molecular orbital at −0.13 eV (Figure S30a) facilitate hydrogen adsorption through an end-on configuration. The antibonding (*d*_z^2^_-*d*_z^2^_^*^)-s* orbital is located at 2.41 eV (Figure S34c), slightly lower than the *d*_z^2^_-s* orbital at 2.46 eV for Os–N_4_–C (Figure S33d), resulting in a weakened Δ*G*_H*_ value of −0.60 eV, indicating only mild modulation toward HER performance. For Ni–N_4_–Os–N_4_–C, the introduction of Ni_2_ site causes a downward shift in the position of Os-*d*_z^2^_ in *d*_z^2^_-*d*_z^2^_^*^ molecular orbital to −1.00 eV (Figure S30b), leading to reduced hydrogen adsorption with a Δ*G*_H*_ value of −0.04 eV. The corresponding antibonding (*d*_z^2^_-*d*_z^2^_^*^)-s* orbital is positioned at 1.81 eV (Figure S34d), achieving excellent HER performance.

To conclude (Figure 4b), hydrogen adsorption with only an end-on configuration on M_1_–N_4_–M_2_–N_4_–C is mildly modulated by the synergistic operation between N-mediated M_1_ and M_2_ sites. In contrast, for both homonuclear and heteronuclear M_1_–N_3_–M_2_–N_3_–C, when hydrogen adsorption on M_1_ sites is strong, a mild modulation in hydrogen adsorption can be achieved through an end-on configuration. Otherwise, the adsorption pattern shifts to bridge adsorption, facilitated by the involvement of *d*_x^2^–y^2^_ orbitals, leading to a wild modulation in hydrogen adsorption. Consequently, compared to M_1_–N_4_–M_2_–N_4_–C, the Δ*G*_H*_ values of M_1_–N_3_–M_2_–N_3_–C exhibit a clear leftward trend, particularly pronounced for catalysts with positive Δ*G*_H*_ values on the right side of the volcano plot (Figure 4a). This distinct trend in Δ*G*_H*_ values arises from the unique orbital synergy in M_1_M_2_N_6_ configurations, leading to different adsorption behaviors and further catalytic activities. Understanding these differences is crucial for elucidating the structure–property relationship between M_1_–N_3_–M_2_–N_3_–C and M_1_–N_4_–M_2_–N_4_–C.

### 2.4 Machine Learning for M_1_M_2_N_6_

To further verify the difference in synergistic mechanism between M_1_–N_3_–M_2_–N_3_–C and M_1_–N_4_–M_2_–N_4_–C, we employed an extreme gradient boosting (XGBoost) machine learning (ML) model implemented with the scikit-learn package^32^ to analyze the relationship between intrinsic properties and catalytic activity. A comprehensive ML training database was constructed on DFT-calculated features, including the distance between M_1_ and M_2_ (*L*_M_1_–M_2__), the d-band center of transition metal atoms (*ε*_d_), and the charge transfer from metal centers to supports (*Q*_M_). Additional intrinsic properties such as the valence electron number (*N*_M_), d-electron number (θ_*d*_), and Pauling electronegativity (χ_M_) were sourced from the Mendeleev database. The data set comprised 32 entries with hydrogen adsorption energies (Δ*G*_H*_) as the target variable. We utilized a 5-fold cross-validation approach, allocating 80% of the data for training and 20% for testing, which minimizes errors from random data splits and enhances the model’s performance evaluation.

Figures S36–S38 present the collected data distribution, showcasing individual variable distributions, pairwise relationships, and correlation analyses.^33^ Notably, the Pearson correlation coefficients reveal a strong positive correlation between Δ*G*_H*_ and intrinsic properties such as *N*_M_, θ_*d*_, and *Q*_M_. Conversely, a strong negative correlation exists between Δ*G*_H*_ and *Q*_M_, indicating that these features are critical for hydrogen adsorption. The introduction of M_2_ sites appears to weaken the influence of M_1_ properties on Δ*G*_H*_, while nonzero correlations at the M_2_ site suggest that M_2_ plays an auxiliary role in modulating hydrogen adsorption. Despite these insights into the contributions of M_1_ and M_2_, they do not fully elucidate the difference in synergistic mechanisms affecting Δ*G*_H*_ between the two configurations.

Toward the end, the XGBoost model was trained, yielding a consistent trend with DFT-calculated values, as illustrated in Figure 6a,b, which shows a clear linear relationship for both training and testing data sets (*R* ^2^ = 0.98 for M_1_–N_3_–M_2_–N_3_–C, and *R* ^2^ = 0.99 for M_1_–N_4_–M_2_–N_4_–C). The SHapley Additive exPlanations (SHAP) method was employed to evaluate the contribution of features in the XGBoost model. The importance of the top six features is depicted in Figure 6 and Figure S39. For M_1_–N_3_–M_2_–N_3_–C, key features influencing hydrogen adsorption include the covalent radius (*C*_M_1__) and charge transfer *Q*_M_1__ at M_1_, alongside the atomic number (*A*_M_2__), *E*_M_2__, first ionization energy (IE_M_2__), and van der Waals (vdW) radius (*V*_M_2__) at M_2_. In contrast, for M_1_–N_4_–M_2_–N_4_–C, while *Q*_M_1__, *C*_M_1__, metal atom radius (*r*_M_1__), and atomic volume (Av_M_1__) at M_1_ remain significant, only *L*_M_1_–M_2__ and *N*_M_2__ at M_2_ show substantial importance, indicating a limited impact on hydrogen adsorption. These findings align with previous orbital analyses, suggesting that direct M_1_–M_2_ interactions in M_1_–N_3_–M_2_–N_3_–C and indirect N-mediated interactions in M_1_–N_4_–M_2_–N_4_–C contribute to differences in Δ*G*_H*_ modulation.

**Figure 6:**
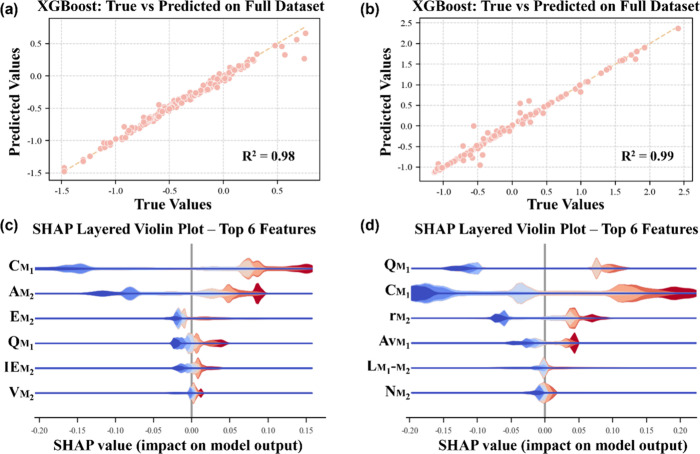
DFT-calculated and ML-predicted Δ*G*_H*_ for (a) M_1_–N_3_–M_2_–N_3_–C and (b) M_1_–N_4_–M_2_–N_4_–C. SHAP summary plots displaying the effects of top six features and their values on the prediction for (c) M_1_–N_3_–M_2_–N_3_–C and (d) M_1_–N_4_–M_2_–N_4_–C. The *y*-axis of each plot lists the features included in the model, sorted from the most (top) to least (bottom) important, and the *x*-axis depicts SHAP value. The color displays whether the feature value is high (red) or low (blue).

## 3 Conclusions

In conclusion, our study employs large-scale DFT calculations to investigate how coordination environment-dependent synergistic cooperation within the isolated atomic orbitals of M_1_ and M_2_ sites in M_1_M_2_N_6_ configurations influences structural stability, adsorption behavior, and catalytic performance. We reveal that the synergistic effects arise from the combination of *d* atomic orbitals into molecular orbitals between M_1_ and M_2_ sites, with significant variations observed from M_1_–N_3_–M_2_–N_3_–C to M_1_–N_4_–M_2_–N_4_–C. In contrast to SACs, head-to-head *d*_x^2^–y^2^_ interactions, rather than N-mediated *d*_x^2^–y^2^_ interactions, shift part of the *d*_x^2^–y^2^_-related molecular orbitals below/above the Fermi level, thereby weakening the structural stability of M_1_–N_3_–M_2_–N_3_–C. Meanwhile, the hybridization of N_bri_ 2*p* orbitals contributes to the enhanced structural stability of M_1_–N_4_–M_2_–N_4_–C compared to SACs. The difference in synergistic effects further influences the adsorption behavior, with hydrogen serving as a model adsorbate. Our results indicate that *d*_x^2^–y^2^_-related molecular orbitals below the Fermi level enable a bridge configuration, resulting in a wild modulation of hydrogen adsorption. In contrast, the end-on configuration, attributed to *d*_z^2^_-related molecular orbitals, leads to a mild modulation of hydrogen adsorption. Machine learning analyses confirm that hydrogen adsorption on M_1_ sites is much less influenced by M_2_ sites in M_1_–N_4_–M_2_–N_4_–C compared to M_1_–N_3_–M_2_–N_3_–C, thereby corroborating the difference in synergistic mechanisms: direct head-to-head interactions for M_1_–N_3_–M_2_–N_3_–C and indirect N-mediated interactions for M_1_–N_4_–M_2_–N_4_–C. This nuanced visualization of the coordination environment-dependent synergistic mechanisms from a molecular orbital perspective in M_1_M_2_N_6_ catalysts will facilitate the precise design and optimization of DACs.

## Supplementary Material



## Notes

Computational methods; construction of M_1_M_2_N_6_; identification of *d* orbitals in M_1_M_2_N_6_; identification of d orbitals and −pCOHP curves on thermodynamic stability of M_1_M_2_N_6_; hydrogen adsorption on M_1_M_2_N_6_; machine learning for M_1_M_2_N_6_; formation energies of M–N_4_–C; formation energies of M_1_–N_3_–M_2_–N_3_–C; formation energies of M_1_–N_4_–M_2_–N_4_–C; structural stability and correlated descriptors for M_1_–N_3_–M_2_–N_3_–C; structural stability and correlated descriptors for M_1_–N_4_–M_2_–N_4_–C; hydrogen adsorption energies of M–N_4_–C; hydrogen adsorption energies of M_1_–N_3_–M_2_–N_3_–C; hydrogen adsorption energies of M_1_–N_4_–M_2_–N_4_–C (PDF)
