# Supplementary material for: Role of Coordination Environment in Synergistic Catalysis: A Molecular Orbital Perspective on M1M2N6 Catalysts
Source: J Am Chem Soc. 2026 Jul 16;148(29):31359–68. doi: 10.1021/jacs.6c08871 (PMC13426260; doi:10.1021/jacs.6c08871)
Supplement: Supplementary file 1 [file ja6c08871_si_001.pdf]

Supporting Information for
The Role of Coordination Environment in Synergistic Catalysis: A Molecular Orbital Perspective on $M_1M_2N_6$ Catalysts

Zhangsheng Shi[+a], Ziyang Wu[+b], Zheng Shu[c], and Xin Wang *[a]

[a]Department of Chemistry, City University of Hong Kong, HongKong SAR 999077, PR China
[b]State Key Laboratory of Advanced Fiber Materials, College of Materials Science and Engineering, Donghua University, Shanghai 201620, China
[c]Department of Chemistry, National University of Singapore, Singapore 117543, Republic of Singapore

S1 **Computational methods** **2**

S2 **Construction of $M_1M_2N_6$** **2**

S3 **Identification of d orbitals in $M_1M_2N_6$** **3**

S4 **Identification of d orbitals and −pCOHP curves on thermodynamic stability of $M_1M_2N_6$** **13**

S5 **Hydrogen adsorption on $M_1M_2N_6$** **16**

S6 **Machine learning for $M_1M_2N_6$** **19**

S7 **Formation energies of M-N_4-C, and $M_1M_2N_6$.** **22**

S8 **Hydrogen adsorption energies on M-N_4-C, and $M_1M_2N_6$.** **31**

*Corresponding author: wang.xin@cityu.edu.hk

S1 Computational methods

All spin-polarized DFT calculations were performed using the Vienna ab initio simulation package (VASP).[1, 2] The Perdew- Burke-Ernzerhof (PBE) functional exchange-correlation functional[3] was employed in conjunction with the projector-augmented wave (PAW)[4] method to handle core-valence interactions. A plane-wave cut-off energy of 500 eV was used, and a graphene supercell with lattice dimensions of 17.09 Å×17.26 Å was adopted. All surface slabs were constructed with a vacuum layer of 20 Å. A 1×1×1 k-point grid was utilized for for reciprocal space sampling, and the energy convergence threshold was set to 10^{-5} eV. Geometry optimizations were performed until force convergence below 0.01 eV/Å. The DFT D3 method[5] was implemented to account for van deer Waals (vdW) interactions. Density of states (DOS) calculations employed a 3×3×1 k-point grid with an energy convergence threshold of 10^{-6} eV. Crystal orbital Hamilton population (COHP) analysis was conducted using the LOBSTER 5.1.1 package to analyze pairwise interactions.[6, 7] Structural visualizations were generated with VESTA,[8] and post-processing of VASP data was performed using the VASPKIT package.[9] The Gibbs free energy change (ΔG) is given by:

$$\Delta G = \Delta E_{\mathrm{DFT}} + \Delta E_{\mathrm{ZPE}} + \int C_p dT - T\Delta S \tag{SE1}$$

where ΔE_{DFT} represents the DFT-calculated electronic energy difference between each step, ΔE_{ZPE} is the zero-point energy correction, $\int C_p dT$ accounts for enthalpy corrections, and $T\Delta S$ incorporates entropy effects.

S2 Construction of $M_1M_2N_6$

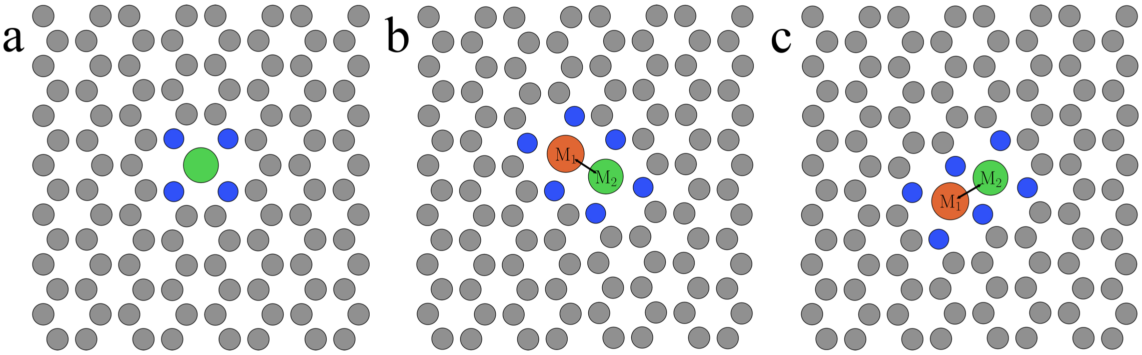

Figure S1: Metal-nitrogen-doped carbon models used in the calculations. (a)M-N_4-C. (b) M_1-N_3-M_2-N_3-C. (c) M_1-N_4-M_2-N_4-C.

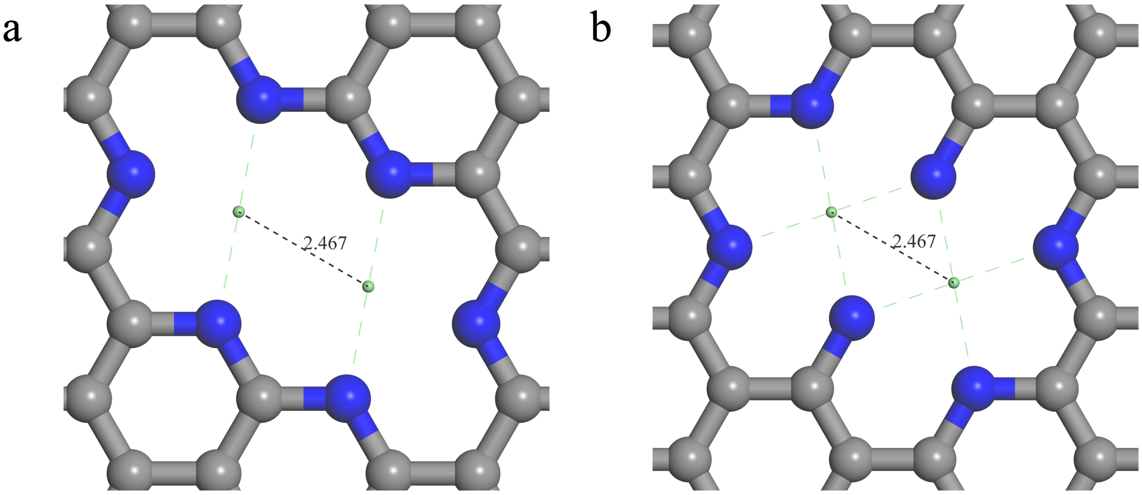

Figure S2: The initial distance between M_1 and M_2 sites is 2.47 Å in (a) M_1-N_3-M_2-N_3-C and (b) M_1-N_4-M_2-N_4-C.

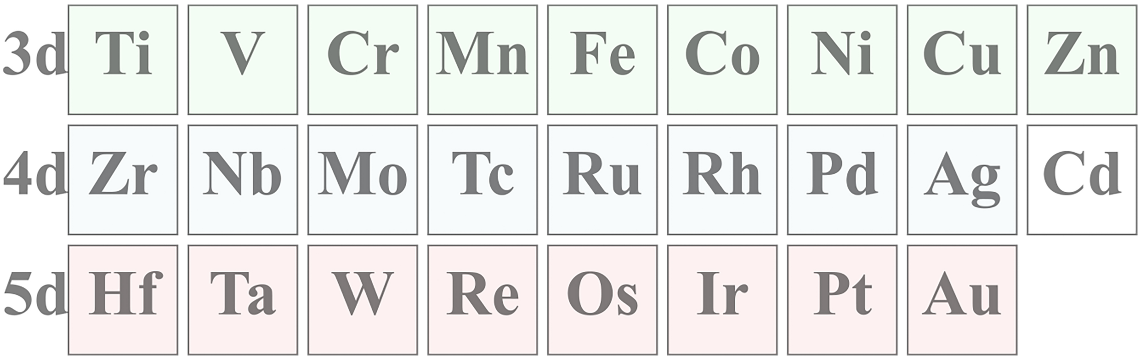

Figure S3: 26 transition metals are adopted as M_1 and M_2 sites in $M_1M_2N_6$.

S3 Identification of d orbitals in $M_1M_2N_6$

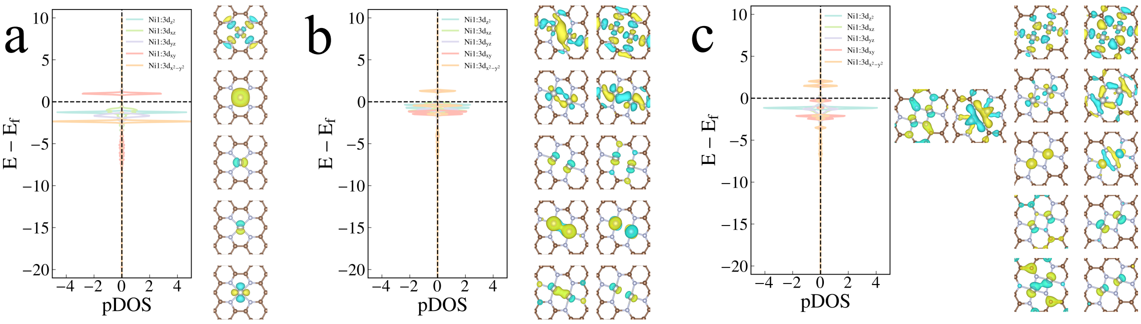

Figure S4: The projected density of states (PDOS) accompanied by the corresponding wave functions of spin-down orbitals of Ni in (a) Ni-N_4-C, (b) Ni-N_3-Ni-N_3-C, and (c) Ni-N_4-Ni-N_4-C.

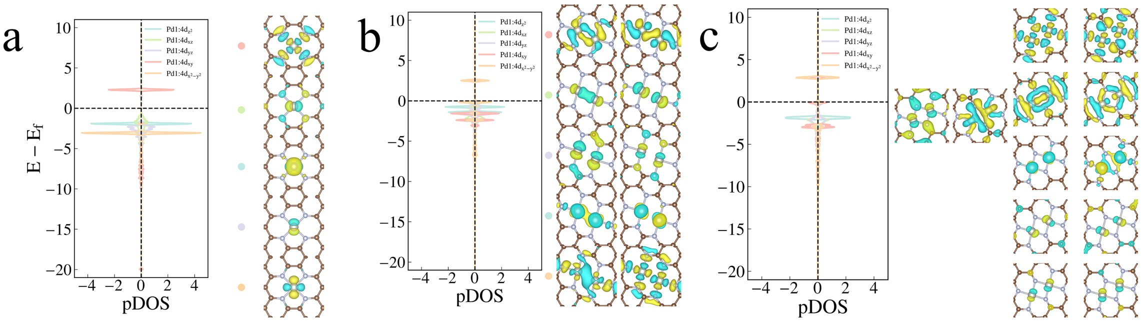

Figure S5: The projected density of states (PDOS) accompanied by the corresponding wave functions of spin-down orbitals of Pd in (a) Pd-N_4-C, (b) Pd-N_3-Pd-N_3-C, and (c) Pd-N_4-Pd-N_4-C.

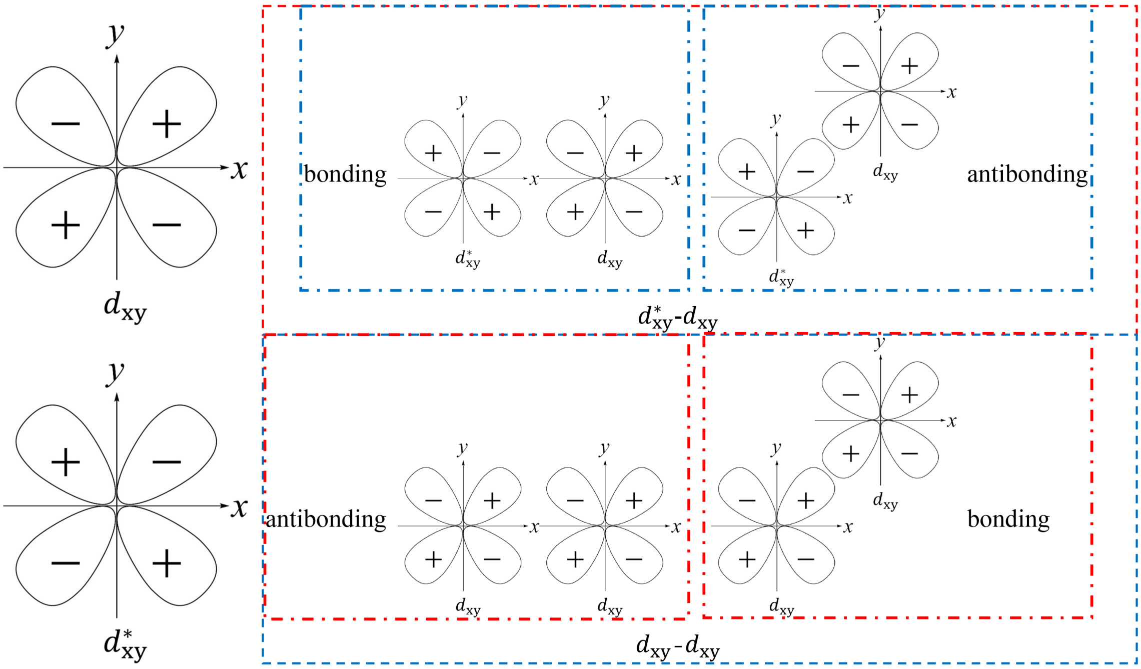

Figure S6: Schematic illustration of d/d^* atomic orbitals, and their linear combinations into bonding/antibonding molecular orbitals for varied coordination environments, with d_{xy} and d^*_{xy} as representative examples.

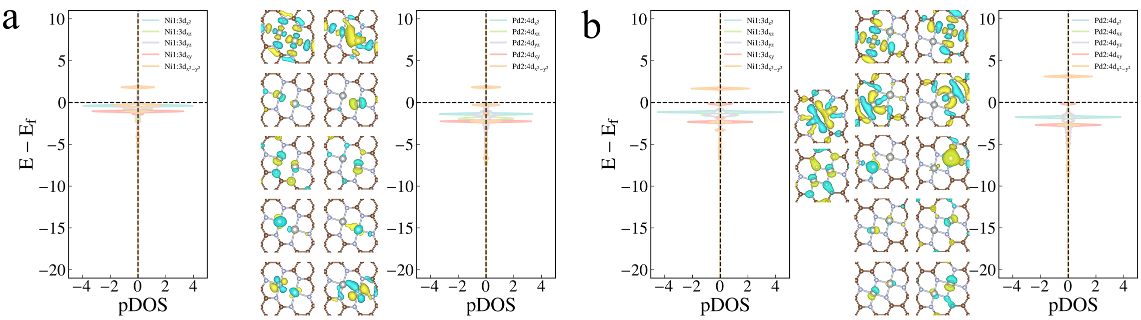

Figure S7: The projected density of states (PDOS) accompanied by the corresponding wave functions of spin-down orbitals of Ni_1 and Pd_2 sites in heteronuclear (a) Ni-N_3-Pd-N_3-C, and (b) Ni-N_4-Pd-N_4-C.

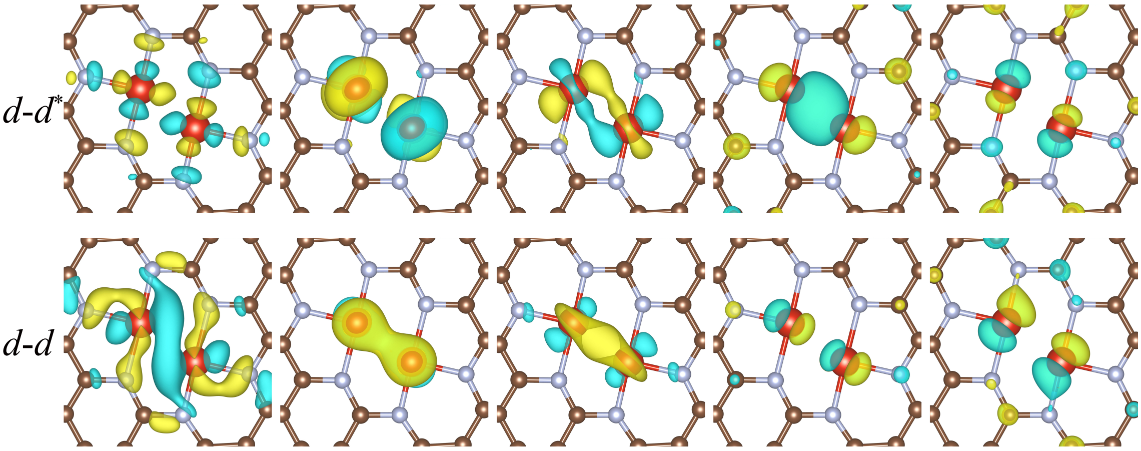

Figure S8: Wave functions for linearly combined d orbitals of the V-N_3-V-N_3-C configuration.

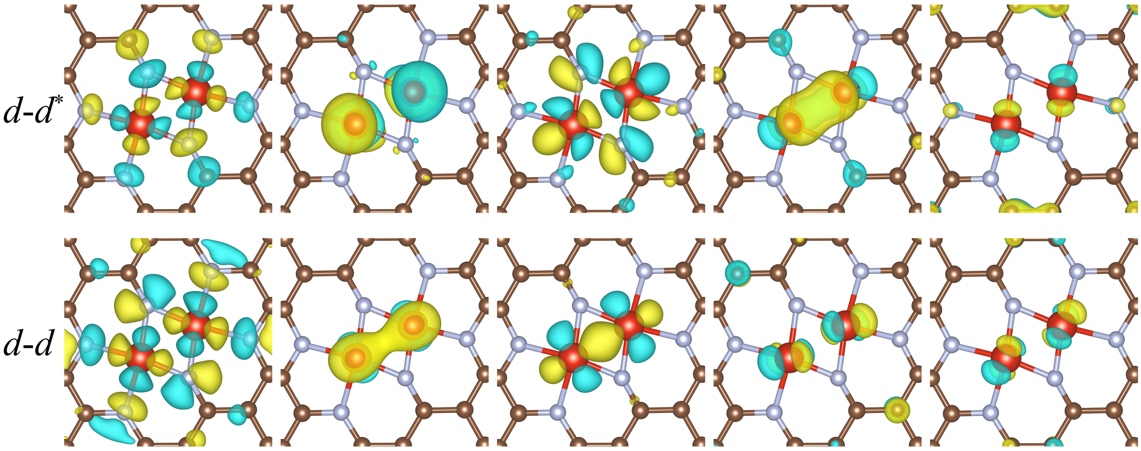

Figure S9: Wave functions for linearly combined d orbitals of the V-N_4-V-N_4-C configuration.

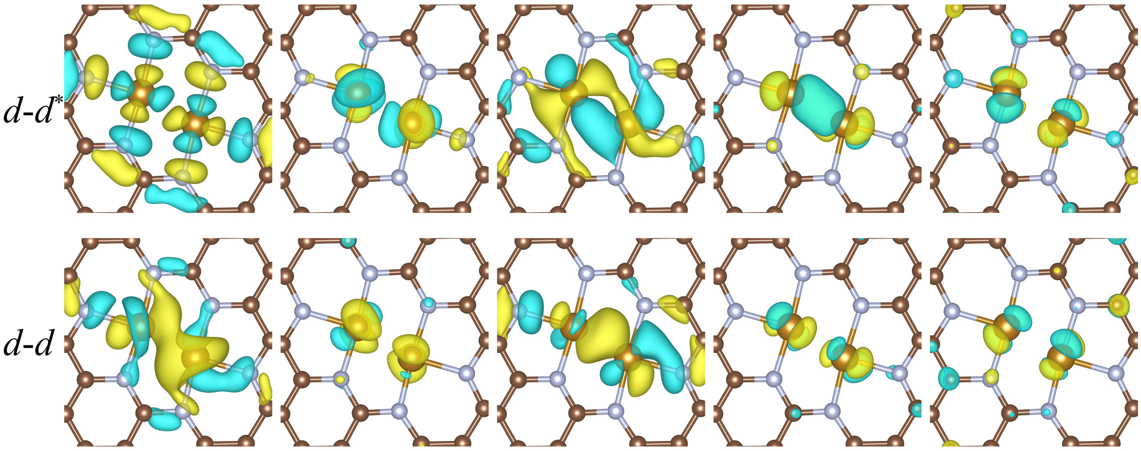

Figure S10: Wave functions for linearly combined d orbitals of the Fe-N_3-Fe-N_3-C configuration.

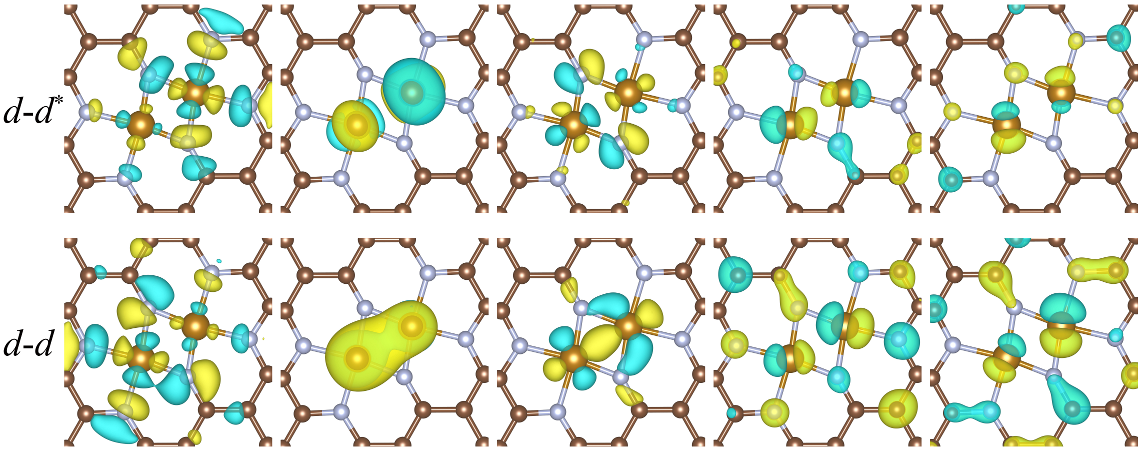

Figure S11: Wave functions for linearly combined d orbitals of the Fe-N_4-Fe-N_4-C configuration.

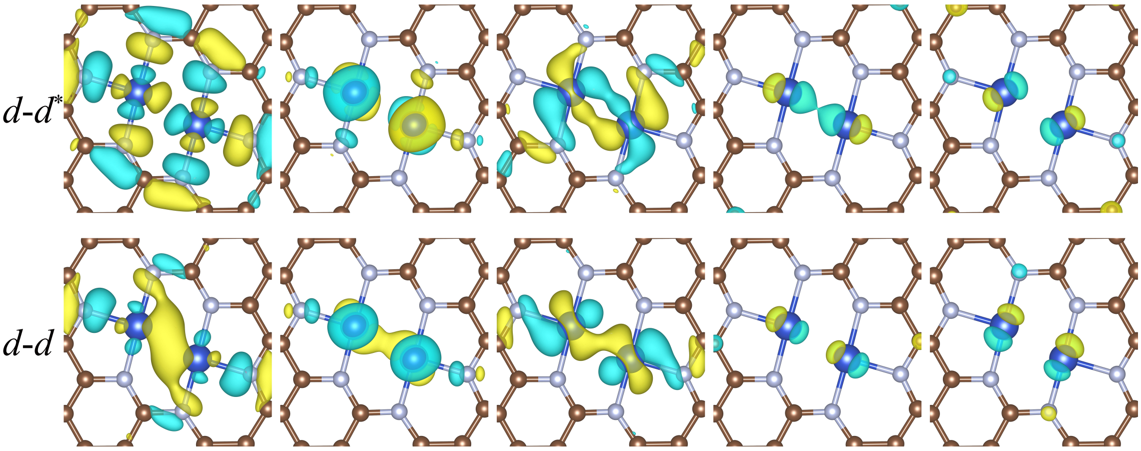

Figure S12: Wave functions for linearly combined d orbitals of the Cu-N_3-Cu-N_3-C configuration.

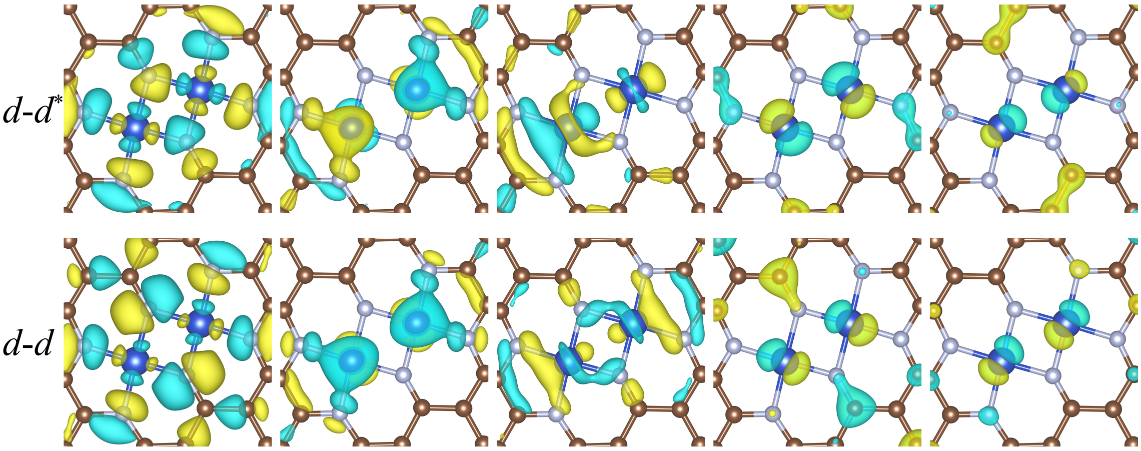

Figure S13: Wave functions for linearly combined d orbitals of the Cu-N_4-Cu-N_4-C configuration.

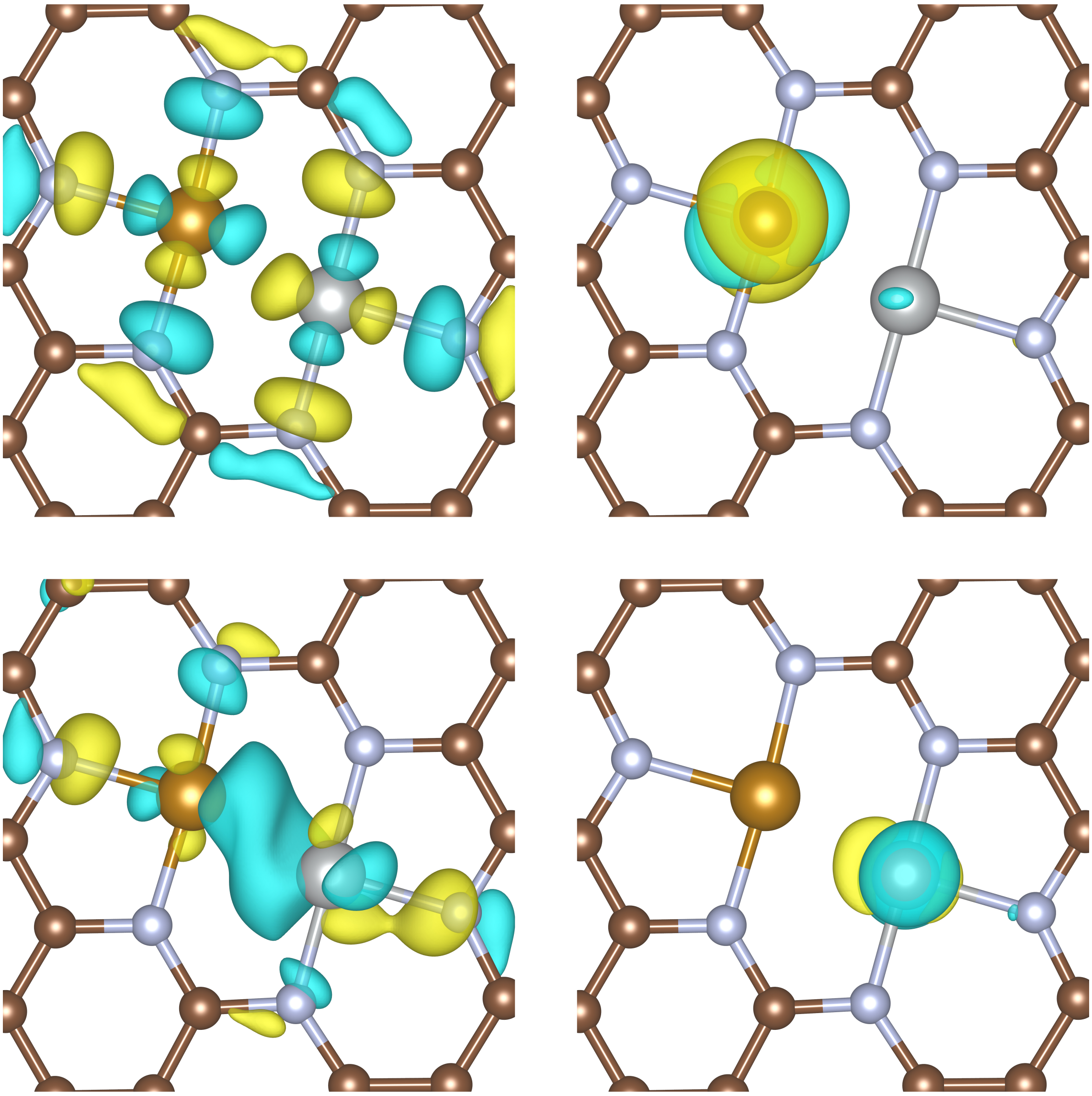

Figure S14: Wave functions for linearly combined d_{z^2} and $d_{x^2-y^2}$ orbitals in Fe-N_3-Ni-N_3-C.

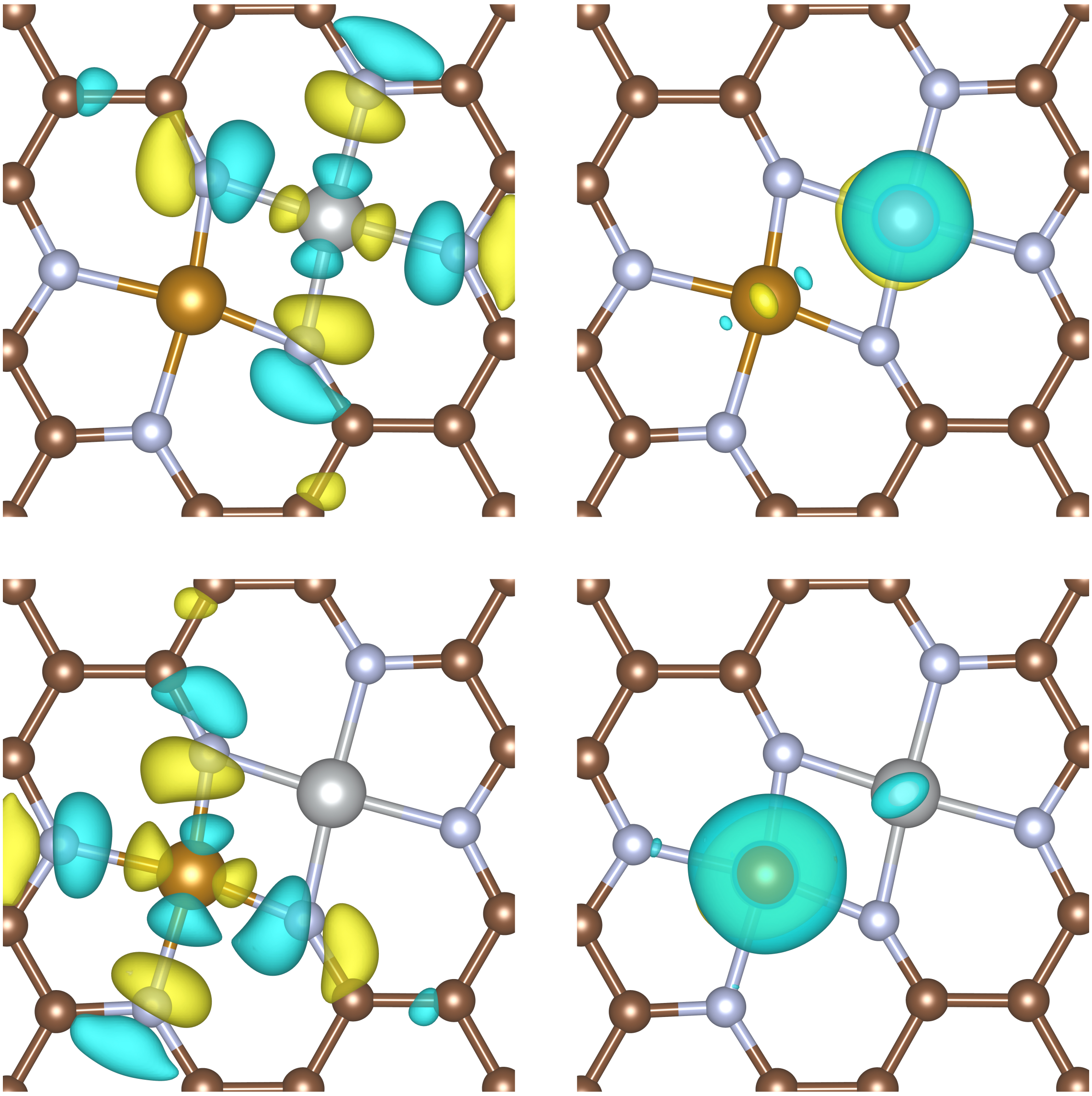

Figure S15: Wave functions for linearly combined d_{z^2} and $d_{x^2-y^2}$ orbitals in Fe-N_4-Ni-N_4-C.

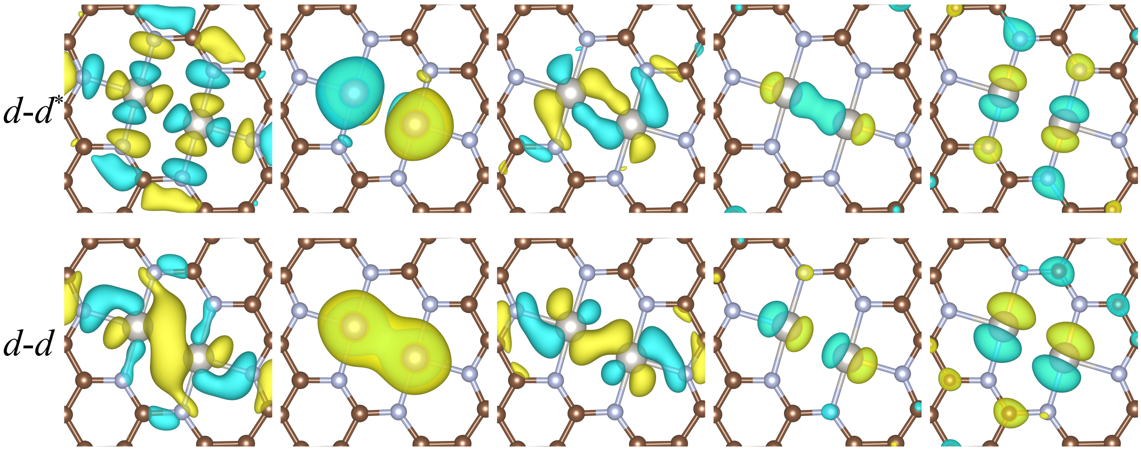

Figure S16: Wave functions for linearly combined d orbitals of the Pt-N_3-Pt-N_3-C configuration.

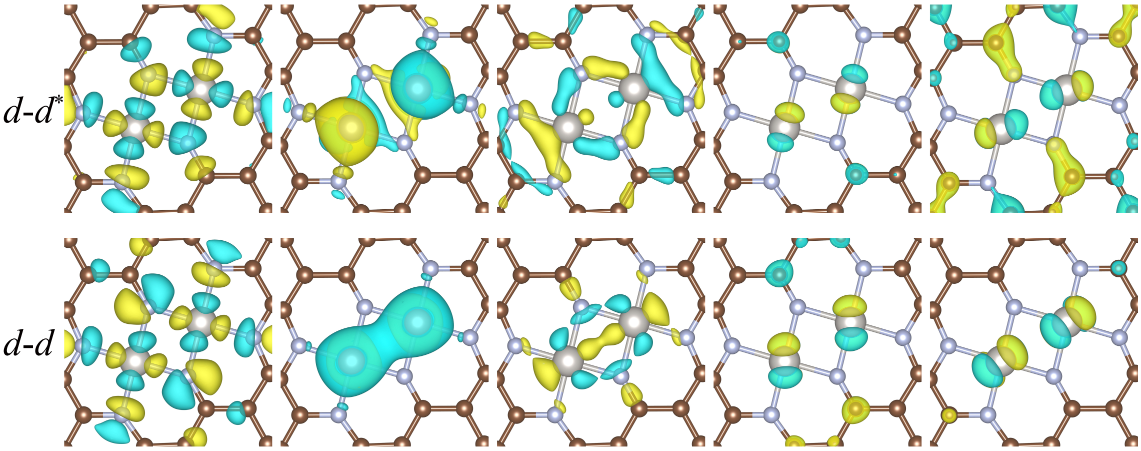

Figure S17: Wave functions for linearly combined d orbitals of the Pt-N_4-Pt-N_4-C configuration.

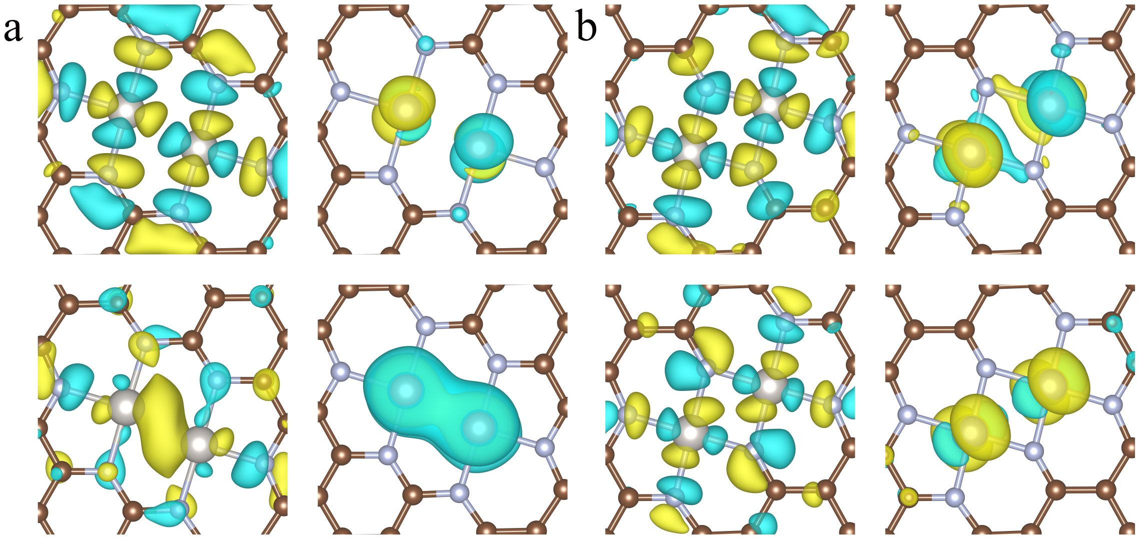

Figure S18: Wave functions for linearly combined d_{z^2} and $d_{x^2-y^2}$ orbitals in (a) Pt-N_3-Pt-N_3-C, and (b) Pt-N_4-Pt-N_4-C under SOC effects.

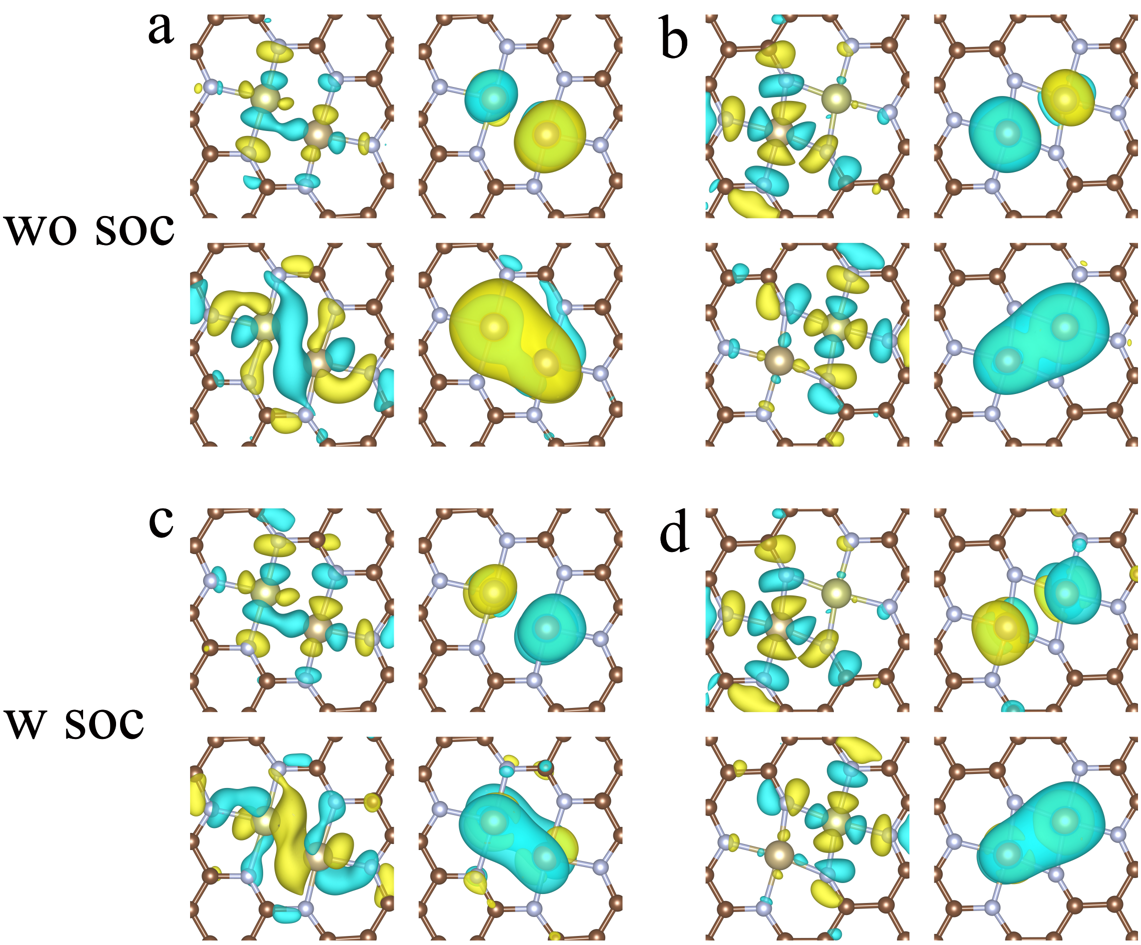

Figure S19: Wave functions for linearly combined d_{z^2} and $d_{x^2-y^2}$ orbitals in (a,c) Ir-N_3-Os-N_3-C, and (b,d) Os-N_4-Ir-N_4-C with/without SOC effects.

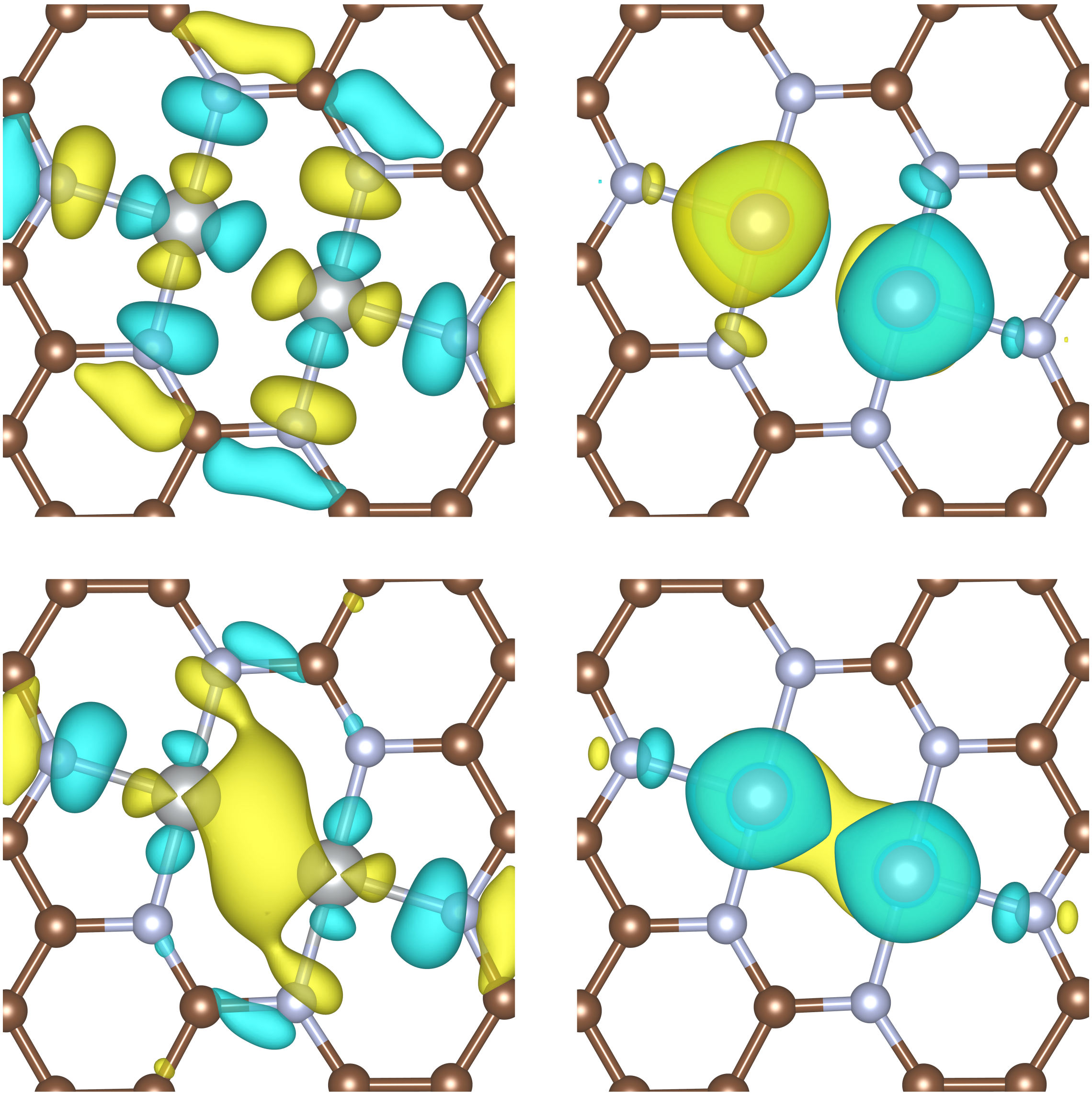

Figure S20: Wave functions for linearly combined d_{z^2} and $d_{x^2-y^2}$ orbitals in Ni-N_3-Ni-N_3-C with the DFT+U method.

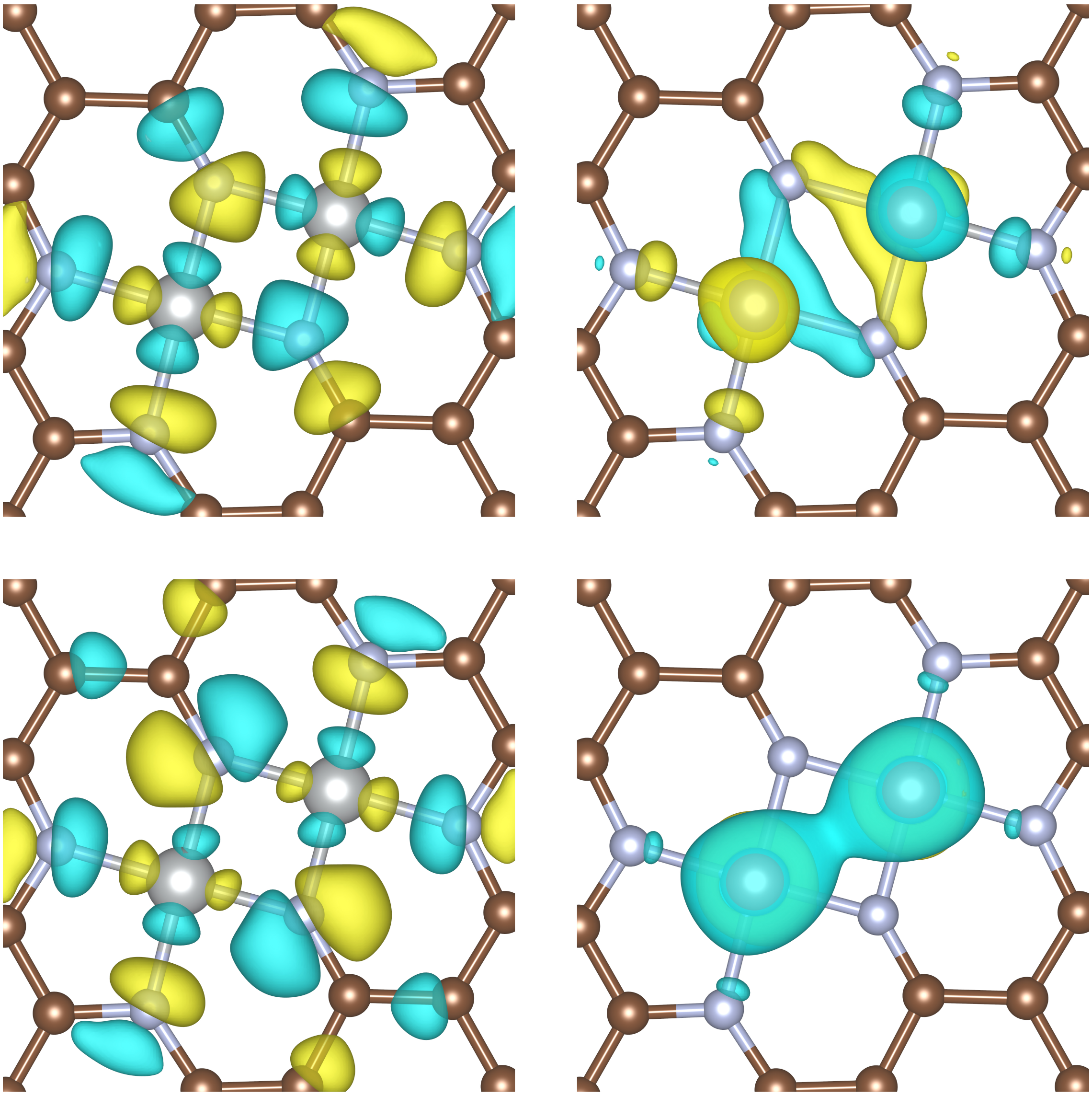

Figure S21: Wave functions for linearly combined d_{z^2} and $d_{x^2-y^2}$ orbitals in Ni-N_3-Ni-N_3-C with the DFT+U method.

S4 Identification of d orbitals and −pCOHP curves on thermodynamic stability of $M_1M_2N_6$

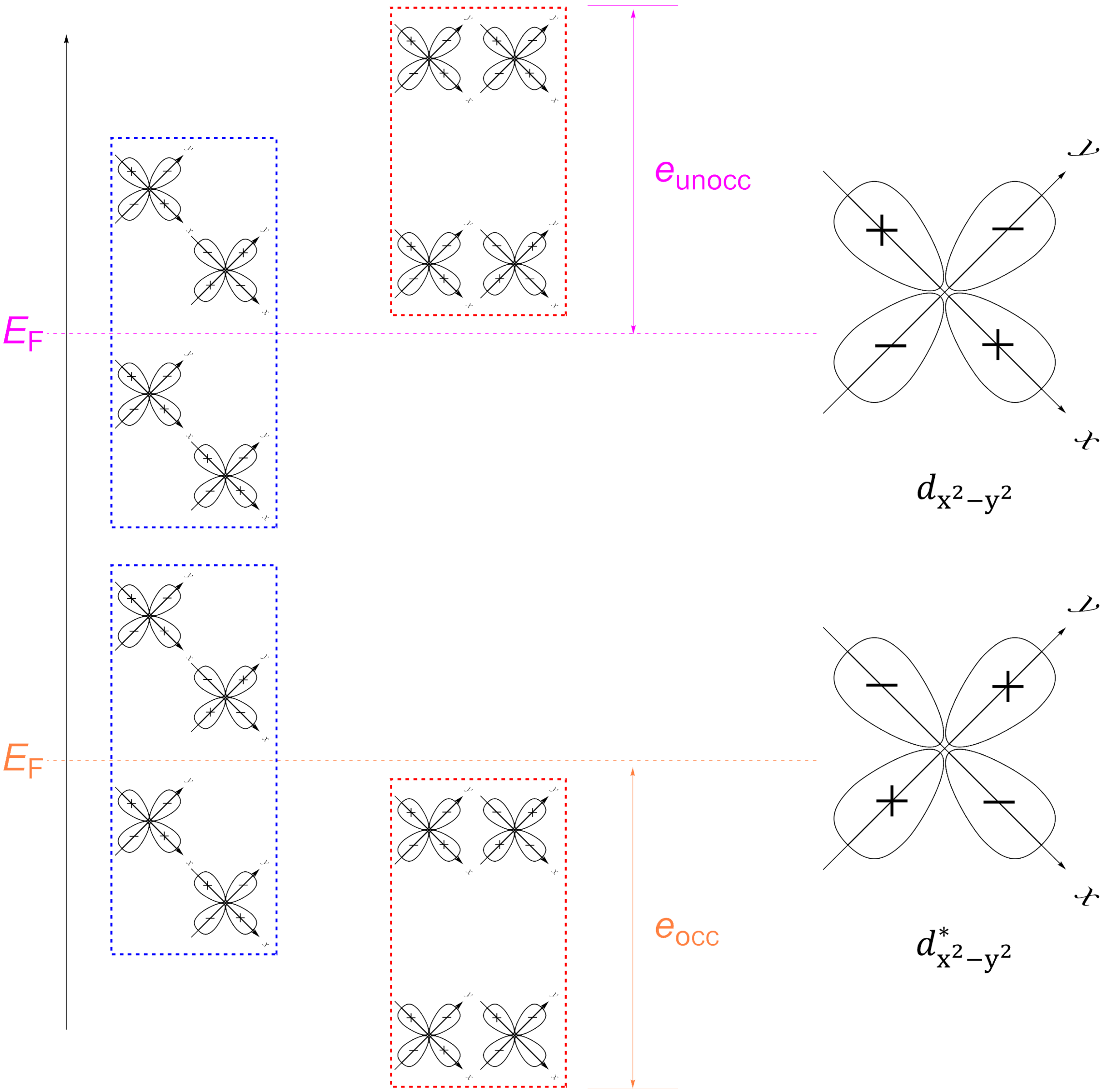

Figure S22: Schematic for linear combination of $d_{x^2-y^2}$ atomic orbitals into $d_{x^2-y^2}$-$d_{x^2-y^2}$ and $d_{x^2-y^2}$-$d^*_{x^2-y^2}$ molecular orbitals. Fully occupied/vacant combinations (red dotted rectangle) give negligible M_1-M_2 interactions, and partially occupied states (blue dotted rectangle) contribute to the M_1-M_2 interaction.

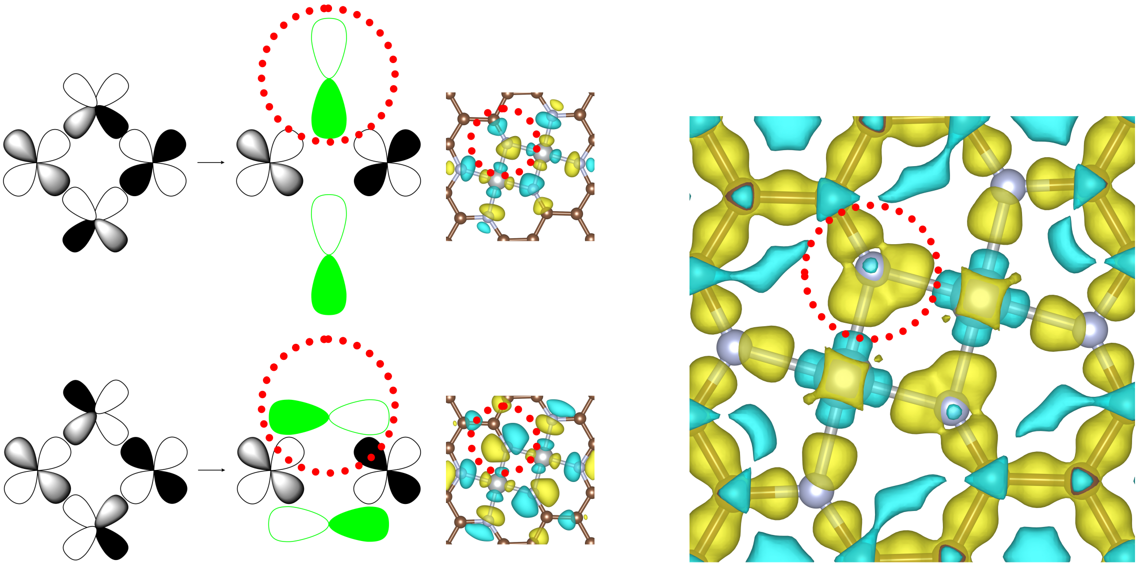

Figure S23: Schematic illustration for N_{bri} p_x/p_y orbital hybridization, spin-down wave functions and deformation charge density of Ni-N_4-Ni-N_4-C. The hybridized orbital region is circled in red dotted line.

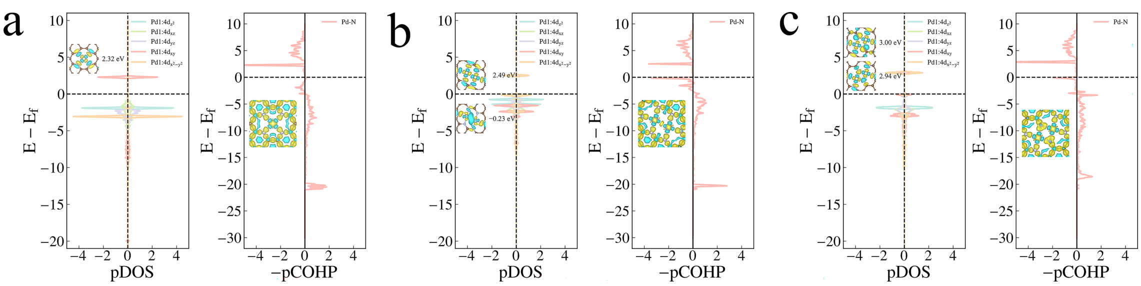

Figure S24: The PDOS (inset is the wave functions of Pd $d_{x^2-y^2}$ orbital) accompanied by the corresponding −pCOHP curves (inset is the deformation charge density) of Pd-N in (a) Pd-N_4-C, (b) Pd-N_3-Pd-N_3-C, and (c) Pd-N_4-Pd-N_4-C.

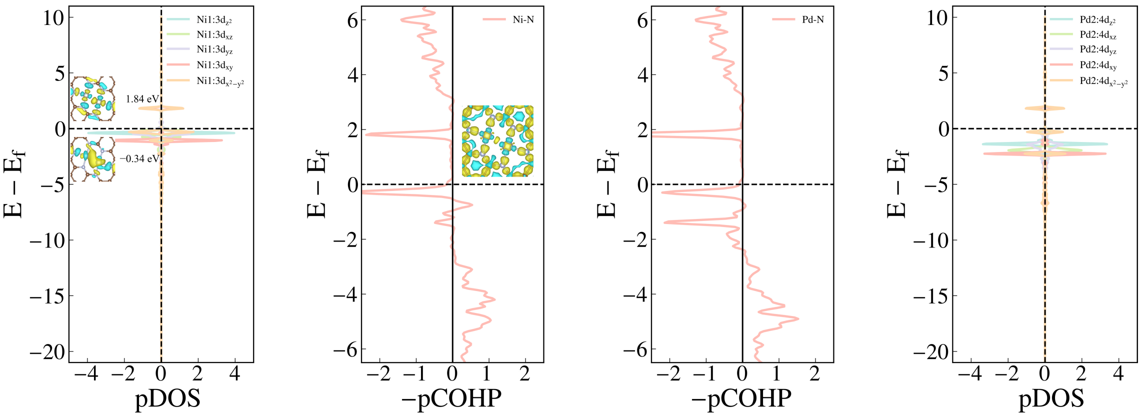

Figure S25: The PDOS (inset is the wave functions of $d_{x^2-y^2}$ orbitals of Ni_1 and Pd_2 sites) accompanied by the corresponding −pCOHP curves (inset is the deformation charge density) of Ni-N and Pd-N in Ni-N_3-Pd-N_3-C.

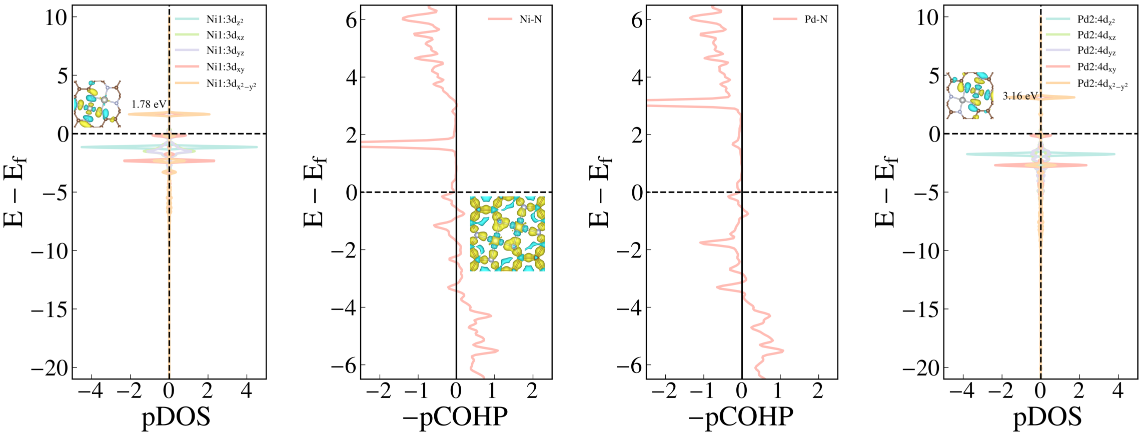

Figure S26: The PDOS (inset is the wave functions of $d_{x^2-y^2}$ orbitals of Ni_1 and Pd_2 sites) accompanied by the corresponding −pCOHP curves (inset is the deformation charge density) of Ni-N and Pd-N in Ni-N_4-Pd-N_4-C.

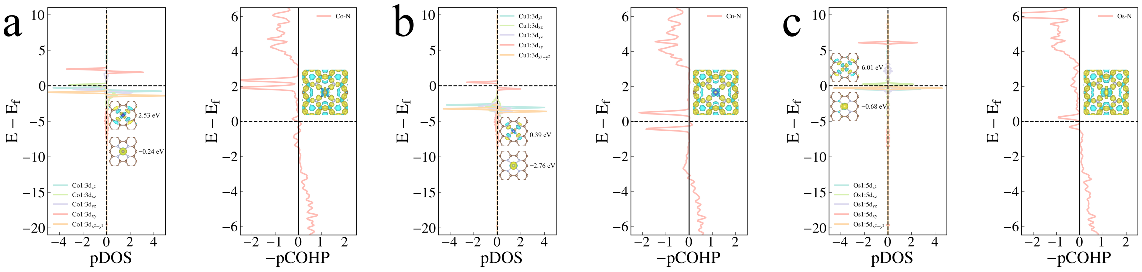

Figure S27: The PDOS (inset is the wave functions of $d_{x^2-y^2}$ and d_{z^2} orbitals of M sites) accompanied by the corresponding −pCOHP curves (inset is the deformation charge density) of M-N in (a) Co-N_4-C, (b) Cu-N_4-C, and (c)Os-N_4-C.

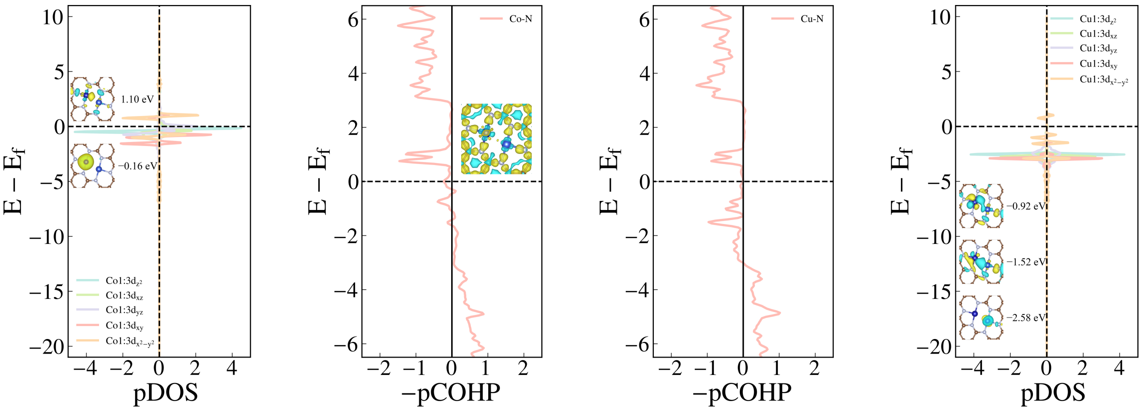

Figure S28: The PDOS (inset is the wave functions of $d_{x^2-y^2}$ and d_{z^2} orbitals of Co_1 and Cu_2 sites) accompanied by the corresponding −pCOHP curves (inset is the deformation charge density) of Co-N and Cu-N in Co-N_3-Cu-N_3-C.

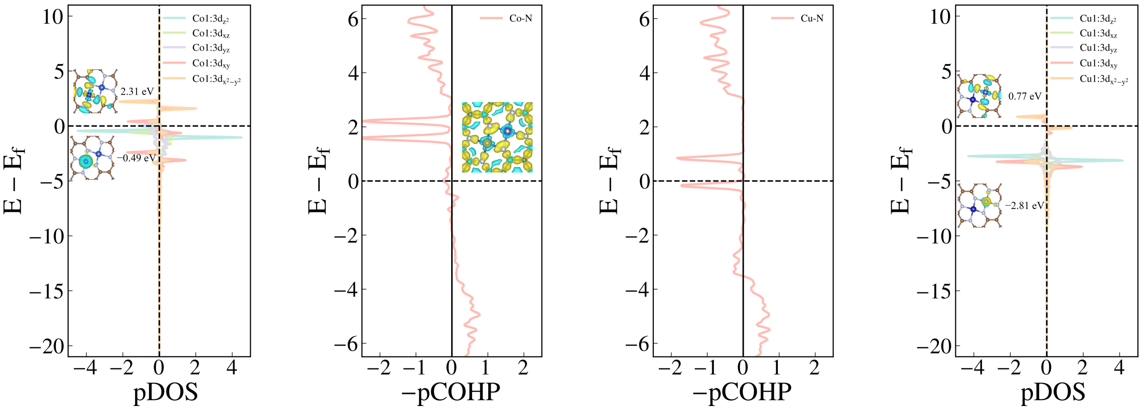

Figure S29: The PDOS (inset is the wave functions of $d_{x^2-y^2}$ and d_{z^2} orbitals of Co_1 and Cu_2 sites) accompanied by the corresponding −pCOHP curves (inset is the deformation charge density) of Co-N and Cu-N in Co-N_4-Cu-N_4-C.

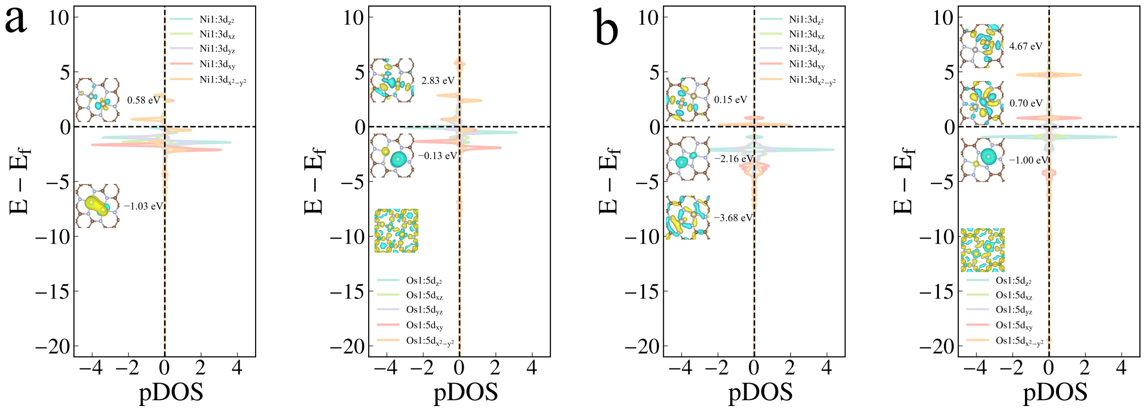

Figure S30: The PDOS (inset is the wave functions of $d_{x^2-y^2}$ and d_{z^2} orbitals of Ni and Os sites) in (a) Ni-N_3-Os-N_3-C , and (b) Ni-N_4-Os-N_4-C.

S5 Hydrogen adsorption on $M_1M_2N_6$

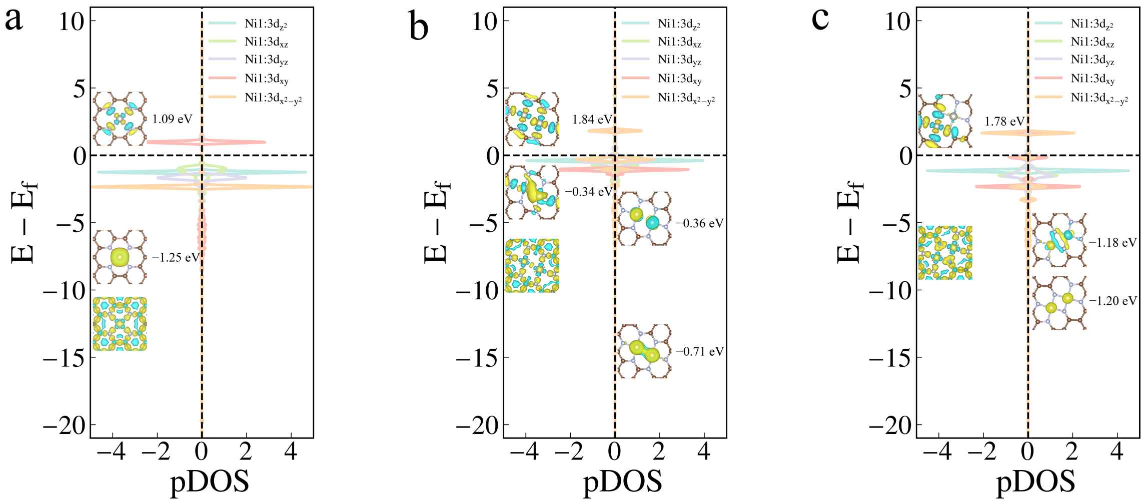

Figure S31: The PDOS (inset is the wave functions of $d_{x^2-y^2}$ and d_{z^2} orbitals of Ni sites) in (a) Ni-N_4-C, (b) Ni-N_3-Ni-N_3-C , and (c) Ni-N_4-Ni-N_4-C.

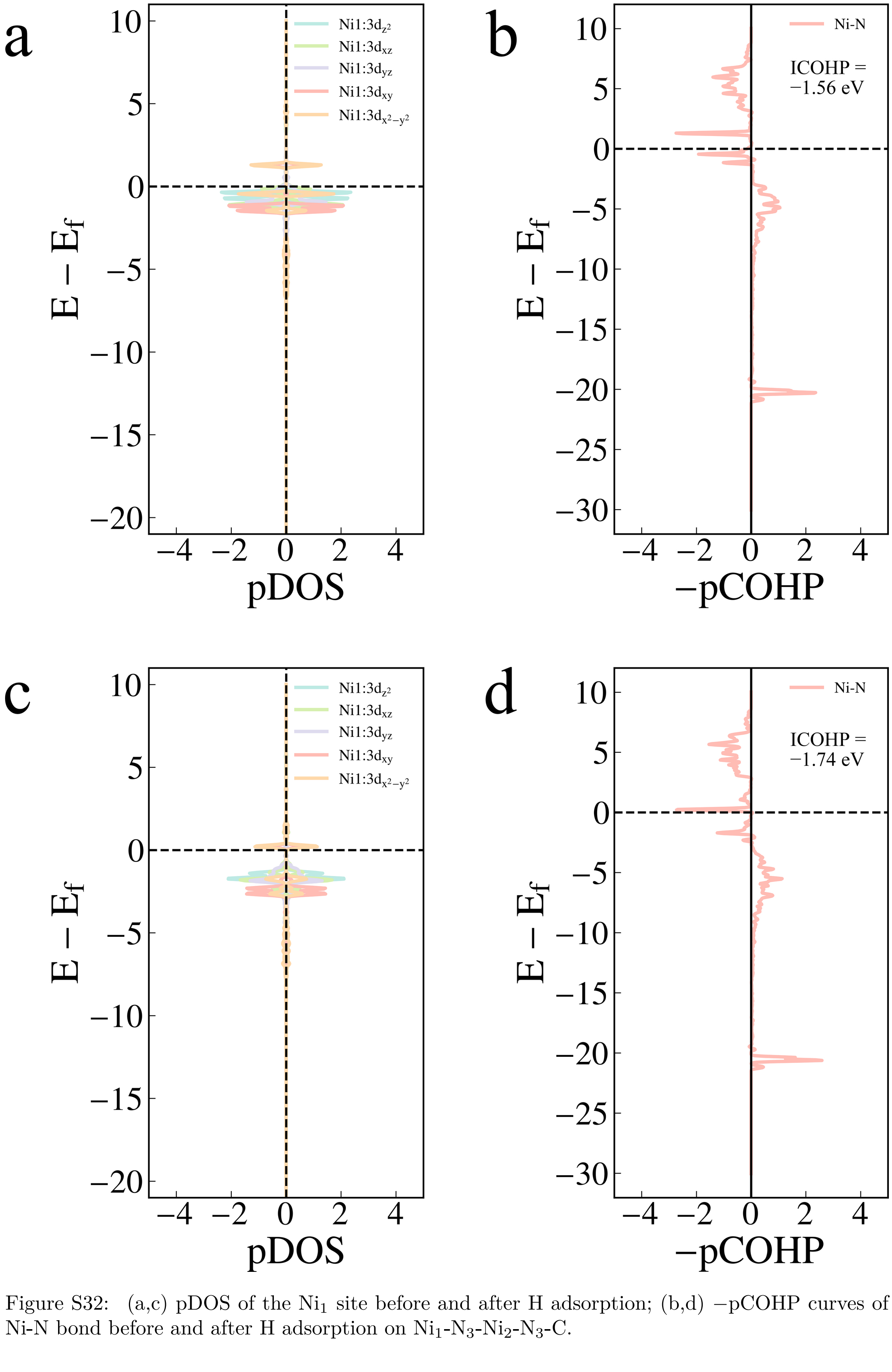

Figure S32: (a,c) pDOS of the Ni_1 site before and after H adsorption; (b,d) −pCOHP curves of Ni-N bond before and after H adsorption on Ni_1-N_3-Ni_2-N_3-C.

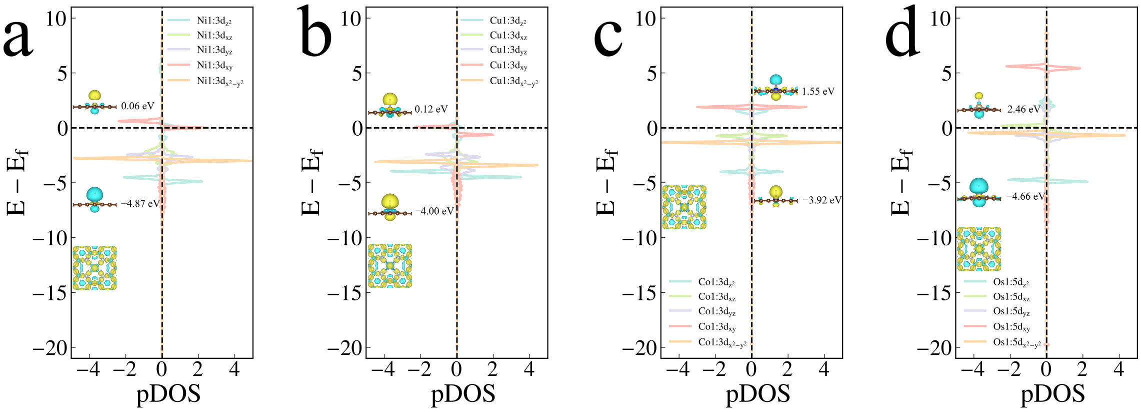

Figure S33: The PDOS (inset is the wave functions of d_{z^2} orbitals of M sites and deformation charge density) of M sites with hydrogen adsorption on (a) Ni-N_4-C, (b) Cu-N_4-C, (c) Co-N_4-C, and (d) Os-N_4-C.

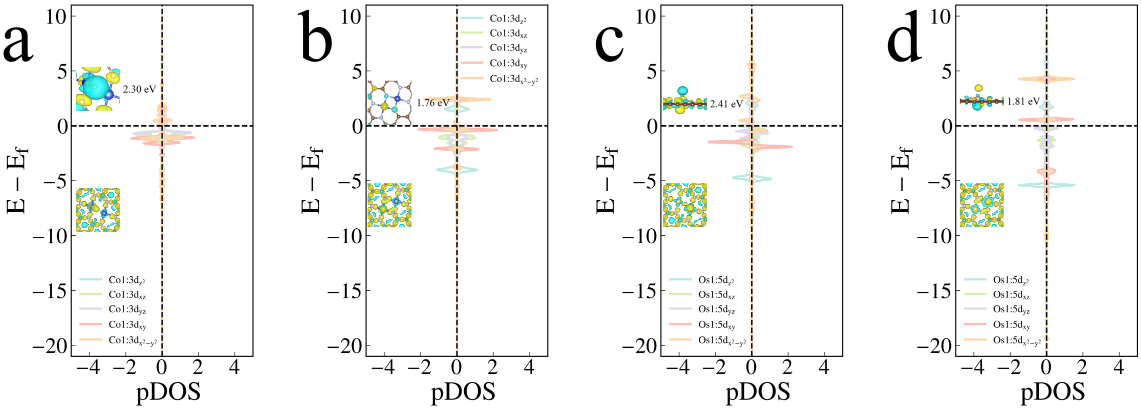

Figure S34: The PDOS (inset is the wave functions of d_{z^2} or $d_{x^2-y^2}$ orbitals of M_1 sites and deformation charge density) of M_1 sites with hydrogen adsorption on (a) Co-N_3-Cu-N_3-C, (b) Co-N_4-Cu-N_4-C, (c) Ni-N_3-Os-N_3-C, and (d) Ni-N_4-Os-N_4-C.

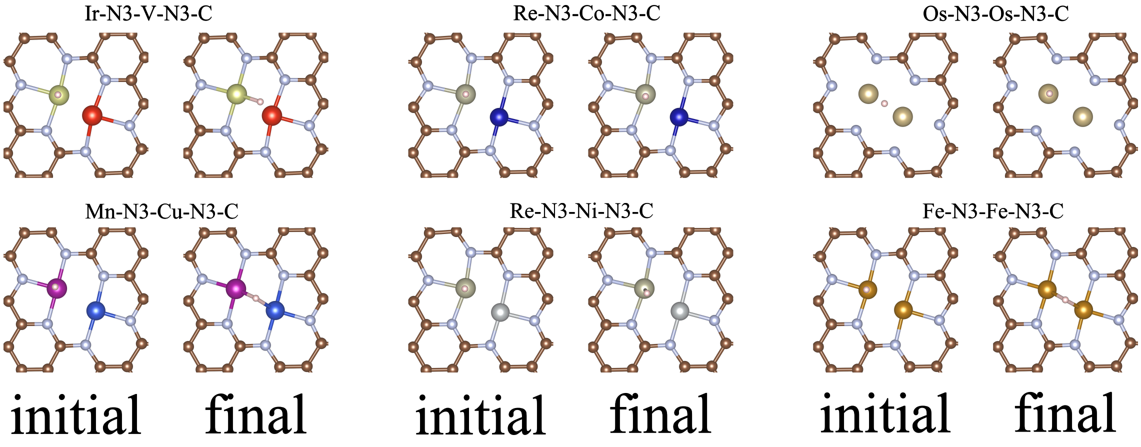

Figure S35: The initial and final structures of hydrogen adsorption on Ir-N_3-V-N_3-C, Mn-N_3-Cu-N_3-C, Re-N_3-Co-N_3-C, Re-N_3-Ni-N_3-C, Os-N_3-Os-N_3-C, and Fe-N_3-Fe-N_3-C during geometry optimization.

S6 Machine learning for $M_1M_2N_6$

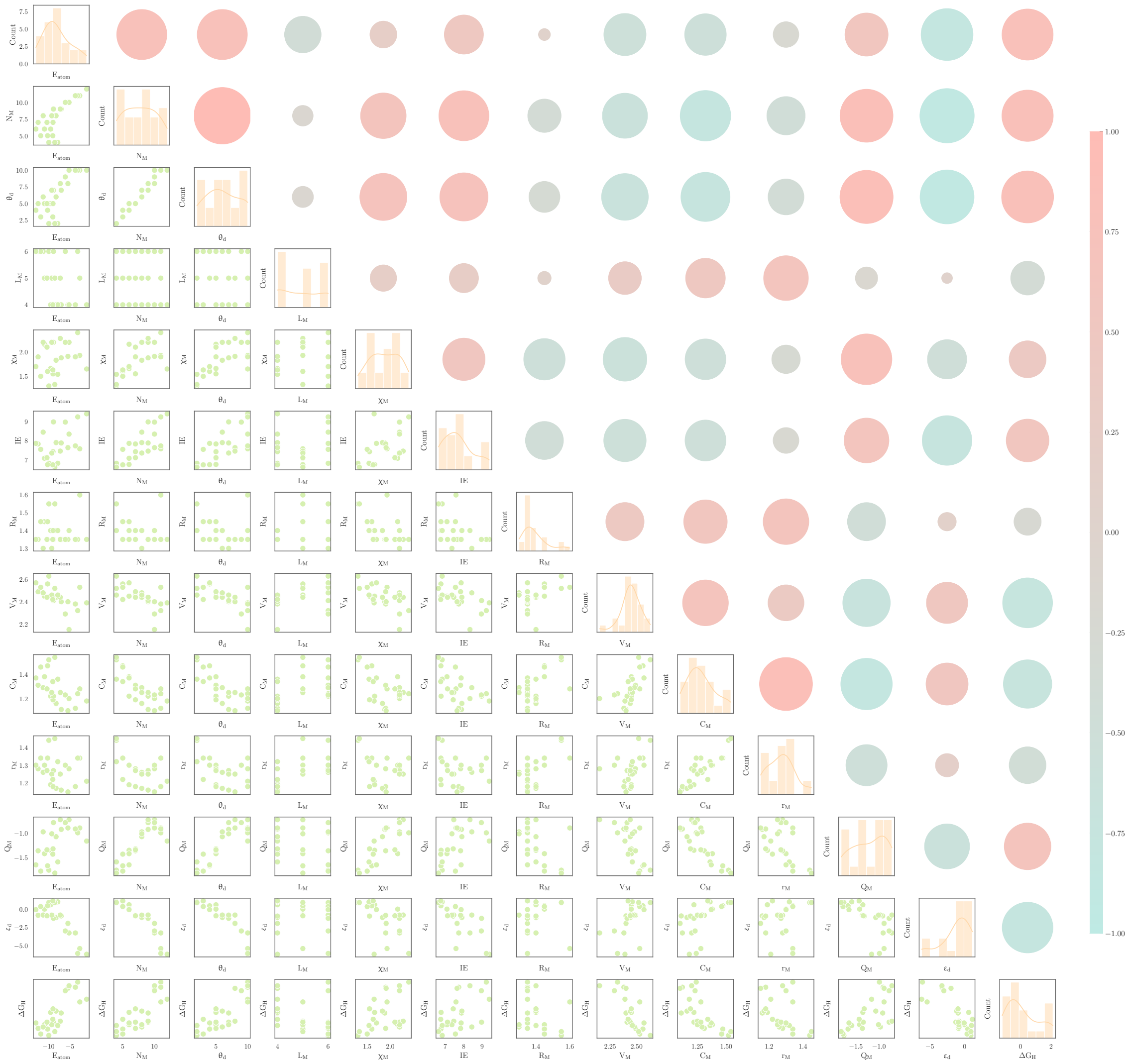

Figure S36: Collected data distribution for M-N_4-C. The diagonal portion represents the distribution curve. The lower left portion displays scatter plots that visually depicts the interrelationships between each pair of variables. The upper right portion shows the Pearson correlation coefficient, which quantifies the correlation between features or between a feature and the target variable.

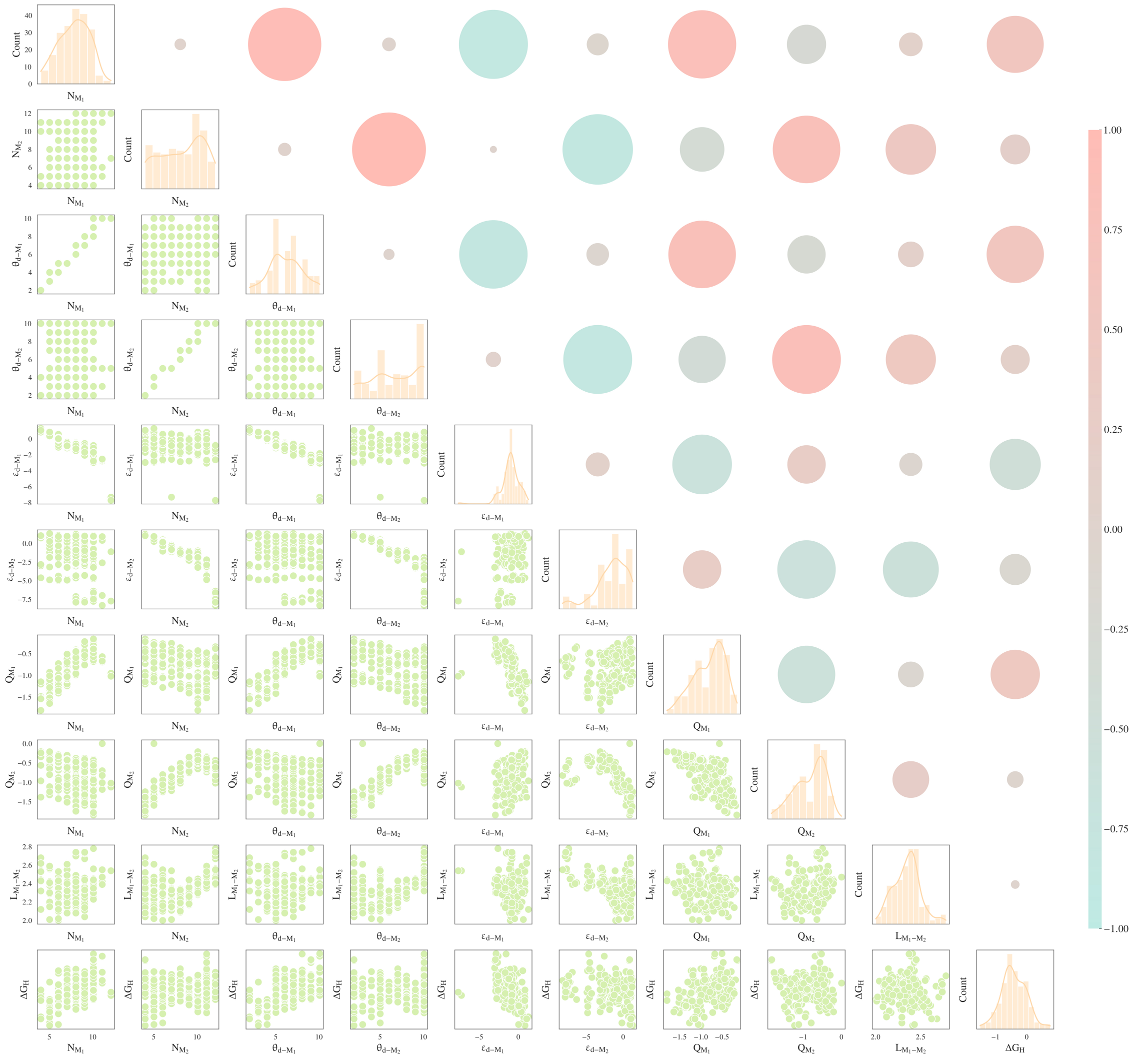

Figure S37: Collected data distribution for M_1-N_3-M_2-N_3-C.

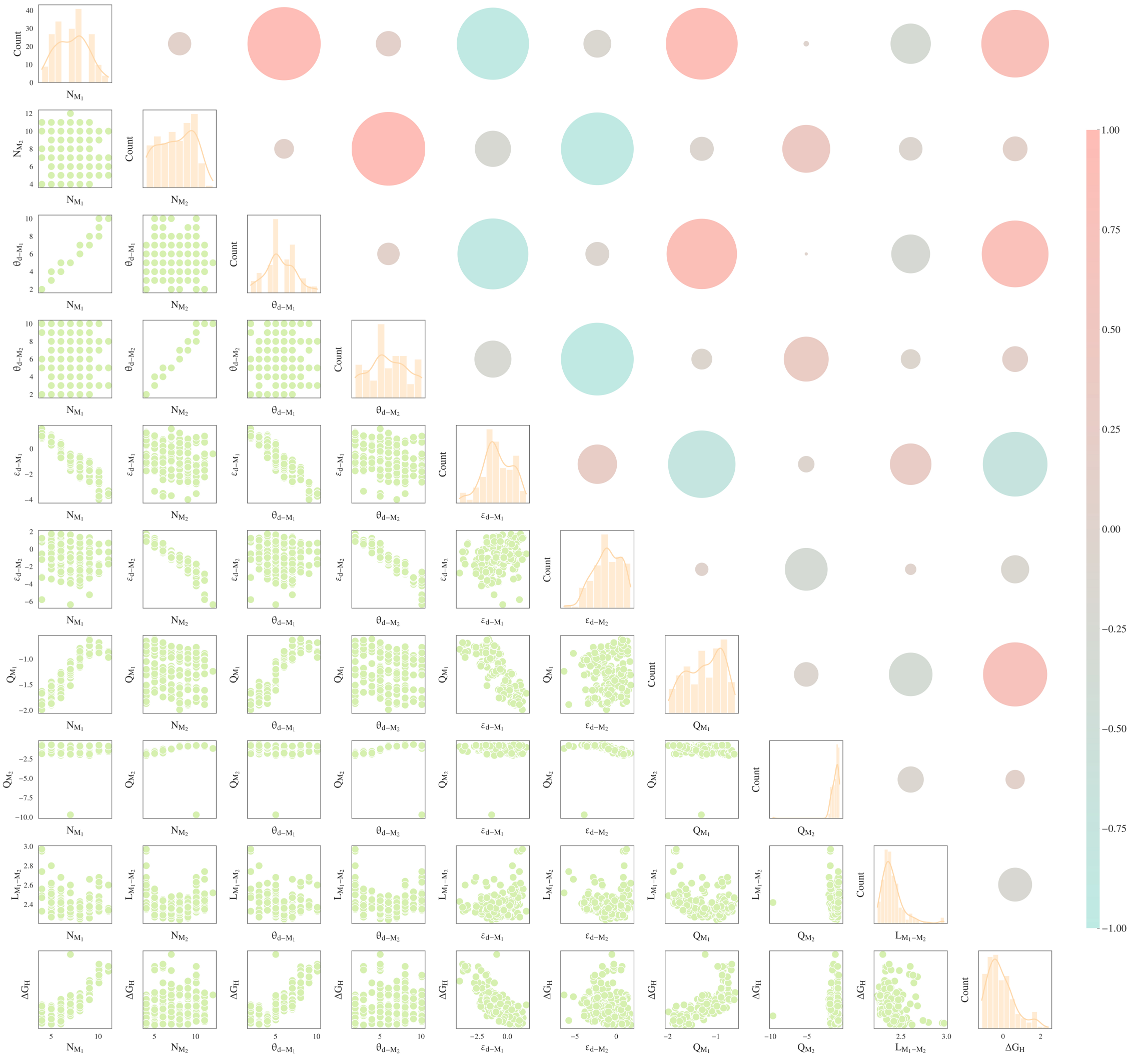

Figure S38: Collected data distribution for M_1-N_4-M_2-N_4-C.

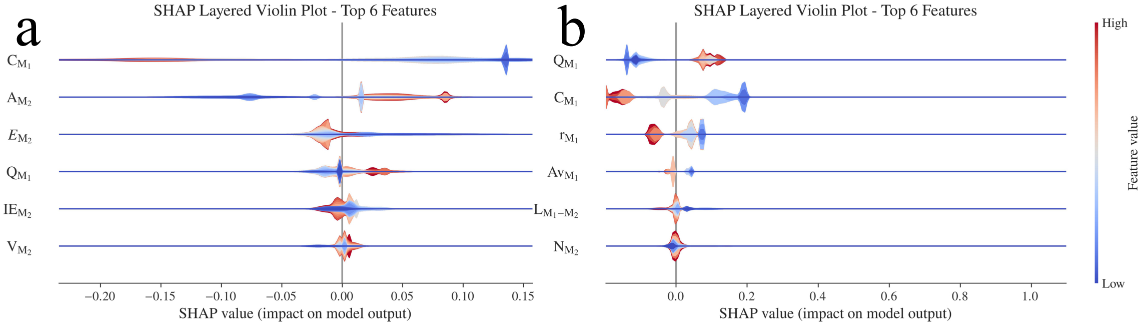

Figure S39: SHAP summary plot of the XGBoost model for ΔG_{H^*} in (a) M_1-N_3-M_2-N_3-C, and (b) M_1-N_4-M_2-N_4-C.

S7 Formation energies of M-N_4-C, and $M_1M_2N_6$.

Table S1: Formation energies of M-N_4-C

*	E_*	E_{support}	M	E_{M}	E_{f}	αE_{f}
Zn-N4-C	-1014.54	-1010.09	Zn	-1.11	-3.34	-2.50
Ag-N4-C	-1013.37	-1009.76	Ag	-2.71	-0.90	-0.68
Au-N4-C	-1014.43	-1009.56	Au	-3.19	-1.68	-1.26
Co-N4-C	-1020.64	-1009.85	Co	-7.03	-3.76	-2.82
Cr-N4-C	-1023.27	-1010.02	Cr	-9.50	-3.75	-2.81
Cu-N4-C	-1016.52	-1010.11	Cu	-3.73	-2.68	-2.01
Fe-N4-C	-1021.71	-1009.88	Fe	-8.23	-3.60	-2.70
Mn-N4-C	-1022.84	-1009.87	Mn	-8.94	-4.03	-3.02
Mo-N4-C	-1021.93	-1009.64	Mo	-10.93	-1.36	-1.02
Hf-N4-C	-1023.60	-1009.82	Hf	-9.92	-3.86	-2.90
Nb-N4-C	-1022.31	-1009.69	Nb	-10.21	-2.41	-1.81
Re-N4-C	-1022.64	-1009.46	Re	-12.42	-0.76	-0.57
Ni-N4-C	-1019.40	-1009.94	Ni	-5.47	-3.99	-2.99
Os-N4-C	-1021.97	-1009.42	Os	-11.24	-1.31	-0.98
Pd-N4-C	-1018.40	-1009.93	Pd	-5.21	-3.26	-2.44
Ir-N4-C	-1021.07	-1009.70	Ir	-8.84	-2.53	-1.90
Rh-N4-C	-1019.89	-1009.86	Rh	-7.25	-2.78	-2.08
Ru-N4-C	-1020.71	-1009.76	Ru	-9.23	-1.72	-1.29
Ta-N4-C	-1023.69	-1009.51	Ta	-11.81	-2.37	-1.78
Tc-N4-C	-1021.57	-1009.68	Tc	-10.35	-1.54	-1.16
Ti-N4-C	-1021.86	-1009.95	Ti	-7.83	-4.08	-3.06
W-N4-C	-1023.33	-1009.15	W	-12.94	-1.24	-0.93
Zr-N4-C	-1022.18	-1009.86	Zr	-8.52	-3.80	-2.85
Pt-N4-C	-1019.54	-1009.81	Pt	-6.08	-3.65	-2.74
V-N4-C	-1022.41	-1009.88	V	-8.99	-3.54	-2.66
Cd-N4-C	-1012.54	-1010.02	Cd	-0.74	-1.78	-1.34

Table S2: Formation energies of M_1-N_3-M_2-N_3-C

*	E_*	E_{support}	M_1	E_{M_1}	M_2	E_{M_2}	E_{f}	αE_{f}
Co-N3-Cu-N3-C	-1001.86	-988.13	Co	-7.03	Cu	-3.73	-1.49	-1.49
Co-N3-Co-N3-C	-1004.87	-988.11	Co	-7.03	Co	-7.03	-1.35	-1.35
Cr-N3-Co-N3-C	-1007.32	-988.11	Co	-7.03	Cr	-9.50	-1.34	-1.34
Cr-N3-Cr-N3-C	-1009.72	-987.98	Cr	-9.50	Cr	-9.50	-1.37	-1.37
Cr-N3-Cu-N3-C	-1004.82	-988.06	Cu	-3.73	Cr	-9.50	-1.76	-1.76
Cr-N3-Mn-N3-C	-1009.37	-987.93	Mn	-8.94	Cr	-9.50	-1.50	-1.50
Cu-N3-Cu-N3-C	-998.69	-988.20	Cu	-3.73	Cu	-3.73	-1.51	-1.51
Cu-N3-Zn-N3-C	-996.64	-988.08	Cu	-3.73	Zn	-1.11	-1.86	-1.86
Fe-N3-Co-N3-C	-1006.26	-988.06	Co	-7.03	Fe	-8.23	-1.47	-1.47
Fe-N3-Cr-N3-C	-1008.30	-988.04	Fe	-8.23	Cr	-9.50	-1.27	-1.27
Fe-N3-Cu-N3-C	-1002.88	-988.02	Fe	-8.23	Cu	-3.73	-1.45	-1.45
Fe-N3-Fe-N3-C	-1007.17	-988.01	Fe	-8.23	Fe	-8.23	-1.35	-1.35
Fe-N3-Mn-N3-C	-1008.04	-988.01	Fe	-8.23	Mn	-8.94	-1.43	-1.43
Fe-N3-Ni-N3-C	-1004.92	-988.09	Fe	-8.23	Ni	-5.47	-1.57	-1.57
Fe-N3-Ti-N3-C	-1007.91	-987.99	Fe	-8.23	Ti	-7.83	-1.93	-1.93
Fe-N3-V-N3-C	-1007.86	-987.99	Fe	-8.23	V	-8.99	-1.32	-1.32
Fe-N3-Zn-N3-C	-1000.21	-988.04	Fe	-8.23	Zn	-1.11	-1.41	-1.41
Ir-N3-Co-N3-C	-1005.66	-987.95	Ir	-8.84	Co	-7.03	-0.92	-0.92
Ir-N3-Cr-N3-C	-1008.35	-987.80	Ir	-8.84	Cr	-9.50	-1.10	-1.10
Ir-N3-Cu-N3-C	-1002.72	-988.01	Ir	-8.84	Cu	-3.73	-1.07	-1.07
Ir-N3-Fe-N3-C	-1007.05	-987.86	Ir	-8.84	Fe	-8.23	-1.06	-1.06

Table S2: Formation Energies of M_1-N_3-M_2-N_3-C

*	E_*	$E_{support}$	M_1	E_{M_1}	M_2	E_{M_2}	E_f	αE_f
Ir-N3-Mn-N3-C	-1008.17	-987.71	Ir	-8.84	Mn	-8.94	-1.34	-1.34
Ir-N3-Mo-N3-C	-1008.61	-987.46	Mo	-10.93	Ir	-8.84	-0.69	-0.69
Ir-N3-Ni-N3-C	-1004.46	-987.94	Ir	-8.84	Ni	-5.47	-1.10	-1.10
Ir-N3-Pt-N3-C	-1004.51	-987.49	Ir	-8.84	Pt	-6.08	-1.05	-1.05
Ir-N3-Ru-N3-C	-1006.81	-987.65	Ir	-8.84	Ru	-9.23	-0.55	-0.55
Ir-N3-V-N3-C	-1008.26	-987.59	Ir	-8.84	V	-8.99	-1.42	-1.42
Mn-N3-Co-N3-C	-1007.14	-988.08	Co	-7.03	Mn	-8.94	-1.54	-1.54
Mn-N3-Cu-N3-C	-1004.07	-987.95	Mn	-8.94	Cu	-3.73	-1.72	-1.72
Mn-N3-Mn-N3-C	-1008.96	-987.95	Mn	-8.94	Mn	-8.94	-1.56	-1.56
Mn-N3-Zn-N3-C	-1001.41	-987.90	Zn	-1.11	Mn	-8.94	-1.73	-1.73
Mo-N3-Co-N3-C	-1007.61	-987.84	Mo	-10.93	Co	-7.03	-0.91	-0.91
Mo-N3-Cr-N3-C	-1009.92	-987.84	Mo	-10.93	Cr	-9.50	-0.82	-0.82
Mo-N3-Cu-N3-C	-1003.82	-987.87	Mo	-10.93	Cu	-3.73	-0.65	-0.65
Mo-N3-Fe-N3-C	-1008.73	-987.71	Mo	-10.93	Fe	-8.23	-0.93	-0.93
Mo-N3-Mn-N3-C	-1009.67	-987.73	Mo	-10.93	Mn	-8.94	-1.04	-1.04
Mo-N3-Mo-N3-C	-1010.04	-987.06	Mo	-10.93	Mo	-10.93	-0.56	-0.56
Mo-N3-Ni-N3-C	-1006.04	-987.82	Mo	-10.93	Ni	-5.47	-0.91	-0.91
Mo-N3-V-N3-C	-1008.55	-987.74	Mo	-10.93	V	-8.99	-0.44	-0.44
Ni-N3-Co-N3-C	-1003.71	-988.10	Co	-7.03	Ni	-5.47	-1.56	-1.56
Ni-N3-Cr-N3-C	-1006.47	-988.10	Ni	-5.47	Cr	-9.50	-1.70	-1.70
Ni-N3-Cu-N3-C	-1000.70	-988.14	Ni	-5.47	Cu	-3.73	-1.68	-1.68
Ni-N3-Mn-N3-C	-1006.35	-988.04	Ni	-5.47	Mn	-8.94	-1.95	-1.95
Ni-N3-Ni-N3-C	-1002.85	-988.15	Ni	-5.47	Ni	-5.47	-1.88	-1.88
Ni-N3-Ti-N3-C	-1005.82	-987.98	Ni	-5.47	Ti	-7.83	-2.27	-2.27
Ni-N3-V-N3-C	-1006.06	-988.07	Ni	-5.47	V	-8.99	-1.76	-1.76
Ni-N3-Zn-N3-C	-998.23	-988.10	Ni	-5.47	Zn	-1.11	-1.78	-1.78
Pt-N3-Co-N3-C	-1003.84	-987.85	Co	-7.03	Pt	-6.08	-1.44	-1.44
Pt-N3-Cr-N3-C	-1006.56	-987.69	Pt	-6.08	Cr	-9.50	-1.64	-1.64
Pt-N3-Cu-N3-C	-1000.99	-987.90	Pt	-6.08	Cu	-3.73	-1.64	-1.64
Pt-N3-Fe-N3-C	-1005.08	-987.84	Pt	-6.08	Fe	-8.23	-1.47	-1.47
Pt-N3-Mn-N3-C	-1006.48	-987.72	Pt	-6.08	Mn	-8.94	-1.87	-1.87
Pt-N3-Mo-N3-C	-1006.36	-987.42	Mo	-10.93	Pt	-6.08	-0.97	-0.97
Pt-N3-Ni-N3-C	-1002.97	-987.95	Ni	-5.47	Pt	-6.08	-1.73	-1.73
Pt-N3-Pt-N3-C	-1002.81	-987.62	Pt	-6.08	Pt	-6.08	-1.51	-1.51
Pt-N3-Ru-N3-C	-1004.66	-987.52	Ru	-9.23	Pt	-6.08	-0.91	-0.91
Pt-N3-Ti-N3-C	-1005.99	-987.56	Pt	-6.08	Ti	-7.83	-2.26	-2.26
Pt-N3-V-N3-C	-1006.06	-987.67	Pt	-6.08	V	-8.99	-1.66	-1.66
Pt-N3-Zn-N3-C	-998.11	-987.81	Pt	-6.08	Zn	-1.11	-1.56	-1.56
Ru-N3-Co-N3-C	-1005.81	-988.03	Ru	-9.23	Co	-7.03	-0.76	-0.76
Ru-N3-Cr-N3-C	-1008.57	-987.94	Cr	-9.50	Ru	-9.23	-0.95	-0.95
Ru-N3-Cu-N3-C	-1002.48	-988.08	Ru	-9.23	Cu	-3.73	-0.72	-0.72
Ru-N3-Fe-N3-C	-1007.22	-987.97	Ru	-9.23	Fe	-8.23	-0.90	-0.90
Ru-N3-Mn-N3-C	-1008.15	-987.95	Mn	-8.94	Ru	-9.23	-1.02	-1.02
Ru-N3-Mo-N3-C	-1009.03	-987.40	Mo	-10.93	Ru	-9.23	-0.74	-0.74
Ru-N3-Ni-N3-C	-1004.44	-988.07	Ru	-9.23	Ni	-5.47	-0.83	-0.83
Ru-N3-Ru-N3-C	-1007.20	-987.82	Ru	-9.23	Ru	-9.23	-0.46	-0.46
Ru-N3-Ti-N3-C	-1008.24	-987.84	Ru	-9.23	Ti	-7.83	-1.67	-1.67
Ru-N3-V-N3-C	-1008.44	-987.85	V	-8.99	Ru	-9.23	-1.18	-1.18
Ti-N3-Co-N3-C	-1007.14	-987.99	Co	-7.03	Ti	-7.83	-2.15	-2.15
Ti-N3-Cr-N3-C	-1008.96	-987.87	Cr	-9.50	Ti	-7.83	-1.88	-1.88
Ti-N3-Cu-N3-C	-1003.43	-988.00	Ti	-7.83	Cu	-3.73	-1.93	-1.93
V-N3-Co-N3-C	-1007.36	-988.03	Co	-7.03	V	-8.99	-1.66	-1.66
V-N3-Cu-N3-C	-1003.92	-988.01	Cu	-3.73	V	-8.99	-1.60	-1.60
V-N3-Mn-N3-C	-1008.47	-987.90	Mn	-8.94	V	-8.99	-1.32	-1.32
V-N3-V-N3-C	-1008.70	-987.85	V	-8.99	V	-8.99	-1.43	-1.43
Zn-N3-Zn-N3-C	-993.99	-987.81	Zn	-1.11	Zn	-1.11	-1.98	-1.98
Au-N3-Co-N3-C	-1000.28	-987.75	Co	-7.03	Au	-3.19	-1.16	-1.16

Table S2: Formation Energies of M_1-N_3-M_2-N_3-C

$*$	E_*	$E_{support}$	M_1	E_{M_1}	M_2	E_{M_2}	E_f	αE_f
Au-N3-Mn-N3-C	-1002.54	-987.56	Mn	-8.94	Au	-3.19	-1.43	-1.43
Au-N3-Mo-N3-C	-1002.12	-987.32	Mo	-10.93	Au	-3.19	-0.34	-0.34
Au-N3-Pd-N3-C	-998.35	-987.60	Pd	-5.21	Au	-3.19	-1.18	-1.18
Au-N3-Tc-N3-C	-1001.48	-987.36	Tc	-10.35	Au	-3.19	-0.29	-0.29
Co-N3-Tc-N3-C	-1007.00	-987.94	Co	-7.03	Tc	-10.35	-0.84	-0.84
Cr-N3-Au-N3-C	-1003.05	-987.59	Cr	-9.50	Au	-3.19	-1.39	-1.39
Cr-N3-Rh-N3-C	-1007.45	-987.96	Rh	-7.25	Cr	-9.50	-1.37	-1.37
Cu-N3-Pd-N3-C	-1000.01	-988.00	Pd	-5.21	Cu	-3.73	-1.54	-1.54
Cu-N3-Re-N3-C	-1004.23	-987.78	Re	-12.42	Cu	-3.73	-0.15	-0.15
Cu-N3-Ta-N3-C	-1005.03	-987.67	Ta	-11.81	Cu	-3.73	-0.91	-0.91
Fe-N3-Au-N3-C	-1001.30	-987.66	Fe	-8.23	Au	-3.19	-1.11	-1.11
Fe-N3-Os-N3-C	-1008.40	-987.81	Os	-11.24	Fe	-8.23	-0.56	-0.56
Fe-N3-Re-N3-C	-1009.48	-987.69	Re	-12.42	Fe	-8.23	-0.57	-0.57
Fe-N3-Rh-N3-C	-1005.96	-988.01	Rh	-7.25	Fe	-8.23	-1.24	-1.24
Fe-N3-Tc-N3-C	-1008.15	-987.86	Tc	-10.35	Fe	-8.23	-0.86	-0.86
Fe-N3-W-N3-C	-1010.06	-987.49	W	-12.94	Fe	-8.23	-0.70	-0.70
Ir-N3-Os-N3-C	-1007.96	-987.52	Os	-11.24	Ir	-8.84	-0.18	-0.18
Ir-N3-Pd-N3-C	-1003.47	-987.71	Ir	-8.84	Pd	-5.21	-0.86	-0.86
Ir-N3-Rh-N3-C	-1005.65	-987.64	Ir	-8.84	Rh	-7.25	-0.96	-0.96
Ir-N3-Tc-N3-C	-1008.07	-987.47	Ir	-8.84	Tc	-10.35	-0.71	-0.71
Mo-N3-Os-N3-C	-1010.40	-987.15	Os	-11.24	Mo	-10.93	-0.54	-0.54
Mo-N3-Rh-N3-C	-1007.42	-987.63	Mo	-10.93	Rh	-7.25	-0.80	-0.80
Nb-N3-Au-N3-C	-1002.35	-987.36	Nb	-10.21	Au	-3.19	-0.80	-0.80
Nb-N3-Cu-N3-C	-1003.94	-987.80	Nb	-10.21	Cu	-3.73	-1.10	-1.10
Nb-N3-Fe-N3-C	-1008.86	-987.81	Fe	-8.23	Nb	-10.21	-1.31	-1.31
Nb-N3-Pd-N3-C	-1005.42	-987.60	Nb	-10.21	Pd	-5.21	-1.20	-1.20
Ni-N3-Au-N3-C	-999.32	-987.77	Ni	-5.47	Au	-3.19	-1.45	-1.45
Ni-N3-Nb-N3-C	-1006.46	-987.80	Nb	-10.21	Ni	-5.47	-1.49	-1.49
Ni-N3-Os-N3-C	-1005.58	-987.91	Os	-11.24	Ni	-5.47	-0.48	-0.48
Ni-N3-Pd-N3-C	-1002.00	-988.08	Ni	-5.47	Pd	-5.21	-1.62	-1.62
Ni-N3-Rh-N3-C	-1003.49	-988.12	Rh	-7.25	Ni	-5.47	-1.33	-1.33
Ni-N3-Tc-N3-C	-1005.48	-987.93	Tc	-10.35	Ni	-5.47	-0.87	-0.87
Os-N3-Co-N3-C	-1006.99	-987.83	Os	-11.24	Co	-7.03	-0.45	-0.45
Os-N3-Cr-N3-C	-1009.64	-987.73	Os	-11.24	Cr	-9.50	-0.58	-0.58
Os-N3-Mn-N3-C	-1009.43	-987.74	Os	-11.24	Mn	-8.94	-0.75	-0.75
Os-N3-Os-N3-C	-1009.76	-987.40	Os	-11.24	Os	-11.24	0.06	0.06
Os-N3-Pt-N3-C	-1005.84	-987.33	Os	-11.24	Pt	-6.08	-0.59	-0.59
Os-N3-Rh-N3-C	-1006.89	-987.62	Os	-11.24	Rh	-7.25	-0.39	-0.39
Os-N3-Ti-N3-C	-1009.26	-987.61	Os	-11.24	Ti	-7.83	-1.29	-1.29
Os-N3-V-N3-C	-1009.81	-987.45	Os	-11.24	V	-8.99	-1.07	-1.07
Pd-N3-Co-N3-C	-1002.86	-987.99	Co	-7.03	Pd	-5.21	-1.31	-1.31
Pd-N3-Fe-N3-C	-1004.06	-987.98	Fe	-8.23	Pd	-5.21	-1.32	-1.32
Pd-N3-Mn-N3-C	-1005.45	-987.88	Mn	-8.94	Pd	-5.21	-1.71	-1.71
Pd-N3-Mo-N3-C	-1005.24	-987.58	Mo	-10.93	Pd	-5.21	-0.76	-0.76
Pd-N3-Pd-N3-C	-1000.94	-987.91	Pd	-5.21	Pd	-5.21	-1.30	-1.30
Pd-N3-V-N3-C	-1005.03	-987.85	Pd	-5.21	V	-8.99	-1.49	-1.49
Pt-N3-Au-N3-C	-999.32	-987.46	Pt	-6.08	Au	-3.19	-1.29	-1.29
Pt-N3-Nb-N3-C	-1006.54	-987.40	Nb	-10.21	Pt	-6.08	-1.42	-1.42
Re-N3-Au-N3-C	-1002.55	-987.24	Re	-12.42	Au	-3.19	0.15	0.15
Re-N3-Co-N3-C	-1008.12	-987.78	Re	-12.42	Co	-7.03	-0.45	-0.45
Re-N3-Ir-N3-C	-1009.32	-987.27	Re	-12.42	Ir	-8.84	-0.40	-0.40
Re-N3-Ni-N3-C	-1006.35	-987.79	Re	-12.42	Ni	-5.47	-0.34	-0.34
Re-N3-Pt-N3-C	-1006.83	-987.29	Re	-12.42	Pt	-6.08	-0.52	-0.52
Re-N3-Rh-N3-C	-1008.07	-987.46	Re	-12.42	Rh	-7.25	-0.47	-0.47
Rh-N3-Co-N3-C	-1004.52	-988.13	Rh	-7.25	Co	-7.03	-1.06	-1.06
Rh-N3-Cu-N3-C	-1001.79	-988.14	Rh	-7.25	Cu	-3.73	-1.34	-1.34
Rh-N3-Mn-N3-C	-1007.07	-987.89	Rh	-7.25	Mn	-8.94	-1.49	-1.49

Table S2: Formation Energies of M_1-N_3-M_2-N_3-C

$*$	E_*	E_{support}	M_1	E_{M_1}	M_2	E_{M_2}	E_f	αE_f
Rh-N3-V-N3-C	-1007.03	-987.92	Rh	-7.25	V	-8.99	-1.43	-1.43
Ru-N3-Os-N3-C	-1008.41	-987.61	Os	-11.24	Ru	-9.23	-0.16	-0.16
Tc-N3-Cu-N3-C	-1003.26	-987.89	Tc	-10.35	Cu	-3.73	-0.65	-0.65
Tc-N3-Mn-N3-C	-1009.13	-987.72	Tc	-10.35	Mn	-8.94	-1.06	-1.06
W-N3-Cu-N3-C	-1004.83	-987.72	W	-12.94	Cu	-3.73	-0.22	-0.22
Zn-N3-Pd-N3-C	-997.29	-987.92	Pd	-5.21	Zn	-1.11	-1.52	-1.52
Ir-N3-Ir-N3-C	-1006.78	-987.45	Ir	-8.84	Ir	-8.84	-0.83	-0.83
Zr-N3-Pd-N3-C	-1005.48	-987.65	Zr	-8.52	Pd	-5.21	-2.05	-2.05
Zr-N3-Mo-N3-C	-1009.10	-987.54	Mo	-10.93	Zr	-8.52	-1.06	-1.06
W-N3-Ni-N3-C	-1007.29	-987.68	W	-12.94	Ni	-5.47	-0.60	-0.60
W-N3-Co-N3-C	-1009.01	-987.65	W	-12.94	Co	-7.03	-0.69	-0.69
Ti-N3-Au-N3-C	-1001.81	-987.68	Ti	-7.83	Au	-3.19	-1.55	-1.55
Tc-N3-V-N3-C	-1009.36	-987.60	V	-8.99	Tc	-10.35	-1.21	-1.21
Tc-N3-Ti-N3-C	-1008.88	-987.78	Tc	-10.35	Ti	-7.83	-1.46	-1.46
Tc-N3-Tc-N3-C	-1009.58	-987.38	Tc	-10.35	Tc	-10.35	-0.75	-0.75
Tc-N3-Ru-N3-C	-1008.40	-987.64	Ru	-9.23	Tc	-10.35	-0.59	-0.59
Tc-N3-Rh-N3-C	-1006.87	-987.67	Rh	-7.25	Tc	-10.35	-0.80	-0.80
Tc-N3-Pd-N3-C	-1004.48	-987.69	Tc	-10.35	Pd	-5.21	-0.61	-0.61
Tc-N3-Os-N3-C	-1009.74	-987.41	Os	-11.24	Tc	-10.35	-0.37	-0.37
Tc-N3-Mo-N3-C	-1010.00	-987.32	Tc	-10.35	Mo	-10.93	-0.70	-0.70
Au-N3-Ta-N3-C	-1003.54	-987.24	Ta	-11.81	Au	-3.19	-0.65	-0.65
Cd-N3-Ni-N3-C	-996.99	-988.21	Ni	-5.47	Cd	-0.74	-1.28	-1.28
Co-N3-Cd-N3-C	-998.14	-988.25	Co	-7.03	Cd	-0.74	-1.06	-1.06
Co-N3-Nb-N3-C	-1007.99	-987.78	Co	-7.03	Nb	-10.21	-1.49	-1.49
Cr-N3-Pd-N3-C	-1005.57	-987.89	Pd	-5.21	Cr	-9.50	-1.49	-1.49
Cr-N3-Tc-N3-C	-1009.55	-987.77	Tc	-10.35	Cr	-9.50	-0.97	-0.97
Cu-N3-Cd-N3-C	-995.11	-988.14	Cu	-3.73	Cd	-0.74	-1.25	-1.25
Fe-N3-Cd-N3-C	-999.33	-988.21	Fe	-8.23	Cd	-0.74	-1.07	-1.07
Hf-N3-Ni-N3-C	-1007.65	-987.76	Ni	-5.47	Hf	-9.92	-2.25	-2.25
Hf-N3-Pd-N3-C	-1006.76	-987.49	Hf	-9.92	Pd	-5.21	-2.07	-2.07
Hf-N3-Pt-N3-C	-1007.85	-987.29	Pt	-6.08	Hf	-9.92	-2.28	-2.28
Hf-N3-Ru-N3-C	-1009.89	-987.65	Ru	-9.23	Hf	-9.92	-1.55	-1.55
Ir-N3-Cd-N3-C	-999.15	-988.02	Ir	-8.84	Cd	-0.74	-0.78	-0.78
Ir-N3-Nb-N3-C	-1009.07	-987.33	Ir	-8.84	Nb	-10.21	-1.35	-1.35
Mn-N3-Re-N3-C	-1010.39	-987.52	Re	-12.42	Mn	-8.94	-0.76	-0.76
Mo-N3-Nb-N3-C	-1010.09	-987.21	Mo	-10.93	Nb	-10.21	-0.87	-0.87
Mo-N3-Ta-N3-C	-1010.59	-987.62	Ta	-11.81	Mo	-10.93	-0.11	-0.11
Mo-N3-W-N3-C	-1010.98	-987.16	W	-12.94	Mo	-10.93	0.02	0.02
Nb-N3-Nb-N3-C	-1009.85	-987.49	Nb	-10.21	Nb	-10.21	-0.97	-0.97
Nb-N3-Ti-N3-C	-1008.65	-987.76	Ti	-7.83	Nb	-10.21	-1.42	-1.42
Nb-N3-Zr-N3-C	-1009.08	-987.74	Nb	-10.21	Zr	-8.52	-1.31	-1.31
Os-N3-Cd-N3-C	-1000.29	-987.85	Os	-11.24	Cd	-0.74	-0.23	-0.23
Os-N3-Hf-N3-C	-1010.93	-987.37	Os	-11.24	Hf	-9.92	-1.20	-1.20
Pd-N3-Ta-N3-C	-1006.80	-987.40	Ta	-11.81	Pd	-5.21	-1.19	-1.19
Pd-N3-Ti-N3-C	-1004.90	-987.79	Ti	-7.83	Pd	-5.21	-2.04	-2.04
Pt-N3-Cd-N3-C	-997.10	-988.09	Pt	-6.08	Cd	-0.74	-1.10	-1.10
Pt-N3-Tc-N3-C	-1005.60	-987.46	Tc	-10.35	Pt	-6.08	-0.86	-0.86
Pt-N3-Zr-N3-C	-1006.58	-987.45	Pt	-6.08	Zr	-8.52	-2.27	-2.27
Pt-N3-W-N3-C	-1007.62	-987.22	W	-12.94	Pt	-6.08	-0.69	-0.69
Re-N3-Mo-N3-C	-1011.23	-987.09	Re	-12.42	Mo	-10.93	-0.39	-0.39
Re-N3-Nb-N3-C	-1010.90	-987.34	Re	-12.42	Nb	-10.21	-0.47	-0.47
Re-N3-Os-N3-C	-1011.16	-987.16	Os	-11.24	Re	-12.42	-0.17	-0.17
Re-N3-Re-N3-C	-1012.49	-986.85	Re	-12.42	Re	-12.42	-0.40	-0.40
Re-N3-Tc-N3-C	-1010.97	-987.09	Re	-12.42	Tc	-10.35	-0.56	-0.56
Rh-N3-Cd-N3-C	-998.25	-988.18	Rh	-7.25	Cd	-0.74	-1.04	-1.04
Rh-N3-Pt-N3-C	-1003.44	-987.66	Pt	-6.08	Rh	-7.25	-1.22	-1.22
Rh-N3-Rh-N3-C	-1004.59	-987.83	Rh	-7.25	Rh	-7.25	-1.13	-1.13

Table S2: Formation Energies of M_1-N_3-M_2-N_3-C

*	E_*	$E_{support}$	M_1	E_{M_1}	M_2	E_{M_2}	E_f	αE_f
Rh-N3-Ru-N3-C	-1005.72	-987.85	Rh	-7.25	Ru	-9.23	-0.69	-0.69
Rh-N3-Ti-N3-C	-1007.02	-987.77	Rh	-7.25	Ti	-7.83	-2.09	-2.09
Rh-N3-W-N3-C	-1008.85	-987.40	W	-12.94	Rh	-7.25	-0.63	-0.63
Ru-N3-Au-N3-C	-1000.65	-987.43	Ru	-9.23	Au	-3.19	-0.40	-0.40
Ru-N3-Cd-N3-C	-999.25	-988.06	Ru	-9.23	Cd	-0.74	-0.61	-0.61
Ru-N3-Pd-N3-C	-1003.60	-987.71	Ru	-9.23	Pd	-5.21	-0.72	-0.72
Ru-N3-Re-N3-C	-1009.69	-987.42	Re	-12.42	Ru	-9.23	-0.31	-0.31
Ru-N3-Zr-N3-C	-1008.80	-987.65	Ru	-9.23	Zr	-8.52	-1.70	-1.70
Ta-N3-Ni-N3-C	-1007.78	-987.65	Ta	-11.81	Ni	-5.47	-1.43	-1.43
Ta-N3-Pt-N3-C	-1007.97	-987.22	Ta	-11.81	Pt	-6.08	-1.43	-1.43
Ta-N3-Ti-N3-C	-1009.59	-987.83	Ta	-11.81	Ti	-7.83	-1.06	-1.06
Tc-N3-Zr-N3-C	-1008.95	-987.35	Tc	-10.35	Zr	-8.52	-1.36	-1.36
Ti-N3-Hf-N3-C	-1009.10	-987.91	Ti	-7.83	Hf	-9.92	-1.72	-1.72
W-N3-Au-N3-C	-1003.25	-987.14	W	-12.94	Au	-3.19	0.01	0.01
W-N3-Pd-N3-C	-1006.45	-987.40	W	-12.94	Pd	-5.21	-0.45	-0.45
W-N3-V-N3-C	-1009.74	-987.55	W	-12.94	V	-8.99	-0.13	-0.13
Zr-N3-Fe-N3-C	-1008.43	-987.85	Fe	-8.23	Zr	-8.52	-1.91	-1.91
Zr-N3-Ni-N3-C	-1006.45	-987.85	Ni	-5.47	Zr	-8.52	-2.31	-2.31
Zr-N3-Os-N3-C	-1009.84	-987.45	Os	-11.24	Zr	-8.52	-1.32	-1.32
Pd-N3-Re-N3-C	-1005.62	-987.50	Re	-12.42	Pd	-5.21	-0.25	-0.25
Zr-N3-Ti-N3-C	-1007.87	-987.97	Ti	-7.83	Zr	-8.52	-1.78	-1.78

Table S3: Formation energies of M_1-N_4-M_2-N_4-C

*	E_*	$E_{support}$	M_1	E_{M_1}	M_2	E_{M_2}	E_f	αE_f
Co-N4-Au-N4-C	-999.72	-983.28	Co	-7.03	Au	-3.19	-3.11	-2.33
Co-N4-Co-N4-C	-1006.45	-983.96	Co	-7.03	Co	-7.03	-4.22	-3.17
Co-N4-Cu-N4-C	-1001.34	-983.97	Co	-7.03	Cu	-3.73	-3.30	-2.47
Co-N4-Ni-N4-C	-1004.52	-983.87	Co	-7.03	Ni	-5.47	-4.08	-3.06
Co-N4-Os-N4-C	-1009.48	-983.63	Os	-11.24	Co	-7.03	-3.79	-2.84
Co-N4-Pd-N4-C	-1003.34	-983.80	Co	-7.03	Pd	-5.21	-3.65	-2.74
Co-N4-Pt-N4-C	-1004.47	-983.69	Co	-7.03	Pt	-6.08	-3.84	-2.88
Cr-N4-Hf-N4-C	-1012.85	-983.24	Cr	-9.50	Hf	-9.92	-5.09	-3.82
Co-N4-Rh-N4-C	-1006.15	-983.94	Co	-7.03	Rh	-7.25	-3.97	-2.98
Co-N4-Ru-N4-C	-1007.89	-983.81	Ru	-9.23	Co	-7.03	-3.91	-2.93
Cr-N4-Co-N4-C	-1009.91	-983.82	Cr	-9.50	Co	-7.03	-4.78	-3.58
Cr-N4-Cr-N4-C	-1012.13	-983.83	Cr	-9.50	Cr	-9.50	-4.65	-3.49
Cr-N4-Fe-N4-C	-1010.99	-983.84	Fe	-8.23	Cr	-9.50	-4.71	-3.53
Cr-N4-Ir-N4-C	-1010.93	-983.63	Ir	-8.84	Cr	-9.50	-4.48	-3.36
Cr-N4-Mn-N4-C	-1012.02	-983.79	Cr	-9.50	Mn	-8.94	-4.89	-3.67
Cr-N4-Ni-N4-C	-1008.03	-983.86	Ni	-5.47	Cr	-9.50	-4.60	-3.45
Cr-N4-Os-N4-C	-1012.63	-983.62	Os	-11.24	Cr	-9.50	-4.14	-3.10
Cr-N4-Rh-N4-C	-1009.59	-983.77	Rh	-7.25	Cr	-9.50	-4.53	-3.40
Cr-N4-Ru-N4-C	-1010.97	-983.80	Ru	-9.23	Cr	-9.50	-4.22	-3.17
Fe-N4-Co-N4-C	-1007.89	-983.88	Fe	-8.23	Co	-7.03	-4.38	-3.29
Fe-N4-Cu-N4-C	-1002.42	-983.98	Fe	-8.23	Cu	-3.73	-3.24	-2.43
Fe-N4-Fe-N4-C	-1009.36	-983.91	Fe	-8.23	Fe	-8.23	-4.50	-3.38
Fe-N4-Ir-N4-C	-1008.89	-983.72	Ir	-8.84	Fe	-8.23	-4.05	-3.04
Fe-N4-Ni-N4-C	-1005.66	-983.92	Fe	-8.23	Ni	-5.47	-4.02	-3.01
Fe-N4-Os-N4-C	-1010.91	-983.65	Os	-11.24	Fe	-8.23	-3.90	-2.92
Fe-N4-Pd-N4-C	-1004.59	-983.79	Fe	-8.23	Pd	-5.21	-3.68	-2.76
Fe-N4-Pt-N4-C	-1005.78	-983.68	Fe	-8.23	Pt	-6.08	-3.90	-2.92
Fe-N4-Re-N4-C	-1012.26	-983.53	Re	-12.42	Fe	-8.23	-4.04	-3.03
Fe-N4-Rh-N4-C	-1007.59	-983.85	Fe	-8.23	Rh	-7.25	-4.13	-3.10
Fe-N4-Ru-N4-C	-1009.35	-983.82	Ru	-9.23	Fe	-8.23	-4.03	-3.02

Table S3: Formation Energies of M_1-N_4-M_2-N_4-C

*	E_*	$E_{support}$	M_1	E_{M_1}	M_2	E_{M_2}	E_f	αE_f
Fe-N4-Tc-N4-C	-1010.63	-983.62	Fe	-8.23	Tc	-10.35	-4.22	-3.17
Mn-N4-Cu-N4-C	-1003.86	-983.91	Cu	-3.73	Mn	-8.94	-3.64	-2.73
Mn-N4-Fe-N4-C	-1010.63	-983.91	Fe	-8.23	Mn	-8.94	-4.78	-3.58
Mn-N4-Ir-N4-C	-1010.39	-983.71	Ir	-8.84	Mn	-8.94	-4.45	-3.34
Mn-N4-Mn-N4-C	-1011.73	-983.86	Mn	-8.94	Mn	-8.94	-4.99	-3.74
Mn-N4-Ni-N4-C	-1007.24	-983.81	Ni	-5.47	Mn	-8.94	-4.51	-3.38
Mn-N4-Os-N4-C	-1012.26	-983.61	Mn	-8.94	Os	-11.24	-4.23	-3.17
Mn-N4-Pd-N4-C	-1006.03	-983.72	Mn	-8.94	Pd	-5.21	-4.08	-3.06
Mn-N4-Rh-N4-C	-1009.09	-983.84	Rh	-7.25	Mn	-8.94	-4.53	-3.40
Mn-N4-Ru-N4-C	-1010.66	-983.76	Ru	-9.23	Mn	-8.94	-4.37	-3.28
Mn-N4-Tc-N4-C	-1011.75	-983.62	Mn	-8.94	Tc	-10.35	-4.42	-3.31
Ni-N4-Ni-N4-C	-1003.03	-983.67	Ni	-5.47	Ni	-5.47	-4.21	-3.16
Ni-N4-Os-N4-C	-1007.26	-983.69	Os	-11.24	Ni	-5.47	-3.43	-2.57
Ni-N4-Re-N4-C	-1008.66	-983.49	Re	-12.42	Ni	-5.47	-3.64	-2.73
Ni-N4-Ru-N4-C	-1005.79	-983.89	Ru	-9.23	Ni	-5.47	-3.60	-2.70
Ni-N4-Tc-N4-C	-1007.07	-983.67	Tc	-10.35	Ni	-5.47	-3.79	-2.84
Os-N4-Ir-N4-C	-1010.36	-983.35	Os	-11.24	Ir	-8.84	-3.47	-2.60
Os-N4-Os-N4-C	-1012.42	-983.29	Os	-11.24	Os	-11.24	-3.33	-2.50
Os-N4-Pt-N4-C	-1007.25	-983.30	Os	-11.24	Pt	-6.08	-3.32	-2.49
Rh-N4-Os-N4-C	-1009.12	-983.49	Rh	-7.25	Os	-11.24	-3.57	-2.68
Rh-N4-Re-N4-C	-1010.49	-983.33	Re	-12.42	Rh	-7.25	-3.74	-2.81
Rh-N4-Rh-N4-C	-1005.84	-983.83	Rh	-7.25	Rh	-7.25	-3.76	-2.82
Ru-N4-Os-N4-C	-1010.86	-983.44	Ru	-9.23	Os	-11.24	-3.47	-2.60
Ru-N4-Pt-N4-C	-1005.84	-983.55	Ru	-9.23	Pt	-6.08	-3.49	-2.62
Ru-N4-Rh-N4-C	-1007.55	-983.69	Ru	-9.23	Rh	-7.25	-3.69	-2.77
Ru-N4-Ru-N4-C	-1009.26	-983.60	Ru	-9.23	Ru	-9.23	-3.60	-2.70
Tc-N4-Rh-N4-C	-1008.87	-983.49	Tc	-10.35	Rh	-7.25	-3.89	-2.92
V-N4-Co-N4-C	-1009.60	-983.84	V	-8.99	Co	-7.03	-4.87	-3.65
V-N4-Fe-N4-C	-1010.82	-983.78	Fe	-8.23	V	-8.99	-4.91	-3.68
V-N4-Ni-N4-C	-1007.90	-983.75	V	-8.99	Ni	-5.47	-4.84	-3.63
Mn-N4-Pt-N4-C	-1007.22	-983.62	Mn	-8.94	Pt	-6.08	-4.29	-3.22
Tc-N4-Os-N4-C	-1012.14	-983.23	Os	-11.24	Tc	-10.35	-3.66	-2.75
V-N4-Rh-N4-C	-1009.31	-983.78	Rh	-7.25	V	-8.99	-4.64	-3.48
Co-N4-Tc-N4-C	-1009.24	-983.71	Tc	-10.35	Co	-7.03	-4.07	-3.05
Pd-N4-Os-N4-C	-1006.16	-983.43	Os	-11.24	Pd	-5.21	-3.14	-2.35
Cu-N4-Os-N4-C	-1004.21	-983.67	Os	-11.24	Cu	-3.73	-2.79	-2.09
Ni-N4-Ir-N4-C	-1005.02	-983.78	Ir	-8.84	Ni	-5.47	-3.46	-2.59
Cr-N4-Pt-N4-C	-1008.21	-983.60	Pt	-6.08	Cr	-9.50	-4.51	-3.38
V-N4-Ir-N4-C	-1010.63	-983.68	Ir	-8.84	V	-8.99	-4.56	-3.42
Ru-N4-Pd-N4-C	-1004.71	-983.67	Ru	-9.23	Pd	-5.21	-3.30	-2.47
Co-N4-Ir-N4-C	-1007.42	-983.79	Ir	-8.84	Co	-7.03	-3.88	-2.91
V-N4-Cu-N4-C	-1004.68	-983.77	Cu	-3.73	V	-8.99	-4.10	-3.07
Ir-N4-Ir-N4-C	-1008.27	-983.48	Ir	-8.84	Ir	-8.84	-3.56	-2.67
Ir-N4-Pt-N4-C	-1004.88	-983.37	Pt	-6.08	Ir	-8.84	-3.30	-2.47
Cr-N4-Tc-N4-C	-1011.97	-983.61	Tc	-10.35	Cr	-9.50	-4.25	-3.19
Hf-N4-Hf-N4-C	-1013.31	-983.09	Hf	-9.92	Hf	-9.92	-5.19	-3.89
Hf-N4-Ta-N4-C	-1013.99	-982.99	Ta	-11.81	Hf	-9.92	-4.63	-3.47
Mn-N4-Ta-N4-C	-1013.53	-983.42	Ta	-11.81	Mn	-8.94	-4.68	-3.51
Mn-N4-W-N4-C	-1013.94	-983.35	W	-12.94	Mn	-8.94	-4.35	-3.26
Mn-N4-Zn-N4-C	-1001.73	-983.46	Mn	-8.94	Zn	-1.11	-4.11	-3.08
Mo-N4-Hf-N4-C	-1012.72	-983.10	Mo	-10.93	Hf	-9.92	-4.39	-3.29
Mo-N4-Ir-N4-C	-1011.06	-983.13	Mo	-10.93	Ir	-8.84	-4.08	-3.06
Mo-N4-Os-N4-C	-1012.68	-983.13	Mo	-10.93	Os	-11.24	-3.69	-2.77
Mo-N4-Pd-N4-C	-1006.91	-983.12	Mo	-10.93	Pd	-5.21	-3.83	-2.87
Mo-N4-Pt-N4-C	-1008.08	-983.03	Mo	-10.93	Pt	-6.08	-4.02	-3.01
Mo-N4-Rh-N4-C	-1009.76	-983.24	Mo	-10.93	Rh	-7.25	-4.17	-3.13
Mo-N4-Ru-N4-C	-1011.11	-983.29	Mo	-10.93	Ru	-9.23	-3.83	-2.87

Table S3: Formation Energies of M_1-N_4-M_2-N_4-C

*	E_*	E_{support}	M_1	E_{M_1}	M_2	E_{M_2}	E_f	αE_f
Mo-N4-Ta-N4-C	-1013.93	-983.13	Mo	-10.93	Ta	-11.81	-4.03	-3.02
Mo-N4-Tc-N4-C	-1012.34	-983.16	Mo	-10.93	Tc	-10.35	-3.95	-2.96
Mo-N4-W-N4-C	-1014.24	-982.99	Mo	-10.93	W	-12.94	-3.69	-2.77
Nb-N4-Mo-N4-C	-1012.63	-983.17	Mo	-10.93	Nb	-10.21	-4.16	-3.12
Nb-N4-Nb-N4-C	-1012.34	-983.07	Nb	-10.21	Nb	-10.21	-4.43	-3.32
Nb-N4-Ta-N4-C	-1013.64	-983.00	Ta	-11.81	Nb	-10.21	-4.31	-3.23
Ta-N4-Ta-N4-C	-1014.86	-982.89	Ta	-11.81	Ta	-11.81	-4.17	-3.13
Tc-N4-Pd-N4-C	-1006.04	-983.35	Tc	-10.35	Pd	-5.21	-3.56	-2.67
Tc-N4-Tc-N4-C	-1011.81	-983.23	Tc	-10.35	Tc	-10.35	-3.94	-2.96
Ti-N4-Mo-N4-C	-1011.30	-983.35	Mo	-10.93	Ti	-7.83	-4.60	-3.45
Ti-N4-Nb-N4-C	-1011.08	-983.12	Nb	-10.21	Ti	-7.83	-4.96	-3.72
Ti-N4-Re-N4-C	-1012.72	-983.41	Re	-12.42	Ti	-7.83	-4.53	-3.40
Ti-N4-Ta-N4-C	-1012.52	-983.05	Ta	-11.81	Ti	-7.83	-4.91	-3.68
Ti-N4-Ti-N4-C	-1010.15	-983.41	Ti	-7.83	Ti	-7.83	-5.54	-4.16
V-N4-Mn-N4-C	-1011.68	-983.73	V	-8.99	Mn	-8.94	-5.01	-3.76
V-N4-Re-N4-C	-1013.36	-983.40	Re	-12.42	V	-8.99	-4.27	-3.20
V-N4-Tc-N4-C	-1011.74	-983.57	Tc	-10.35	V	-8.99	-4.41	-3.31
V-N4-V-N4-C	-1011.28	-983.72	V	-8.99	V	-8.99	-4.79	-3.59
W-N4-W-N4-C	-1016.08	-982.93	W	-12.94	W	-12.94	-3.63	-2.72
Zr-N4-Hf-N4-C	-1012.01	-983.17	Hf	-9.92	Zr	-8.52	-5.20	-3.90
Zr-N4-Nb-N4-C	-1011.40	-983.02	Nb	-10.21	Zr	-8.52	-4.82	-3.62
Zr-N4-Zr-N4-C	-1010.70	-983.26	Zr	-8.52	Zr	-8.52	-5.20	-3.90
W-N4-Os-N4-C	-1014.41	-982.99	W	-12.94	Os	-11.24	-3.62	-2.71
V-N4-Ru-N4-C	-1010.66	-983.78	Ru	-9.23	V	-8.99	-4.33	-3.25
V-N4-Os-N4-C	-1012.22	-983.57	Os	-11.24	V	-8.99	-4.21	-3.16
Ti-N4-Tc-N4-C	-1010.92	-983.64	Ti	-7.83	Tc	-10.35	-4.55	-3.41
Ta-N4-Os-N4-C	-1014.12	-983.11	Ta	-11.81	Os	-11.24	-3.98	-2.98
Ru-N4-W-N4-C	-1012.84	-983.15	W	-12.94	Ru	-9.23	-3.76	-2.82
Rh-N4-Ta-N4-C	-1011.28	-983.27	Ta	-11.81	Rh	-7.25	-4.48	-3.36
Nb-N4-Tc-N4-C	-1012.24	-983.29	Nb	-10.21	Tc	-10.35	-4.19	-3.14
Nb-N4-Os-N4-C	-1012.75	-983.12	Os	-11.24	Nb	-10.21	-4.09	-3.07
Mn-N4-Co-N4-C	-1009.38	-983.89	Mn	-8.94	Co	-7.03	-4.76	-3.57
Ni-N4-Rh-N4-C	-1003.87	-983.93	Rh	-7.25	Ni	-5.47	-3.61	-2.71
Mn-N4-Nb-N4-C	-1012.17	-983.44	Nb	-10.21	Mn	-8.94	-4.79	-3.59
Mn-N4-Mo-N4-C	-1012.21	-983.44	Mo	-10.93	Mn	-8.94	-4.45	-3.34
Fe-N4-Nb-N4-C	-1011.21	-983.43	Nb	-10.21	Fe	-8.23	-4.67	-3.50
Hf-N4-Os-N4-C	-1013.25	-983.29	Hf	-9.92	Os	-11.24	-4.40	-3.30
Fe-N4-Mo-N4-C	-1011.15	-983.48	Mo	-10.93	Fe	-8.23	-4.25	-3.19
Ni-N4-Pt-N4-C	-1002.85	-983.49	Pt	-6.08	Ni	-5.47	-3.90	-2.92
Ni-N4-Nb-N4-C	-1008.16	-983.43	Nb	-10.21	Ni	-5.47	-4.53	-3.40
Ni-N4-Mo-N4-C	-1007.84	-983.46	Mo	-10.93	Ni	-5.47	-3.99	-2.99
Co-N4-Nb-N4-C	-1009.93	-983.48	Nb	-10.21	Co	-7.03	-4.61	-3.46
Co-N4-Mo-N4-C	-1009.91	-983.49	Mo	-10.93	Co	-7.03	-4.23	-3.17
Cr-N4-Cu-N4-C	-1004.83	-983.88	Cu	-3.73	Cr	-9.50	-3.86	-2.90
Cr-N4-Mo-N4-C	-1012.25	-983.50	Mo	-10.93	Cr	-9.50	-4.16	-3.12
Cr-N4-Re-N4-C	-1013.58	-983.58	Re	-12.42	Cr	-9.50	-4.04	-3.03
Nb-N4-Re-N4-C	-1013.90	-983.06	Re	-12.42	Nb	-10.21	-4.10	-3.07
Nb-N4-Ir-N4-C	-1011.13	-983.16	Nb	-10.21	Ir	-8.84	-4.46	-3.34
Nb-N4-Ru-N4-C	-1011.20	-983.28	Ru	-9.23	Nb	-10.21	-4.24	-3.18
Nb-N4-Rh-N4-C	-1009.85	-983.25	Rh	-7.25	Nb	-10.21	-4.57	-3.43
Cu-N4-Mo-N4-C	-1004.85	-983.35	Mo	-10.93	Cu	-3.73	-3.42	-2.56
Nb-N4-Pt-N4-C	-1008.49	-983.01	Nb	-10.21	Pt	-6.08	-4.60	-3.45
Ru-N4-Ta-N4-C	-1012.60	-983.25	Ru	-9.23	Ta	-11.81	-4.16	-3.12
Cr-N4-Nb-N4-C	-1012.27	-983.33	Nb	-10.21	Cr	-9.50	-4.61	-3.46
Ti-N4-Co-N4-C	-1009.06	-983.81	Co	-7.03	Ti	-7.83	-5.20	-3.90
Ti-N4-Cu-N4-C	-1004.28	-983.51	Ti	-7.83	Cu	-3.73	-4.61	-3.46
Ti-N4-Ru-N4-C	-1010.17	-983.63	Ru	-9.23	Ti	-7.83	-4.74	-3.56

Table S3: Formation Energies of M_1-N_4-M_2-N_4-C

*	E_*	$E_{support}$	M_1	E_{M_1}	M_2	E_{M_2}	E_f	αE_f
Ti-N4-Fe-N4-C	-1010.26	-983.74	Fe	-8.23	Ti	-7.83	-5.23	-3.92
V-N4-Cr-N4-C	-1011.65	-983.53	V	-8.99	Cr	-9.50	-4.81	-3.61
Fe-N4-Ta-N4-C	-1012.59	-983.47	Ta	-11.81	Fe	-8.23	-4.54	-3.41
Ti-N4-Ir-N4-C	-1010.21	-983.52	Ir	-8.84	Ti	-7.83	-5.01	-3.76
Ti-N4-Os-N4-C	-1011.76	-983.48	Os	-11.24	Ti	-7.83	-4.60	-3.45
Tc-N4-Ta-N4-C	-1013.46	-983.21	Ta	-11.81	Tc	-10.35	-4.05	-3.04
V-N4-Mo-N4-C	-1012.08	-983.49	Mo	-10.93	V	-8.99	-4.33	-3.25
Ti-N4-Pt-N4-C	-1007.74	-983.23	Ti	-7.83	Pt	-6.08	-5.30	-3.97
Co-N4-Ta-N4-C	-1011.37	-983.54	Ta	-11.81	Co	-7.03	-4.49	-3.37
Co-N4-W-N4-C	-1011.68	-983.38	Co	-7.03	W	-12.94	-4.17	-3.13
Cr-N4-W-N4-C	-1013.89	-983.39	W	-12.94	Cr	-9.50	-4.03	-3.02
Co-N4-Hf-N4-C	-1010.57	-983.66	Co	-7.03	Hf	-9.92	-4.98	-3.74
V-N4-Pt-N4-C	-1008.10	-983.48	Pt	-6.08	V	-8.99	-4.78	-3.58
Cr-N4-Pd-N4-C	-1006.99	-983.70	Pd	-5.21	Cr	-9.50	-4.29	-3.22
Cu-N4-Nb-N4-C	-1005.13	-983.32	Nb	-10.21	Cu	-3.73	-3.94	-2.96
Mn-N4-Re-N4-C	-1013.46	-983.51	Re	-12.42	Mn	-8.94	-4.30	-3.22
W-N4-Re-N4-C	-1015.43	-983.01	W	-12.94	Re	-12.42	-3.53	-2.65
Tc-N4-Re-N4-C	-1013.43	-983.13	Tc	-10.35	Re	-12.42	-3.77	-2.83
Cu-N4-Re-N4-C	-1005.61	-983.48	Re	-12.42	Cu	-3.73	-2.99	-2.24
Ru-N4-Re-N4-C	-1012.24	-983.29	Re	-12.42	Ru	-9.23	-3.65	-2.74
Pd-N4-Re-N4-C	-1007.54	-983.21	Re	-12.42	Pd	-5.21	-3.35	-2.51
Mo-N4-Mo-N4-C	-1012.54	-983.10	Mo	-10.93	Mo	-10.93	-3.79	-2.84
Fe-N4-W-N4-C	-1012.94	-983.36	W	-12.94	Fe	-8.23	-4.21	-3.16
Rh-N4-Ir-N4-C	-1007.08	-983.65	Ir	-8.84	Rh	-7.25	-3.67	-2.75
Tc-N4-Ir-N4-C	-1010.13	-983.32	Tc	-10.35	Ir	-8.84	-3.81	-2.86
Ni-N4-Cu-N4-C	-999.89	-983.70	Cu	-3.73	Ni	-5.47	-3.50	-2.62
Co-N4-Re-N4-C	-1010.82	-983.52	Re	-12.42	Co	-7.03	-3.93	-2.95
Cu-N4-W-N4-C	-1006.58	-983.16	W	-12.94	Cu	-3.73	-3.37	-2.53
Re-N4-Re-N4-C	-1015.00	-983.07	Re	-12.42	Re	-12.42	-3.55	-2.66
Ta-N4-Ir-N4-C	-1012.54	-983.18	Ir	-8.84	Ta	-11.81	-4.36	-3.27
Cr-N4-Ta-N4-C	-1013.54	-983.08	Cr	-9.50	Ta	-11.81	-4.58	-3.44
Pd-N4-Pt-N4-C	-1001.34	-983.37	Pt	-6.08	Pd	-5.21	-3.34	-2.50
Cu-N4-Ru-N4-C	-1002.69	-983.90	Ru	-9.23	Cu	-3.73	-2.91	-2.18
Ru-N4-Ir-N4-C	-1008.83	-983.54	Ru	-9.23	Ir	-8.84	-3.61	-2.71
Ni-N4-Pd-N4-C	-1001.70	-983.65	Ni	-5.47	Pd	-5.21	-3.68	-2.76
Ni-N4-W-N4-C	-1009.54	-983.28	W	-12.94	Ni	-5.47	-3.93	-2.95
Mn-N4-Hf-N4-C	-1012.58	-983.55	Mn	-8.94	Hf	-9.92	-5.08	-3.81
Zr-N4-Au-N4-C	-1002.82	-982.90	Zr	-8.52	Au	-3.19	-4.11	-3.08

Table S4: Structural stability (αE_f), metal-nitrogen bond lengths ($L_{M_1\text{-}N}$, $L_{M_2\text{-}N}$), intermetallic distance $L_{M_1\text{-}M_2}$, and spin-resolved ICOHP values of M_1–N and M_2–N bonds ($I^{\uparrow}_{M_1\text{-}N}$, $I^{\downarrow}_{M_1\text{-}N}$, $I^{\uparrow}_{M_2\text{-}N}$, $I^{\downarrow}_{M_2\text{-}N}$), as well as spin-up/spin-down occupied and unoccupied $d_{x^2-y^2}$ orbital populations at the M_1 and M_2 sites ($e^{\uparrow}_{occ}(M_1)$, $e^{\downarrow}_{occ}(M_1)$, $e^{\uparrow}_{occ}(M_2)$, $e^{\downarrow}_{occ}(M_2)$, $e^{\uparrow}_{unocc}(M_1)$, $e^{\downarrow}_{unocc}(M_1)$, $e^{\uparrow}_{unocc}(M_2)$, $e^{\downarrow}_{unocc}(M_2)$) for M_1–N_3–M_2–N_3–C

*	αE_f	$L_{M_1\text{-}N}$	$I^{\uparrow}_{M_1\text{-}N}$	$I^{\downarrow}_{M_1\text{-}N}$	$L_{M_2\text{-}N}$	$I^{\uparrow}_{M_2\text{-}N}$	$I^{\downarrow}_{M_2\text{-}N}$	$L_{M_1\text{-}M_2}$	$e^{\uparrow}_{occ}(M_1)$	$e^{\downarrow}_{occ}(M_1)$	$e^{\uparrow}_{occ}(M_2)$	$e^{\downarrow}_{occ}(M_2)$	$e^{\uparrow}_{unocc}(M_1)$	$e^{\downarrow}_{unocc}(M_1)$	$e^{\uparrow}_{unocc}(M_2)$	$e^{\downarrow}_{unocc}(M_2)$
Ti-N3-Ti-N3-C	-2.33	2.00	-2.18	-2.18	2.00	-2.19	-2.18	2.50	0.18	0.16	0.18	0.15	0.76	0.78	0.76	0.78
Ti-N3-V-N3-C	-1.96	1.98	-2.23	-2.29	1.93	-2.04	-1.98	2.41	0.16	0.17	0.25	0.18	0.77	0.77	0.73	0.79
Ti-N3-Cr-N3-C	-1.88	2.00	-2.17	-2.18	1.96	-2.03	-2.07	2.52	0.17	0.17	0.50	0.19	0.77	0.77	0.52	0.80
Ti-N3-Mn-N3-C	-2.08	2.01	-2.11	-2.17	1.97	-1.66	-1.82	2.40	0.15	0.18	0.77	0.18	0.78	0.76	0.23	0.80
Fe-N3-Ti-N3-C	-1.93	1.93	-1.69	-1.86	2.04	-2.01	-2.02	2.22	0.78	0.31	0.15	0.19	0.26	0.71	0.78	0.75
Ti-N3-Co-N3-C	-2.15	2.02	-2.08	-2.08	1.92	-1.76	-1.76	2.04	0.19	0.19	0.65	0.65	0.75	0.74	0.39	0.38
Ni-N3-Ti-N3-C	-2.27	1.89	-1.78	-1.73	2.03	-2.11	-2.08	2.25	0.72	0.78	0.18	0.16	0.33	0.26	0.77	0.77
Ti-N3-Cu-N3-C	-1.93	1.98	-2.44	-2.36	1.95	-1.30	-1.30	2.54	0.19	0.16	0.89	0.88	0.75	0.79	0.13	0.14
Ti-N3-Zn-N3-C	-1.95	2.01	-2.11	-2.05	2.12	-1.31	-1.29	2.61	0.20	0.17	0.98	0.98	0.75	0.77	0.04	0.04
V-N3-V-N3-C	-1.44	1.91	-2.16	-2.04	1.91	-2.16	-2.04	2.38	0.34	0.19	0.34	0.19	0.66	0.79	0.66	0.79
V-N3-Cr-N3-C	-1.43	1.98	-2.04	-1.98	1.95	-1.94	-2.14	2.63	0.37	0.16	0.54	0.18	0.61	0.81	0.48	0.82
V-N3-Mn-N3-C	-1.32	1.96	-2.03	-2.03	1.91	-1.60	-2.24	2.61	0.45	0.15	0.79	0.17	0.53	0.82	0.22	0.82
Fe-N3-V-N3-C	-1.66	1.95	-1.86	-1.68	2.00	-1.80	-1.75	2.04	0.39	0.77	0.34	0.19	0.64	0.27	0.65	0.78
V-N3-Co-N3-C	-1.66	1.98	-1.92	-1.85	1.90	-1.92	-1.75	2.11	0.28	0.20	0.49	0.78	0.71	0.78	0.55	0.26
Ni-N3-V-N3-C	-1.76	1.87	-1.90	-1.76	1.93	-2.11	-2.00	2.23	0.60	0.84	0.26	0.20	0.45	0.20	0.74	0.78
V-N3-Cu-N3-C	-1.60	1.99	-2.06	-1.92	1.94	-1.32	-1.29	2.47	0.39	0.17	0.90	0.90	0.60	0.80	0.12	0.13
V-N3-Zn-N3-C	-1.56	1.96	-2.01	-1.92	2.07	-1.43	-1.40	2.57	0.26	0.18	0.98	0.98	0.72	0.80	0.04	0.04
Cr-N3-Cr-N3-C	-1.37	1.93	-1.85	-2.15	1.93	-1.85	-2.15	2.51	0.64	0.17	0.64	0.17	0.39	0.83	0.39	0.83
Cr-N3-Mn-N3-C	-1.50	1.94	-1.99	-2.13	1.90	-1.66	-2.26	2.55	0.55	0.18	0.76	0.18	0.47	0.82	0.26	0.81
Fe-N3-Cr-N3-C	-1.12	1.83	-2.23	-1.91	1.93	-2.07	-2.05	2.34	0.28	0.82	0.47	0.19	0.76	0.23	0.55	0.80
Cr-N3-Co-N3-C	-1.48	1.93	-2.06	-2.03	1.87	-2.01	-1.81	2.24	0.41	0.20	0.34	0.81	0.61	0.80	0.70	0.23
Ni-N3-Cr-N3-C	-1.70	1.89	-1.81	-1.71	1.95	-1.99	-1.95	2.35	0.56	0.85	0.46	0.19	0.46	0.20	0.54	0.81
Cr-N3-Cu-N3-C	-1.76	1.94	-2.03	-1.97	1.93	-1.37	-1.32	2.42	0.49	0.19	0.88	0.90	0.53	0.81	0.14	0.13
Cr-N3-Zn-N3-C	-1.62	1.93	-2.04	-2.02	2.02	-1.56	-1.53	2.60	0.36	0.20	0.99	0.99	0.66	0.80	0.04	0.04
Mn-N3-Mn-N3-C	-1.56	1.94	-1.90	-1.81	1.95	-1.88	-1.78	2.43	0.20	0.67	0.21	0.70	0.80	0.34	0.79	0.31
Fe-N3-Mn-N3-C	-1.55	1.91	-1.90	-1.91	1.96	-1.84	-1.75	2.31	0.59	0.41	0.20	0.66	0.46	0.62	0.79	0.34
Mn-N3-Co-N3-C	-1.56	1.94	-1.84	-1.71	1.89	-1.82	-1.92	2.29	0.19	0.73	0.69	0.53	0.80	0.27	0.35	0.50
Ni-N3-Mn-N3-C	-1.95	1.91	-1.75	-1.74	1.93	-1.75	-1.89	2.28	0.71	0.75	0.72	0.20	0.34	0.30	0.29	0.79
Mn-N3-Cu-N3-C	-1.72	1.92	-1.95	-2.20	1.94	-1.42	-1.35	2.42	0.59	0.22	0.85	0.88	0.43	0.78	0.18	0.15
Mn-N3-Zn-N3-C	-1.73	1.96	-1.48	-2.05	1.99	-1.51	-1.73	2.54	0.83	0.16	1.01	0.99	0.18	0.82	0.05	0.06
Fe-N3-Fe-N3-C	-1.02	1.88	-1.89	-2.05	1.88	-2.04	-1.91	2.10	0.61	0.29	0.29	0.60	0.44	0.74	0.75	0.44
Fe-N3-Co-N3-C	-1.47	1.91	-1.72	-1.90	1.91	-1.73	-1.88	2.17	0.77	0.28	0.73	0.47	0.27	0.75	0.31	0.57
Fe-N3-Ni-N3-C	-1.56	1.92	-1.76	-1.95	1.90	-1.75	-1.76	2.30	0.75	0.27	0.72	0.68	0.30	0.76	0.33	0.36
Fe-N3-Cu-N3-C	-1.45	1.89	-1.94	-2.07	1.95	-1.37	-1.32	2.41	0.59	0.43	0.85	0.90	0.46	0.62	0.18	0.12
Fe-N3-Zn-N3-C	-1.41	1.95	-1.58	-1.82	1.99	-1.47	-1.61	2.47	0.87	0.23	1.01	1.00	0.17	0.79	0.04	0.05
Co-N3-Co-N3-C	-1.35	1.89	-1.94	-1.77	1.89	-1.94	-1.77	2.24	0.36	0.75	0.36	0.75	0.68	0.29	0.68	0.29
Ni-N3-Co-N3-C	-1.56	1.87	-1.78	-1.79	1.91	-1.74	-1.88	2.33	0.71	0.76	0.72	0.56	0.34	0.27	0.32	0.47
Co-N3-Cu-N3-C	-1.49	1.85	-1.98	-2.08	1.97	-1.30	-1.28	2.42	0.56	0.51	0.87	0.89	0.49	0.53	0.16	0.13
Co-N3-Zn-N3-C	-1.46	1.83	-2.01	-2.06	1.91	-1.80	-1.81	2.48	0.48	0.44	1.00	1.00	0.57	0.60	0.06	0.06
Ni-N3-Ni-N3-C	-1.88	1.88	-1.76	-1.76	1.88	-1.76	-1.76	2.36	0.72	0.72	0.72	0.72	0.34	0.33	0.33	0.33
Ni-N3-Cu-N3-C	-1.68	1.89	-1.76	-1.85	1.97	-1.33	-1.34	2.40	0.70	0.59	0.86	0.86	0.33	0.46	0.15	0.17
Ni-N3-Zn-N3-C	-1.78	1.88	-1.64	-1.87	1.93	-1.69	-1.78	2.45	0.91	0.42	1.01	1.00	0.14	0.62	0.04	0.06
Cu-N3-Cu-N3-C	-1.51	1.93	-1.39	-1.53	1.93	-1.39	-1.53	2.39	0.96	0.78	0.96	0.78	0.07	0.22	0.07	0.22
Cu-N3-Zn-N3-C	-1.86	1.92	-1.48	-1.48	1.96	-1.65	-1.65	2.39	0.90	0.90	1.00	1.00	0.12	0.12	1.01	1.01
Zn-N3-Zn-N3-C	-1.98	1.96	-1.75	-1.75	1.96	-1.75	-1.75	2.54	1.00	1.00	1.00	1.00	0.07	0.07	0.07	0.07

Table S5: Structural stability (αE_f), metal-nitrogen bond lengths ($L_{M_1\text{-}N}$, $L_{M_2\text{-}N}$), intermetallic distance $L_{M_1\text{-}M_2}$, and spin-resolved ICOHP values of M_1–N and M_2–N bonds ($I^{\uparrow}_{M_1\text{-}N}$, $I^{\downarrow}_{M_1\text{-}N}$, $I^{\uparrow}_{M_2\text{-}N}$, $I^{\downarrow}_{M_2\text{-}N}$), as well as spin-up/spin-down occupied and unoccupied $d_{x^2-y^2}$ orbital populations at the M_1 and M_2 sites ($e^{\uparrow}_{occ}(M_1)$, $e^{\downarrow}_{occ}(M_1)$, $e^{\uparrow}_{occ}(M_2)$, $e^{\downarrow}_{occ}(M_2)$, $e^{\uparrow}_{unocc}(M_1)$, $e^{\downarrow}_{unocc}(M_1)$, $e^{\uparrow}_{unocc}(M_2)$, $e^{\downarrow}_{unocc}(M_2)$) for M_1–N_4–M_2–N_4–C

*	αE_f	$L_{M_1\text{-}N}$	$I^{\uparrow}_{M_1\text{-}N}$	$I^{\downarrow}_{M_1\text{-}N}$	$L_{M_2\text{-}N}$	$I^{\uparrow}_{M_2\text{-}N}$	$I^{\downarrow}_{M_2\text{-}N}$	$L_{M_1\text{-}M_2}$	$e^{\uparrow}_{occ}(M_1)$	$e^{\downarrow}_{occ}(M_1)$	$e^{\uparrow}_{occ}(M_2)$	$e^{\downarrow}_{occ}(M_2)$	$e^{\uparrow}_{unocc}(M_1)$	$e^{\downarrow}_{unocc}(M_1)$	$e^{\uparrow}_{unocc}(M_2)$	$e^{\downarrow}_{unocc}(M_2)$
Ti-N4-Ti-N4-C	-4.15	1.94	-2.60	-2.55	1.94	-2.60	-2.55	2.56	0.31	0.21	0.31	0.21	0.65	0.74	0.65	0.75
Ti-N4-V-N4-C	-3.95	1.93	-2.93	-2.91	1.90	-2.24	-2.23	2.47	0.31	0.22	0.42	0.26	0.65	0.74	0.60	0.75
Ti-N4-Cr-N4-C	-3.94	1.88	-3.08	-3.16	1.91	-2.11	-2.18	2.51	0.28	0.24	0.53	0.24	0.68	0.72	0.51	0.78
Ti-N4-Mn-N4-C	-3.98	1.88	-3.06	-3.00	1.87	-1.92	-2.11	2.38	0.26	0.30	0.54	0.38	0.70	0.66	0.49	0.63
Ti-N4-Fe-N4-C	-3.92	1.85	-3.18	-3.12	1.86	-1.86	-2.05	2.35	0.27	0.29	0.59	0.50	0.70	0.67	0.48	0.56
Ti-N4-Co-N4-C	-3.90	1.84	-3.27	-3.24	1.85	-1.86	-1.92	2.35	0.28	0.28	0.59	0.57	0.69	0.68	0.48	0.49
Ti-N4-Ni-N4-C	-3.96	1.83	-3.40	-3.40	1.88	-1.79	-1.79	2.44	0.27	0.27	0.66	0.66	0.71	0.70	0.41	0.41
Ti-N4-Cu-N4-C	-3.46	1.84	-3.42	-3.48	1.92	-1.20	-1.56	2.47	0.26	0.27	0.99	0.71	0.71	0.70	0.04	0.32
Ti-N4-Zn-N4-C	-3.66	1.86	-3.26	-3.26	1.97	-1.52	-1.52	2.52	0.26	0.26	1.02	1.02	0.71	0.70	0.03	0.03
V-N4-V-N4-C	-3.59	1.86	-2.62	-2.63	1.86	-2.60	-2.61	2.27	0.38	0.36	0.38	0.36	0.63	0.63	0.63	0.63
V-N4-Cr-N4-C	-3.61	1.83	-2.74	-2.72	1.90	-2.45	-2.51	2.44	0.39	0.28	0.52	0.26	0.63	0.72	0.53	0.77
V-N4-Mn-N4-C	-3.76	1.83	-2.79	-2.67	1.86	-2.32	-2.47	2.31	0.36	0.37	0.52	0.41	0.66	0.64	0.51	0.61
V-N4-Fe-N4-C	-3.64	1.79	-2.89	-2.85	1.85	-2.33	-2.35	2.26	0.39	0.36	0.53	0.54	0.62	0.65	0.53	0.52
V-N4-Co-N4-C	-3.65	1.79	-2.99	-2.98	1.84	-2.25	-2.18	2.29	0.38	0.34	0.57	0.59	0.64	0.66	0.50	0.48
V-N4-Ni-N4-C	-3.63	1.78	-3.14	-3.09	1.88	-2.01	-1.98	2.35	0.37	0.33	0.66	0.66	0.66	0.69	0.42	0.42
V-N4-Cu-N4-C	-3.07	1.78	-3.20	-3.09	1.93	-1.80	-1.40	2.36	0.37	0.32	0.69	1.00	0.66	0.70	0.34	0.03
V-N4-Zn-N4-C	-3.31	1.81	-3.02	-2.96	1.99	-1.67	-1.67	2.42	0.36	0.31	1.02	1.02	0.66	0.70	0.03	0.03
Cr-N4-Cr-N4-C	-3.49	1.83	-2.61	-2.63	1.83	-2.61	-2.63	2.27	0.46	0.41	0.46	0.41	0.59	0.63	0.59	0.63
Cr-N4-Mn-N4-C	-3.67	1.80	-2.95	-2.94	1.84	-2.18	-2.27	2.28	0.45	0.41	0.51	0.42	0.61	0.64	0.53	0.60
Cr-N4-Fe-N4-C	-3.58	1.79	-2.92	-3.03	1.83	-2.22	-2.12	2.29	0.47	0.37	0.52	0.54	0.59	0.66	0.56	0.53
Cr-N4-Co-N4-C	-3.58	1.77	-3.24	-3.05	1.82	-1.94	-2.13	2.29	0.37	0.46	0.59	0.56	0.68	0.59	0.49	0.51
Cr-N4-Ni-N4-C	-3.45	1.76	-3.15	-3.31	1.87	-1.81	-1.75	2.33	0.45	0.36	0.65	0.65	0.61	0.69	0.43	0.42
Cr-N4-Cu-N4-C	-2.89	1.77	-3.23	-3.36	1.93	-1.56	-1.16	2.34	0.45	0.35	0.69	1.00	0.61	0.70	0.34	0.03
Cr-N4-Zn-N4-C	-2.99	1.77	-3.13	-3.31	1.97	-1.56	-1.57	2.38	0.45	0.36	1.03	1.03	0.61	0.69	0.03	0.03
Mn-N4-Mn-N4-C	-3.75	1.82	-2.40	-2.52	1.82	-2.40	-2.52	2.28	0.50	0.41	0.50	0.41	0.53	0.61	0.53	0.61
Mn-N4-Fe-N4-C	-3.61	1.79	-2.41	-2.76	1.79	-2.62	-2.27	2.28	0.53	0.39	0.48	0.58	0.52	0.64	0.60	0.51
Mn-N4-Co-N4-C	-3.57	1.77	-2.58	-2.84	1.80	-2.35	-2.19	2.27	0.51	0.39	0.56	0.58	0.53	0.64	0.53	0.50
Mn-N4-Ni-N4-C	-3.38	1.82	-2.22	-2.68	1.85	-1.94	-1.94	2.48	0.58	0.33	0.65	0.65	0.46	0.69	0.43	0.43
Mn-N4-Cu-N4-C	-2.73	1.77	-2.73	-3.05	1.91	-1.48	-1.46	2.33	0.49	0.38	0.82	0.80	0.55	0.66	0.16	0.18
Mn-N4-Zn-N4-C	-3.08	1.86	-2.05	-2.48	1.95	-1.67	-1.63	2.52	0.61	0.31	1.03	1.02	0.42	0.70	0.03	0.03
Fe-N4-Fe-N4-C	-3.37	1.78	-2.47	-2.56	1.78	-2.47	-2.56	2.24	0.55	0.50	0.55	0.50	0.54	0.58	0.54	0.58
Fe-N4-Co-N4-C	-3.28	1.76	-2.53	-2.75	1.78	-2.37	-2.30	2.27	0.55	0.49	0.55	0.56	0.53	0.59	0.56	0.57
Fe-N4-Ni-N4-C	-3.09	1.79	-2.16	-2.70	1.84	-1.92	-1.98	2.45	0.63	0.43	0.66	0.64	0.46	0.64	0.43	0.43
Fe-N4-Cu-N4-C	-2.47	1.81	-2.11	-2.72	1.89	-1.30	-1.72	2.47	0.64	0.42	1.00	0.67	0.44	0.65	0.03	0.36
Fe-N4-Zn-N4-C	-2.76	1.83	-2.43	-2.26	1.94	-1.58	-1.63	2.47	0.47	0.58	1.02	1.03	0.61	0.49	0.04	0.03
Co-N4-Co-N4-C	-3.16	1.77	-2.42	-2.42	1.77	-2.42	-2.42	2.26	0.56	0.56	0.56	0.56	0.53	0.52	0.53	0.52
Co-N4-Ni-N4-C	-3.06	1.78	-2.14	-2.56	1.83	-1.94	-2.06	2.44	0.63	0.51	0.66	0.64	0.46	0.57	0.43	0.44
Co-N4-Cu-N4-C	-2.48	1.79	-2.59	-2.16	1.88	-1.37	-1.74	2.44	0.50	0.63	0.95	0.68	0.58	0.45	0.07	0.36
Co-N4-Zn-N4-C	-2.64	1.80	-2.03	-2.47	1.93	-1.64	-1.62	2.46	0.65	0.51	1.03	1.02	0.43	0.56	0.03	0.03
Ni-N4-Ni-N4-C	-3.16	1.82	-2.00	-2.00	1.82	-2.00	-2.00	2.59	0.65	0.65	0.65	0.65	0.44	0.43	0.44	0.43
Ni-N4-Cu-N4-C	-2.62	1.84	-1.97	-1.97	1.87	-1.66	-1.66	2.60	0.64	0.64	0.78	0.78	0.43	0.43	0.24	0.24
Ni-N4-Zn-N4-C	-2.74	1.85	-1.88	-1.88	1.90	-1.67	-1.67	2.61	0.68	0.68	1.04	1.04	0.41	0.40	0.03	0.02
Cu-N4-Cu-N4-C	-2.17	1.88	-1.51	-1.77	1.88	-1.51	-1.77	2.65	0.91	0.69	0.91	0.69	0.13	0.34	0.13	0.34
Cu-N4-Zn-N4-C	-2.36	1.87	-1.61	-1.61	1.91	-1.72	-1.72	2.66	0.80	0.80	1.02	1.02	0.24	0.24	0.05	0.04
Zn-N4-Zn-N4-C	-2.44	1.92	-1.71	-1.71	1.92	-1.71	-1.71	2.57	1.01	1.01	1.01	1.01	0.05	0.05	0.05	0.05

S8 Hydrogen adsorption energies on M-N_4-C, and $M_1M_2N_6$.

Table S6: Hydrogen adsorption energies of M-N_4-C

*	E_*	E_{H^*}	G_{corr}	G_{H^*}	ΔG_{H^*}
Zn-N4-C	-1014.54	-1017.05	0.15	-1016.90	1.04
Ag-N4-C	-1013.37	-1016.03	0.13	-1015.90	0.88
Au-N4-C	-1014.43	-1015.80	0.09	-1015.72	2.12
Co-N4-C	-1020.64	-1024.06	0.20	-1023.86	0.19
Cr-N4-C	-1023.27	-1026.60	0.15	-1026.44	0.24
Cu-N4-C	-1016.52	-1018.26	0.13	-1018.13	1.80
Fe-N4-C	-1021.71	-1024.98	0.18	-1024.80	0.31
Mn-N4-C	-1022.84	-1025.97	0.16	-1025.80	0.45
Mo-N4-C	-1021.93	-1026.04	0.16	-1025.88	-0.54
Hf-N4-C	-1023.60	-1028.36	0.15	-1028.22	-1.21
Nb-N4-C	-1022.31	-1026.69	0.16	-1026.54	-0.82
Re-N4-C	-1022.64	-1026.99	0.17	-1026.82	-0.77
Ni-N4-C	-1019.40	-1021.21	0.14	-1021.08	1.72
Os-N4-C	-1021.97	-1026.22	0.17	-1026.06	-0.69
Pd-N4-C	-1018.40	-1020.04	0.12	-1019.92	1.89
Ir-N4-C	-1021.07	-1025.05	0.20	-1024.85	-0.37
Rh-N4-C	-1019.89	-1023.82	0.20	-1023.62	-0.32
Ru-N4-C	-1020.71	-1024.84	0.19	-1024.65	-0.54
Ta-N4-C	-1023.69	-1028.38	0.17	-1028.22	-1.12
Tc-N4-C	-1021.57	-1025.54	0.18	-1025.36	-0.38
Ti-N4-C	-1021.86	-1026.01	0.14	-1025.87	-0.60
W-N4-C	-1023.33	-1027.34	0.17	-1027.17	-0.43
Zr-N4-C	-1022.18	-1026.69	0.13	-1026.56	-0.97
Pt-N4-C	-1019.54	-1021.53	0.16	-1021.37	1.58
V-N4-C	-1022.41	-1026.17	0.14	-1026.02	-0.20
Cd-N4-C	-1012.54	-1015.91	0.13	-1015.78	0.17

Table S7: Hydrogen adsorption energies of M_1-N_3-M_2-N_3-C

*	E_*	E_{H^*}	G_{corr}	G_{H^*}	ΔG_{H^*}
Co-N3-Cu-N3-C	-1001.86	-1005.44	0.19	-1005.25	0.02
Co-N3-Co-N3-C	-1004.87	-1008.92	0.22	-1008.70	-0.42
Cr-N3-Co-N3-C	-1007.32	-1011.66	0.22	-1011.44	-0.71
Cr-N3-Cr-N3-C	-1009.72	-1014.16	0.19	-1013.97	-0.84
Cr-N3-Cu-N3-C	-1004.82	-1008.25	0.18	-1008.07	0.16
Cr-N3-Mn-N3-C	-1009.37	-1013.63	0.19	-1013.43	-0.65
Cu-N3-Cu-N3-C	-998.69	-1001.64	0.22	-1001.42	0.68
Cu-N3-Zn-N3-C	-996.64	-1000.07	0.19	-999.88	0.17
Fe-N3-Co-N3-C	-1006.26	-1010.04	0.23	-1009.81	-0.14
Fe-N3-Cr-N3-C	-1008.30	-1012.60	0.21	-1012.39	-0.68
Fe-N3-Cu-N3-C	-1002.88	-1006.43	0.23	-1006.20	0.08
Fe-N3-Fe-N3-C	-1007.17	-1011.19	0.28	-1010.92	-0.34
Fe-N3-Mn-N3-C	-1008.04	-1012.27	0.22	-1012.05	-0.60
Fe-N3-Ni-N3-C	-1004.92	-1008.65	0.24	-1008.41	-0.08
Fe-N3-Ti-N3-C	-1007.91	-1012.11	0.23	-1011.88	-0.57
Fe-N3-V-N3-C	-1007.86	-1012.39	0.22	-1012.17	-0.90
Fe-N3-Zn-N3-C	-1000.21	-1004.35	0.19	-1004.15	-0.54
Ir-N3-Co-N3-C	-1005.66	-1009.98	0.21	-1009.77	-0.70
Ir-N3-Cr-N3-C	-1008.35	-1012.91	0.22	-1012.69	-0.94
Ir-N3-Cu-N3-C	-1002.72	-1006.62	0.19	-1006.43	-0.30
Ir-N3-Fe-N3-C	-1007.05	-1011.20	0.21	-1010.98	-0.52

Table S7: Hydrogen adsorption energies of M_1-N_3-M_2-N_3-C

$*$	E_*	E_{H^*}	G_{corr}	G_{H^*}	ΔG_{H^*}
Ir-N3-Mn-N3-C	-1008.17	-1012.62	0.22	-1012.40	-0.82
Ir-N3-Mo-N3-C	-1008.61	-1012.77	0.17	-1012.60	-0.58
Ir-N3-Ni-N3-C	-1004.46	-1008.80	0.22	-1008.59	-0.72
Ir-N3-Pt-N3-C	-1004.51	-1008.81	0.22	-1008.58	-0.66
Ir-N3-Ru-N3-C	-1006.81	-1010.58	0.34	-1010.23	-0.01
Ir-N3-V-N3-C	-1008.26	-1012.50	0.22	-1012.28	-0.61
Mn-N3-Co-N3-C	-1007.14	-1011.33	0.21	-1011.11	-0.56
Mn-N3-Cu-N3-C	-1004.07	-1007.70	0.18	-1007.52	-0.04
Mn-N3-Mn-N3-C	-1008.96	-1013.19	0.20	-1012.99	-0.62
Mn-N3-Zn-N3-C	-1001.41	-1005.55	0.18	-1005.37	-0.56
Mo-N3-Co-N3-C	-1007.61	-1011.66	0.17	-1011.49	-0.47
Mo-N3-Cr-N3-C	-1009.92	-1013.93	0.17	-1013.76	-0.44
Mo-N3-Cu-N3-C	-1003.82	-1007.84	0.14	-1007.70	-0.47
Mo-N3-Fe-N3-C	-1008.73	-1012.65	0.15	-1012.49	-0.35
Mo-N3-Mn-N3-C	-1009.67	-1013.67	0.14	-1013.53	-0.45
Mo-N3-Mo-N3-C	-1010.04	-1014.32	0.19	-1014.13	-0.68
Mo-N3-Ni-N3-C	-1006.04	-1010.09	0.16	-1009.93	-0.48
Mo-N3-V-N3-C	-1008.55	-1013.20	0.19	-1013.01	-1.06
Ni-N3-Co-N3-C	-1003.71	-1007.76	0.22	-1007.54	-0.42
Ni-N3-Cr-N3-C	-1006.47	-1010.41	0.22	-1010.19	-0.31
Ni-N3-Cu-N3-C	-1000.70	-1004.08	0.22	-1003.87	0.24
Ni-N3-Mn-N3-C	-1006.35	-1009.90	0.20	-1009.70	0.06
Ni-N3-Ni-N3-C	-1002.85	-1006.43	0.22	-1006.20	0.05
Ni-N3-Ti-N3-C	-1005.82	-1009.39	0.17	-1009.22	0.01
Ni-N3-V-N3-C	-1006.06	-1009.71	0.20	-1009.51	-0.04
Ni-N3-Zn-N3-C	-998.23	-1001.99	0.21	-1001.78	-0.14
Pt-N3-Co-N3-C	-1003.84	-1008.01	0.23	-1007.78	-0.53
Pt-N3-Cr-N3-C	-1006.56	-1010.80	0.24	-1010.56	-0.59
Pt-N3-Cu-N3-C	-1000.99	-1004.53	0.22	-1004.31	0.09
Pt-N3-Fe-N3-C	-1005.08	-1009.00	0.22	-1008.78	-0.29
Pt-N3-Mn-N3-C	-1006.48	-1010.20	0.23	-1009.97	-0.08
Pt-N3-Mo-N3-C	-1006.36	-1010.46	0.17	-1010.30	-0.53
Pt-N3-Ni-N3-C	-1002.97	-1006.80	0.23	-1006.57	-0.19
Pt-N3-Pt-N3-C	-1002.81	-1006.95	0.24	-1006.71	-0.49
Pt-N3-Ru-N3-C	-1004.66	-1008.43	0.19	-1008.24	-0.17
Pt-N3-Ti-N3-C	-1005.99	-1009.70	0.23	-1009.47	-0.07
Pt-N3-V-N3-C	-1006.06	-1010.12	0.23	-1009.89	-0.42
Pt-N3-Zn-N3-C	-998.11	-1002.44	0.23	-1002.21	-0.69
Ru-N3-Co-N3-C	-1005.81	-1009.72	0.20	-1009.52	-0.30
Ru-N3-Cr-N3-C	-1008.57	-1012.00	0.15	-1011.84	0.14
Ru-N3-Cu-N3-C	-1002.48	-1006.68	0.20	-1006.49	-0.60
Ru-N3-Fe-N3-C	-1007.22	-1011.17	0.19	-1010.98	-0.35
Ru-N3-Mn-N3-C	-1008.15	-1011.46	0.18	-1011.28	0.28
Ru-N3-Mo-N3-C	-1009.03	-1012.82	0.16	-1012.66	-0.22
Ru-N3-Ni-N3-C	-1004.44	-1008.54	0.20	-1008.34	-0.50
Ru-N3-Ru-N3-C	-1007.20	-1010.94	0.20	-1010.74	-0.13
Ru-N3-Ti-N3-C	-1008.24	-1012.56	0.29	-1012.27	-0.62
Ru-N3-V-N3-C	-1008.44	-1012.14	0.15	-1011.99	-0.14
Ti-N3-Co-N3-C	-1007.14	-1010.84	0.17	-1010.66	-0.11
Ti-N3-Cr-N3-C	-1008.96	-1013.15	0.18	-1012.97	-0.60
Ti-N3-Cu-N3-C	-1003.43	-1007.48	0.14	-1007.34	-0.50
V-N3-Co-N3-C	-1007.36	-1011.30	0.21	-1011.08	-0.31
V-N3-Cu-N3-C	-1003.92	-1007.69	0.19	-1007.50	-0.17
V-N3-Mn-N3-C	-1008.47	-1012.95	0.19	-1012.76	-0.88
V-N3-V-N3-C	-1008.70	-1013.20	0.18	-1013.01	-0.90
Zn-N3-Zn-N3-C	-993.99	-998.04	0.18	-997.86	-0.46
Au-N3-Co-N3-C	-1000.28	-1003.86	0.18	-1003.68	0.01

Table S7: Hydrogen adsorption energies of M_1-N_3-M_2-N_3-C

$*$	E_*	E_{H^*}	G_{corr}	G_{H^*}	ΔG_{H^*}
Au-N3-Mn-N3-C	-1002.54	-1005.86	0.13	-1005.73	0.22
Au-N3-Mo-N3-C	-1002.12	-1006.14	0.14	-1005.99	-0.46
Au-N3-Pd-N3-C	-998.35	-1001.20	0.20	-1001.00	0.76
Au-N3-Tc-N3-C	-1001.48	-1005.40	0.15	-1005.24	-0.35
Co-N3-Tc-N3-C	-1007.00	-1010.80	0.28	-1010.53	-0.12
Cr-N3-Au-N3-C	-1003.05	-1006.38	0.13	-1006.24	0.22
Cr-N3-Rh-N3-C	-1007.45	-1011.68	0.21	-1011.47	-0.62
Cu-N3-Pd-N3-C	-1000.01	-1003.33	0.21	-1003.11	0.31
Cu-N3-Re-N3-C	-1004.23	-1008.37	0.16	-1008.21	-0.57
Cu-N3-Ta-N3-C	-1005.03	-1009.65	0.16	-1009.49	-1.05
Fe-N3-Au-N3-C	-1001.30	-1004.63	0.19	-1004.44	0.27
Fe-N3-Os-N3-C	-1008.40	-1012.60	0.19	-1012.41	-0.60
Fe-N3-Re-N3-C	-1009.48	-1013.51	0.18	-1013.32	-0.43
Fe-N3-Rh-N3-C	-1005.96	-1009.98	0.21	-1009.77	-0.40
Fe-N3-Tc-N3-C	-1008.15	-1012.00	0.18	-1011.82	-0.26
Fe-N3-W-N3-C	-1010.06	-1014.38	0.17	-1014.21	-0.74
Ir-N3-Os-N3-C	-1007.96	-1012.28	0.20	-1012.08	-0.71
Ir-N3-Pd-N3-C	-1003.47	-1007.81	0.21	-1007.60	-0.72
Ir-N3-Rh-N3-C	-1005.65	-1009.40	0.22	-1009.18	-0.12
Ir-N3-Tc-N3-C	-1008.07	-1011.85	0.20	-1011.65	-0.17
Mo-N3-Os-N3-C	-1010.40	-1014.00	0.16	-1013.84	-0.03
Mo-N3-Rh-N3-C	-1007.42	-1011.54	0.17	-1011.37	-0.54
Nb-N3-Au-N3-C	-1002.35	-1006.73	0.14	-1006.59	-0.83
Nb-N3-Cu-N3-C	-1003.94	-1008.22	0.14	-1008.08	-0.73
Nb-N3-Fe-N3-C	-1008.86	-1012.75	0.22	-1012.53	-0.26
Nb-N3-Pd-N3-C	-1005.42	-1009.72	0.15	-1009.57	-0.74
Ni-N3-Au-N3-C	-999.32	-1002.18	0.19	-1001.98	0.75
Ni-N3-Nb-N3-C	-1006.46	-1010.71	0.15	-1010.56	-0.69
Ni-N3-Os-N3-C	-1005.58	-1009.78	0.19	-1009.59	-0.60
Ni-N3-Pd-N3-C	-1002.00	-1005.62	0.23	-1005.40	0.01
Ni-N3-Rh-N3-C	-1003.49	-1007.63	0.22	-1007.42	-0.52
Ni-N3-Tc-N3-C	-1005.48	-1009.34	0.22	-1009.12	-0.23
Os-N3-Co-N3-C	-1006.99	-1011.17	0.17	-1011.00	-0.60
Os-N3-Cr-N3-C	-1009.64	-1013.66	0.18	-1013.48	-0.44
Os-N3-Mn-N3-C	-1009.43	-1013.69	0.19	-1013.50	-0.66
Os-N3-Os-N3-C	-1009.76	-1013.77	0.20	-1013.58	-0.41
Os-N3-Pt-N3-C	-1005.84	-1010.06	0.20	-1009.86	-0.62
Os-N3-Rh-N3-C	-1006.89	-1011.09	0.20	-1010.89	-0.59
Os-N3-Ti-N3-C	-1009.26	-1013.87	0.20	-1013.67	-1.00
Os-N3-V-N3-C	-1009.81	-1014.03	0.19	-1013.83	-0.61
Pd-N3-Co-N3-C	-1002.86	-1006.90	0.22	-1006.68	-0.41
Pd-N3-Fe-N3-C	-1004.06	-1007.82	0.22	-1007.60	-0.14
Pd-N3-Mn-N3-C	-1005.45	-1008.98	0.21	-1008.78	0.08
Pd-N3-Mo-N3-C	-1005.24	-1009.30	0.16	-1009.13	-0.48
Pd-N3-Pd-N3-C	-1000.94	-1004.75	0.23	-1004.52	-0.17
Pd-N3-V-N3-C	-1005.03	-1008.84	0.22	-1008.63	-0.19
Pt-N3-Au-N3-C	-999.32	-1002.44	0.20	-1002.24	0.48
Pt-N3-Nb-N3-C	-1006.54	-1010.88	0.15	-1010.73	-0.78
Re-N3-Au-N3-C	-1002.55	-1006.84	0.15	-1006.69	-0.73
Re-N3-Co-N3-C	-1008.12	-1012.34	0.19	-1012.15	-0.62
Re-N3-Ir-N3-C	-1009.32	-1013.61	0.18	-1013.42	-0.69
Re-N3-Ni-N3-C	-1006.35	-1010.77	0.16	-1010.61	-0.85
Re-N3-Pt-N3-C	-1006.83	-1011.15	0.18	-1010.97	-0.73
Re-N3-Rh-N3-C	-1008.07	-1012.28	0.18	-1012.09	-0.61
Rh-N3-Co-N3-C	-1004.52	-1008.75	0.21	-1008.54	-0.61
Rh-N3-Cu-N3-C	-1001.79	-1005.44	0.19	-1005.26	-0.06
Rh-N3-Mn-N3-C	-1007.07	-1011.35	0.21	-1011.14	-0.67

Table S7: Hydrogen adsorption energies of M_1-N_3-M_2-N_3-C

$*$	E_*	E_{H^*}	G_{corr}	G_{H^*}	ΔG_{H^*}
Rh-N3-V-N3-C	-1007.03	-1011.21	0.21	-1011.00	-0.57
Ru-N3-Os-N3-C	-1008.41	-1012.42	0.19	-1012.23	-0.41
Tc-N3-Cu-N3-C	-1003.26	-1007.06	0.23	-1006.83	-0.16
Tc-N3-Mn-N3-C	-1009.13	-1013.18	0.22	-1012.96	-0.42
W-N3-Cu-N3-C	-1004.83	-1009.33	0.15	-1009.18	-0.94
Zn-N3-Pd-N3-C	-997.29	-1001.30	0.22	-1001.08	-0.38
Ir-N3-Ir-N3-C	-1006.78	-1010.47	0.17	-1010.30	-0.11
Zr-N3-Pd-N3-C	-1005.48	-1009.53	0.14	-1009.40	-0.51
Zr-N3-Mo-N3-C	-1009.10	-1013.79	0.18	-1013.61	-1.10
W-N3-Ni-N3-C	-1007.29	-1011.71	0.16	-1011.54	-0.84
W-N3-Co-N3-C	-1009.01	-1013.37	0.18	-1013.19	-0.77
Ti-N3-Au-N3-C	-1001.81	-1005.98	0.14	-1005.84	-0.63
Tc-N3-V-N3-C	-1009.36	-1013.70	0.20	-1013.50	-0.74
Tc-N3-Ti-N3-C	-1008.88	-1012.91	0.20	-1012.71	-0.42
Tc-N3-Tc-N3-C	-1009.58	-1013.21	0.17	-1013.04	-0.06
Tc-N3-Ru-N3-C	-1008.40	-1011.90	0.18	-1011.72	0.09
Tc-N3-Rh-N3-C	-1006.87	-1010.44	0.26	-1010.18	0.10
Tc-N3-Pd-N3-C	-1004.48	-1008.45	0.16	-1008.29	-0.41
Tc-N3-Os-N3-C	-1009.74	-1013.32	0.18	-1013.14	0.01
Tc-N3-Mo-N3-C	-1010.00	-1013.81	0.19	-1013.62	-0.21
Au-N3-Ta-N3-C	-1003.54	-1008.22	0.16	-1008.06	-1.11
Cd-N3-Ni-N3-C	-996.99	-1000.60	0.17	-1000.43	-0.03
Co-N3-Cd-N3-C	-998.14	-1002.00	0.18	-1001.82	-0.27
Co-N3-Nb-N3-C	-1007.99	-1011.66	0.18	-1011.48	-0.08
Cr-N3-Pd-N3-C	-1005.57	-1009.60	0.22	-1009.38	-0.40
Cr-N3-Tc-N3-C	-1009.55	-1013.73	0.20	-1013.53	-0.57
Cu-N3-Cd-N3-C	-995.11	-998.78	0.17	-998.61	-0.09
Fe-N3-Cd-N3-C	-999.33	-1003.03	0.17	-1002.86	-0.12
Hf-N3-Ni-N3-C	-1007.65	-1011.15	0.15	-1011.00	0.06
Hf-N3-Pd-N3-C	-1006.76	-1011.04	0.15	-1010.89	-0.72
Hf-N3-Pt-N3-C	-1007.85	-1010.92	0.19	-1010.72	0.54
Hf-N3-Ru-N3-C	-1009.89	-1013.59	0.19	-1013.39	-0.09
Ir-N3-Cd-N3-C	-999.15	-1003.05	0.17	-1002.88	-0.32
Ir-N3-Nb-N3-C	-1009.07	-1012.51	0.22	-1012.29	0.19
Mn-N3-Re-N3-C	-1010.39	-1014.64	0.16	-1014.48	-0.68
Mo-N3-Nb-N3-C	-1010.09	-1014.29	0.18	-1014.11	-0.61
Mo-N3-Ta-N3-C	-1010.59	-1015.61	0.19	-1015.42	-1.43
Mo-N3-W-N3-C	-1010.98	-1015.73	0.20	-1015.53	-1.14
Nb-N3-Nb-N3-C	-1009.85	-1014.47	0.18	-1014.30	-1.05
Nb-N3-Ti-N3-C	-1008.65	-1013.48	0.17	-1013.30	-1.24
Nb-N3-Zr-N3-C	-1009.08	-1013.90	0.16	-1013.74	-1.25
Os-N3-Cd-N3-C	-1000.29	-1004.51	0.20	-1004.31	-0.61
Os-N3-Hf-N3-C	-1010.93	-1014.68	0.19	-1014.49	-0.15
Pd-N3-Ta-N3-C	-1006.80	-1011.40	0.16	-1011.24	-1.03
Pd-N3-Ti-N3-C	-1004.90	-1008.84	0.15	-1008.70	-0.39
Pt-N3-Cd-N3-C	-997.10	-1000.66	0.21	-1000.45	0.06
Pt-N3-Tc-N3-C	-1005.60	-1009.55	0.16	-1009.39	-0.38
Pt-N3-Zr-N3-C	-1006.58	-1009.60	0.18	-1009.42	0.57
Pt-N3-W-N3-C	-1007.62	-1012.15	0.17	-1011.97	-0.94
Re-N3-Mo-N3-C	-1011.23	-1015.14	0.18	-1014.96	-0.32
Re-N3-Nb-N3-C	-1010.90	-1015.27	0.20	-1015.08	-0.77
Re-N3-Os-N3-C	-1011.16	-1014.92	0.19	-1014.74	-0.18
Re-N3-Re-N3-C	-1012.49	-1016.44	0.17	-1016.27	-0.37
Re-N3-Tc-N3-C	-1010.97	-1014.61	0.17	-1014.44	-0.06
Rh-N3-Cd-N3-C	-998.25	-1002.04	0.17	-1001.87	-0.21
Rh-N3-Pt-N3-C	-1003.44	-1007.74	0.23	-1007.51	-0.66
Rh-N3-Rh-N3-C	-1004.59	-1008.25	0.19	-1008.06	-0.06

Table S7: Hydrogen adsorption energies of M_1-N_3-M_2-N_3-C

$*$	E_*	E_{H^*}	G_{corr}	G_{H^*}	ΔG_{H^*}
Rh-N3-Ru-N3-C	-1005.72	-1009.36	0.34	-1009.02	0.11
Rh-N3-Ti-N3-C	-1007.02	-1011.14	0.21	-1010.93	-0.50
Rh-N3-W-N3-C	-1008.85	-1013.29	0.18	-1013.11	-0.86
Ru-N3-Au-N3-C	-1000.65	-1004.74	0.18	-1004.56	-0.50
Ru-N3-Cd-N3-C	-999.25	-1003.29	0.19	-1003.11	-0.45
Ru-N3-Pd-N3-C	-1003.60	-1007.41	0.18	-1007.23	-0.22
Ru-N3-Re-N3-C	-1009.69	-1013.84	0.19	-1013.65	-0.55
Ru-N3-Zr-N3-C	-1008.80	-1012.34	0.19	-1012.15	0.05
Ta-N3-Ni-N3-C	-1007.78	-1012.36	0.16	-1012.20	-1.01
Ta-N3-Pt-N3-C	-1007.97	-1012.62	0.16	-1012.45	-1.08
Ta-N3-Ti-N3-C	-1009.59	-1014.69	0.18	-1014.51	-1.51
Tc-N3-Zr-N3-C	-1008.95	-1013.46	0.19	-1013.27	-0.92
Ti-N3-Hf-N3-C	-1009.10	-1013.95	0.14	-1013.81	-1.30
W-N3-Au-N3-C	-1003.25	-1007.72	0.15	-1007.57	-0.91
W-N3-Pd-N3-C	-1006.45	-1010.92	0.17	-1010.75	-0.89
W-N3-V-N3-C	-1009.74	-1014.83	0.20	-1014.63	-1.48
Zr-N3-Fe-N3-C	-1008.43	-1012.30	0.17	-1012.13	-0.29
Zr-N3-Ni-N3-C	-1006.45	-1009.87	0.17	-1009.70	0.16
Zr-N3-Os-N3-C	-1009.84	-1013.48	0.18	-1013.29	-0.04
Pd-N3-Re-N3-C	-1005.62	-1009.93	0.15	-1009.78	-0.75
Zr-N3-Ti-N3-C	-1007.87	-1012.74	0.16	-1012.58	-1.30

Table S8: Hydrogen adsorption energies of M_1-N_4-M_2-N_4-C

$*$	E_*	E_{H^*}	G_{corr}	G_{H^*}	ΔG_{H^*}
Co-N4-Au-N4-C	-999.72	-1003.03	0.20	-1002.84	0.29
Co-N4-Co-N4-C	-1006.45	-1009.28	0.20	-1009.08	0.78
Co-N4-Cu-N4-C	-1001.34	-1004.47	0.20	-1004.27	0.48
Co-N4-Ni-N4-C	-1004.52	-1007.67	0.20	-1007.47	0.46
Co-N4-Os-N4-C	-1009.48	-1013.33	0.21	-1013.13	-0.24
Co-N4-Pd-N4-C	-1003.34	-1006.38	0.20	-1006.18	0.57
Co-N4-Pt-N4-C	-1004.47	-1007.53	0.20	-1007.33	0.55
Cr-N4-Hf-N4-C	-1012.85	-1016.63	0.20	-1016.43	-0.17
Co-N4-Rh-N4-C	-1006.15	-1008.66	0.20	-1008.46	1.10
Co-N4-Ru-N4-C	-1007.89	-1011.65	0.21	-1011.45	-0.15
Cr-N4-Co-N4-C	-1009.91	-1013.35	0.19	-1013.16	0.16
Cr-N4-Cr-N4-C	-1012.13	-1015.78	0.18	-1015.60	-0.06
Cr-N4-Fe-N4-C	-1010.99	-1014.48	0.20	-1014.28	0.12
Cr-N4-Ir-N4-C	-1010.93	-1014.20	0.20	-1014.00	0.34
Cr-N4-Mn-N4-C	-1012.02	-1015.67	0.19	-1015.48	-0.05
Cr-N4-Ni-N4-C	-1008.03	-1009.78	0.14	-1009.65	1.79
Cr-N4-Os-N4-C	-1012.63	-1016.54	0.20	-1016.34	-0.30
Cr-N4-Rh-N4-C	-1009.59	-1012.68	0.20	-1012.48	0.52
Cr-N4-Ru-N4-C	-1010.97	-1014.86	0.20	-1014.66	-0.28
Fe-N4-Co-N4-C	-1007.89	-1011.25	0.21	-1011.04	0.26
Fe-N4-Cu-N4-C	-1002.42	-1006.03	0.20	-1005.83	-0.00
Fe-N4-Fe-N4-C	-1009.36	-1012.63	0.21	-1012.43	0.34
Fe-N4-Ir-N4-C	-1008.89	-1011.95	0.21	-1011.74	0.56
Fe-N4-Ni-N4-C	-1005.66	-1009.18	0.20	-1008.98	0.09
Fe-N4-Os-N4-C	-1010.91	-1014.76	0.21	-1014.55	-0.23
Fe-N4-Pd-N4-C	-1004.59	-1008.00	0.20	-1007.80	0.20
Fe-N4-Pt-N4-C	-1005.78	-1009.14	0.20	-1008.93	0.26
Fe-N4-Re-N4-C	-1012.26	-1016.26	0.19	-1016.07	-0.40
Fe-N4-Rh-N4-C	-1007.59	-1010.93	0.21	-1010.72	0.28
Fe-N4-Ru-N4-C	-1009.35	-1013.08	0.21	-1012.87	-0.11

Table S8: Hydrogen adsorption energies of M_1-N_4-M_2-N_4-C

*	E_*	E_{H^*}	G_{corr}	G_{H^*}	ΔG_{H^*}
Fe-N4-Tc-N4-C	-1010.63	-1013.80	0.21	-1013.59	0.45
Mn-N4-Cu-N4-C	-1003.86	-1005.83	0.13	-1005.70	1.57
Mn-N4-Fe-N4-C	-1010.63	-1014.02	0.20	-1013.82	0.22
Mn-N4-Ir-N4-C	-1010.39	-1013.27	0.20	-1013.07	0.73
Mn-N4-Mn-N4-C	-1011.73	-1015.13	0.19	-1014.94	0.20
Mn-N4-Ni-N4-C	-1007.24	-1009.21	0.15	-1009.05	1.60
Mn-N4-Os-N4-C	-1012.26	-1015.50	0.20	-1015.29	0.38
Mn-N4-Pd-N4-C	-1006.03	-1009.47	0.21	-1009.27	0.17
Mn-N4-Rh-N4-C	-1009.09	-1011.77	0.20	-1011.58	0.92
Mn-N4-Ru-N4-C	-1010.66	-1014.42	0.20	-1014.22	-0.15
Mn-N4-Tc-N4-C	-1011.75	-1014.97	0.19	-1014.78	0.38
Ni-N4-Ni-N4-C	-1003.03	-1005.19	0.17	-1005.02	1.42
Ni-N4-Os-N4-C	-1007.26	-1010.91	0.20	-1010.71	-0.04
Ni-N4-Re-N4-C	-1008.66	-1012.83	0.20	-1012.63	-0.56
Ni-N4-Ru-N4-C	-1005.79	-1009.24	0.20	-1009.04	0.16
Ni-N4-Tc-N4-C	-1007.07	-1011.15	0.20	-1010.95	-0.47
Os-N4-Ir-N4-C	-1010.36	-1014.29	0.21	-1014.08	-0.31
Os-N4-Os-N4-C	-1012.42	-1016.35	0.21	-1016.14	-0.31
Os-N4-Pt-N4-C	-1007.25	-1010.90	0.20	-1010.70	-0.04
Rh-N4-Os-N4-C	-1009.12	-1012.01	0.21	-1011.81	0.72
Rh-N4-Re-N4-C	-1010.49	-1014.76	0.20	-1014.57	-0.67
Rh-N4-Rh-N4-C	-1005.84	-1008.46	0.20	-1008.26	0.99
Ru-N4-Os-N4-C	-1010.86	-1014.65	0.21	-1014.44	-0.17
Ru-N4-Pt-N4-C	-1005.84	-1009.31	0.20	-1009.11	0.14
Ru-N4-Rh-N4-C	-1007.55	-1011.33	0.21	-1011.13	-0.17
Ru-N4-Ru-N4-C	-1009.26	-1013.05	0.21	-1012.85	-0.18
Tc-N4-Rh-N4-C	-1008.87	-1012.97	0.19	-1012.78	-0.50
V-N4-Co-N4-C	-1009.60	-1013.34	0.17	-1013.17	-0.16
V-N4-Fe-N4-C	-1010.82	-1014.12	0.20	-1013.92	0.31
V-N4-Ni-N4-C	-1007.90	-1011.72	0.17	-1011.55	-0.24
Mn-N4-Pt-N4-C	-1007.22	-1010.64	0.21	-1010.44	0.19
Tc-N4-Os-N4-C	-1012.14	-1016.01	0.20	-1015.81	-0.26
V-N4-Rh-N4-C	-1009.31	-1012.55	0.20	-1012.35	0.37
Co-N4-Tc-N4-C	-1009.24	-1013.25	0.20	-1013.05	-0.40
Pd-N4-Os-N4-C	-1006.16	-1009.79	0.20	-1009.59	-0.02
Cu-N4-Os-N4-C	-1004.21	-1008.02	0.20	-1007.82	-0.20
Ni-N4-Ir-N4-C	-1005.02	-1008.52	0.20	-1008.32	0.11
Cr-N4-Pt-N4-C	-1008.21	-1010.04	0.15	-1009.89	1.73
V-N4-Ir-N4-C	-1010.63	-1014.13	0.20	-1013.93	0.11
Ru-N4-Pd-N4-C	-1004.71	-1008.17	0.20	-1007.97	0.15
Co-N4-Ir-N4-C	-1007.42	-1010.36	0.20	-1010.16	0.67
V-N4-Cu-N4-C	-1004.68	-1006.41	0.13	-1006.28	1.81
Ir-N4-Ir-N4-C	-1008.27	-1011.16	0.21	-1010.95	0.73
Ir-N4-Pt-N4-C	-1004.88	-1007.41	0.19	-1007.22	1.07
Cr-N4-Tc-N4-C	-1011.97	-1016.10	0.19	-1015.91	-0.53
Hf-N4-Hf-N4-C	-1013.31	-1017.90	0.17	-1017.73	-1.01
Hf-N4-Ta-N4-C	-1013.99	-1018.55	0.17	-1018.39	-0.99
Mn-N4-Ta-N4-C	-1013.53	-1018.23	0.17	-1018.06	-1.12
Mn-N4-W-N4-C	-1013.94	-1018.54	0.19	-1018.36	-1.01
Mn-N4-Zn-N4-C	-1001.73	-1004.94	0.18	-1004.76	0.38
Mo-N4-Hf-N4-C	-1012.72	-1017.19	0.19	-1017.00	-0.87
Mo-N4-Ir-N4-C	-1011.06	-1015.24	0.18	-1015.06	-0.59
Mo-N4-Os-N4-C	-1012.68	-1016.78	0.17	-1016.61	-0.52
Mo-N4-Pd-N4-C	-1006.91	-1011.08	0.18	-1010.90	-0.58
Mo-N4-Pt-N4-C	-1008.08	-1012.24	0.18	-1012.07	-0.58
Mo-N4-Rh-N4-C	-1009.76	-1013.95	0.18	-1013.77	-0.60
Mo-N4-Ru-N4-C	-1011.11	-1015.17	0.17	-1015.00	-0.48

Table S8: Hydrogen adsorption energies of M_1-N_4-M_2-N_4-C

$*$	E_*	E_{H^*}	G_{corr}	G_{H^*}	ΔG_{H^*}
Mo-N4-Ta-N4-C	-1013.93	-1018.19	0.19	-1018.00	-0.66
Mo-N4-Tc-N4-C	-1012.34	-1016.35	0.17	-1016.17	-0.42
Mo-N4-W-N4-C	-1014.24	-1018.21	0.31	-1017.91	-0.26
Nb-N4-Mo-N4-C	-1012.63	-1016.77	0.30	-1016.47	-0.43
Nb-N4-Nb-N4-C	-1012.34	-1016.90	0.18	-1016.72	-0.97
Nb-N4-Ta-N4-C	-1013.64	-1018.29	0.19	-1018.11	-1.06
Ta-N4-Ta-N4-C	-1014.86	-1019.59	0.19	-1019.40	-1.13
Tc-N4-Pd-N4-C	-1006.04	-1010.11	0.19	-1009.92	-0.47
Tc-N4-Tc-N4-C	-1011.81	-1015.70	0.18	-1015.52	-0.30
Ti-N4-Mo-N4-C	-1011.30	-1015.56	0.27	-1015.29	-0.58
Ti-N4-Nb-N4-C	-1011.08	-1015.70	0.18	-1015.51	-1.02
Ti-N4-Re-N4-C	-1012.72	-1016.88	0.19	-1016.69	-0.56
Ti-N4-Ta-N4-C	-1012.52	-1017.13	0.17	-1016.96	-1.03
Ti-N4-Ti-N4-C	-1010.15	-1014.51	0.18	-1014.33	-0.77
V-N4-Mn-N4-C	-1011.68	-1015.66	0.17	-1015.49	-0.40
V-N4-Re-N4-C	-1013.36	-1017.43	0.19	-1017.24	-0.47
V-N4-Tc-N4-C	-1011.74	-1015.65	0.18	-1015.47	-0.32
V-N4-V-N4-C	-1011.28	-1015.46	0.19	-1015.28	-0.59
W-N4-W-N4-C	-1016.08	-1020.51	0.18	-1020.33	-0.84
Zr-N4-Hf-N4-C	-1012.01	-1016.62	0.17	-1016.45	-1.03
Zr-N4-Nb-N4-C	-1011.40	-1016.10	0.18	-1015.92	-1.11
Zr-N4-Zr-N4-C	-1010.70	-1015.32	0.17	-1015.15	-1.04
W-N4-Os-N4-C	-1014.41	-1018.84	0.18	-1018.66	-0.84
V-N4-Ru-N4-C	-1010.66	-1014.60	0.20	-1014.40	-0.33
V-N4-Os-N4-C	-1012.22	-1016.24	0.19	-1016.05	-0.42
Ti-N4-Tc-N4-C	-1010.92	-1014.60	0.15	-1014.45	-0.12
Ta-N4-Os-N4-C	-1014.13	-1018.67	0.17	-1018.50	-0.96
Ru-N4-W-N4-C	-1012.84	-1017.26	0.18	-1017.08	-0.83
Rh-N4-Ta-N4-C	-1011.28	-1015.90	0.17	-1015.73	-1.04
Nb-N4-Tc-N4-C	-1012.24	-1016.40	0.16	-1016.25	-0.60
Nb-N4-Os-N4-C	-1012.75	-1016.65	0.19	-1016.46	-0.30
Mn-N4-Co-N4-C	-1009.38	-1012.64	0.20	-1012.44	0.35
Ni-N4-Rh-N4-C	-1003.87	-1007.24	0.20	-1007.04	0.24
Mn-N4-Nb-N4-C	-1012.17	-1016.51	0.16	-1016.35	-0.77
Mn-N4-Mo-N4-C	-1012.21	-1016.49	0.17	-1016.32	-0.70
Fe-N4-Nb-N4-C	-1011.21	-1015.39	0.16	-1015.23	-0.61
Hf-N4-Os-N4-C	-1013.25	-1017.27	0.14	-1017.13	-0.47
Fe-N4-Mo-N4-C	-1011.15	-1015.28	0.17	-1015.11	-0.55
Ni-N4-Pt-N4-C	-1002.85	-1005.45	0.18	-1005.27	0.99
Ni-N4-Nb-N4-C	-1008.16	-1012.57	0.16	-1012.41	-0.84
Ni-N4-Mo-N4-C	-1007.84	-1012.03	0.18	-1011.85	-0.60
Co-N4-Nb-N4-C	-1009.93	-1014.22	0.16	-1014.06	-0.72
Co-N4-Mo-N4-C	-1009.91	-1014.11	0.18	-1013.93	-0.61
Cr-N4-Cu-N4-C	-1004.83	-1006.57	0.13	-1006.43	1.81
Cr-N4-Mo-N4-C	-1012.25	-1016.58	0.17	-1016.41	-0.75
Cr-N4-Re-N4-C	-1013.58	-1017.52	0.19	-1017.33	-0.34
Nb-N4-Re-N4-C	-1013.90	-1017.93	0.19	-1017.74	-0.43
Nb-N4-Ir-N4-C	-1011.13	-1015.36	0.15	-1015.20	-0.66
Nb-N4-Ru-N4-C	-1011.20	-1015.05	0.19	-1014.86	-0.25
Nb-N4-Rh-N4-C	-1009.85	-1012.95	0.19	-1012.76	0.50
Cu-N4-Mo-N4-C	-1004.85	-1009.04	0.18	-1008.86	-0.60
Nb-N4-Pt-N4-C	-1008.49	-1012.88	0.16	-1012.72	-0.82
Ru-N4-Ta-N4-C	-1012.60	-1016.50	0.19	-1016.30	-0.29
Cr-N4-Nb-N4-C	-1012.27	-1016.49	0.15	-1016.34	-0.66
Ti-N4-Co-N4-C	-1009.06	-1012.25	0.17	-1012.09	0.38
Ti-N4-Cu-N4-C	-1004.28	-1008.10	0.15	-1007.95	-0.26
Ti-N4-Ru-N4-C	-1010.17	-1014.03	0.19	-1013.84	-0.26

Table S8: Hydrogen adsorption energies of M_1-N_4-M_2-N_4-C

$*$	E_*	E_{H^*}	G_{corr}	G_{H^*}	ΔG_{H^*}
Ti-N4-Fe-N4-C	-1010.26	-1013.59	0.19	-1013.41	0.26
V-N4-Cr-N4-C	-1011.65	-1015.54	0.16	-1015.38	-0.32
Fe-N4-Ta-N4-C	-1012.59	-1017.26	0.17	-1017.09	-1.09
Ti-N4-Ir-N4-C	-1010.21	-1013.63	0.19	-1013.43	0.19
Ti-N4-Os-N4-C	-1011.76	-1015.63	0.18	-1015.45	-0.28
Tc-N4-Ta-N4-C	-1013.46	-1018.13	0.17	-1017.96	-1.09
V-N4-Mo-N4-C	-1012.08	-1016.20	0.17	-1016.03	-0.54
Ti-N4-Pt-N4-C	-1007.74	-1011.48	0.15	-1011.33	-0.18
Co-N4-Ta-N4-C	-1011.37	-1016.03	0.17	-1015.86	-1.08
Co-N4-W-N4-C	-1011.68	-1014.29	0.18	-1014.11	0.98
Cr-N4-W-N4-C	-1013.89	-1018.56	0.18	-1018.38	-1.08
Co-N4-Hf-N4-C	-1010.57	-1013.98	0.16	-1013.82	0.16
V-N4-Pt-N4-C	-1008.10	-1010.05	0.17	-1009.88	1.63
Cr-N4-Pd-N4-C	-1006.99	-1008.59	0.12	-1008.48	1.92
Cu-N4-Nb-N4-C	-1005.13	-1009.53	0.16	-1009.37	-0.83
Mn-N4-Re-N4-C	-1013.46	-1014.65	0.18	-1014.46	2.41
W-N4-Re-N4-C	-1015.43	-1019.73	0.18	-1019.55	-0.71
Tc-N4-Re-N4-C	-1013.43	-1017.26	0.19	-1017.07	-0.23
Cu-N4-Re-N4-C	-1005.61	-1009.88	0.20	-1009.68	-0.66
Ru-N4-Re-N4-C	-1012.24	-1016.46	0.20	-1016.26	-0.61
Pd-N4-Re-N4-C	-1007.54	-1011.82	0.20	-1011.62	-0.67
Mo-N4-Mo-N4-C	-1012.54	-1016.84	0.17	-1016.67	-0.72
Fe-N4-W-N4-C	-1012.94	-1017.38	0.18	-1017.20	-0.85
Rh-N4-Ir-N4-C	-1007.08	-1009.98	0.20	-1009.77	0.72
Tc-N4-Ir-N4-C	-1010.13	-1014.26	0.20	-1014.06	-0.52
Ni-N4-Cu-N4-C	-999.89	-1001.61	0.12	-1001.49	1.81
Co-N4-Re-N4-C	-1010.82	-1014.99	0.20	-1014.79	-0.56
Cu-N4-W-N4-C	-1006.58	-1011.14	0.19	-1010.95	-0.96
Re-N4-Re-N4-C	-1015.00	-1019.07	0.19	-1018.88	-0.47
Ta-N4-Ir-N4-C	-1012.54	-1016.01	0.20	-1015.81	0.14
Cr-N4-Ta-N4-C	-1013.54	-1017.51	0.20	-1017.31	-0.36
Pd-N4-Pt-N4-C	-1001.34	-1003.65	0.19	-1003.46	1.29
Cu-N4-Ru-N4-C	-1002.69	-1006.28	0.18	-1006.11	-0.01
Ru-N4-Ir-N4-C	-1008.83	-1012.63	0.21	-1012.42	-0.18
Ni-N4-Pd-N4-C	-1001.70	-1003.91	0.17	-1003.74	1.37
Ni-N4-W-N4-C	-1009.54	-1014.08	0.19	-1013.89	-0.94
Mn-N4-Hf-N4-C	-1012.58	-1016.22	0.16	-1016.06	-0.07
Zr-N4-Au-N4-C	-1002.82	-1007.27	0.14	-1007.13	-0.90

References

[1] Georg Kresse and Jürgen Furthmüller. Efficient iterative schemes for ab initio total-energy calculations using a plane-wave basis set. *Physical review B*, 54(16):11169, 1996.

[2] Georg Kresse and Jürgen Furthmüller. Efficiency of ab-initio total energy calculations for metals and semiconductors using a plane-wave basis set. *Computational materials science*, 6(1):15–50, 1996.

[3] John P Perdew, Kieron Burke, and Matthias Ernzerhof. Generalized gradient approximation made simple. *Physical review letters*, 77(18):3865, 1996.

[4] Georg Kresse and Daniel Joubert. From ultrasoft pseudopotentials to the projector augmented-wave method. *Physical review b*, 59(3):1758, 1999.

[5] Stefan Grimme, Jens Antony, Stephan Ehrlich, and Helge Krieg. A consistent and accurate ab initio parametrization of density functional dispersion correction (dft-d) for the 94 elements h-pu. *The Journal of chemical physics*, 132(15), 2010.

[6] Volker L Deringer, Andrei L Tchougréeff, and Richard Dronskowski. Crystal orbital hamilton population (cohp) analysis as projected from plane-wave basis sets. *The journal of physical chemistry A*, 115(21):5461–5466, 2011.

[7] Stefan Maintz, Volker L Deringer, Andrei L Tchougréeff, and Richard Dronskowski. Lobster: a tool to extract chemical bonding from plane-wave based dft, 2016.

[8] Koichi Momma and Fujio Izumi. Vesta 3 for three-dimensional visualization of crystal, volumetric and morphology data. *Applied Crystallography*, 44(6):1272–1276, 2011.

[9] Vei Wang, Nan Xu, Jin-Cheng Liu, Gang Tang, and Wen-Tong Geng. Vaspkit: A user-friendly interface facilitating high-throughput computing and analysis using vasp code. *Computer Physics Communications*, 267:108033, 2021.
